# Artificial intelligence for individualized treatment of persistent atrial fibrillation: a randomized controlled trial

**DOI:** 10.1038/s41591-025-03517-w

**Published:** 2025-02-14

**Authors:** Isabel Deisenhofer, Jean-Paul Albenque, Sonia Busch, Edouard Gitenay, Stavros E. Mountantonakis, Antoine Roux, Jerome Horvilleur, Babe Bakouboula, Saumil Oza, Selim Abbey, Guillaume Theodore, Antoine Lepillier, Yves Guyomar, Francis Bessiere, Jaap Jan Smit, Theophile Mohr Durdez, Paola Milpied, Anthony Appetiti, Daniel Guerrero, Tom De Potter, Christian De Chillou, Seth Goldbarg, Atul Verma, John D. Hummel, TAILORED-AF Investigators, Isabel Deisenhofer, Jean-Paul Albenque, Sonia Busch, Edouard Gitenay, Stavros E. Mountantonakis, Antoine Roux, Jerome Horvilleur, Babe Bakouboula, Saumil Oza, Selim Abbey, Guillaume Theodore, Antoine Lepillier, Yves Guyomar, Francis Bessiere, Seth Goldbarg, John D. Hummel, Jaap Jan Smit, Tom De Potter, Christian De Chillou

**Affiliations:** 1 Department of Electrophysiology, German Heart Center Munich, Technical University of Munich (TUM) School of Medicine and Health, Munich, Germany; 2 Clinique Pasteur, Toulouse, France; 3 Department of Electrophysiology, Herz-Zentrum Bodensee, Constance, Germany; 4 Hopital Saint Joseph, Marseille, France; 5 Northwell Health, New York, NY USA; 6 Pôle Santé République, Clermont-Ferrand, France; 7 Hôpital Privé Jacques Cartier, Massy, France; 8 Clinique Rhena, Strasbourg, France; 9 Ascension St. Vincent’s Riverside Hospital, Jacksonville, FL USA; 10 Hôpital privé Le Confluent, Nantes, France; 11 Clinique Saint George, Nice, France; 12 Centre Cardiologique du Nord, Saint-Denis, France; 13 Hôpital Saint Philibert, Lomme, France; 14 Hopital Cardiologique Louis Pradel, Institut Cardiologique de Lyon, Hospices Civils de Lyon, Lyon, France; 15 Isala Klinieken, Zwolle, The Netherlands; 16 Volta Medical, Marseille, France; 17 Cardiovascular Center, Onze Lieve Vrouwziekenhuis Hospital, Aalst, Belgium; 18 Département de Cardiologie, CHU de Nancy, Nancy, France; 19 Division of Cardiology, New York-Presbyterian Queens, New York, NY USA; 20 McGill University Health Centre, Montreal, Quebec Canada; 21 The Ohio State University Wexner Medical Center, Columbus, OH USA

**Keywords:** Atrial fibrillation, Cardiac device therapy, Randomized controlled trials

## Abstract

Although pulmonary vein isolation (PVI) has become the cornerstone ablation procedure for atrial fibrillation (AF), the optimal ablation procedure for persistent and long-standing persistent AF remains elusive. Targeting spatio-temporal electrogram dispersion in a tailored procedure has been suggested as a potentially beneficial alternative to a conventional PVI-only procedure. In this multicenter, randomized, controlled, double-blind, superiority trial, patients with drug-refractory persistent AF were randomly assigned to a tailored ablation procedure targeting areas of spatio-temporal dispersion, as detected by an artificial intelligence (AI) algorithm, in addition to PVI (tailored arm, *n* = 187, 23% women) or to a conventional PVI-only procedure (anatomical arm, *n* = 183, 19% women). The primary efficacy endpoint was freedom from documented AF with or without antiarrhythmic drugs at 12 months after a single ablation procedure. Secondary endpoints included freedom from any atrial arrhythmic events, and the secondary composite safety endpoint consisted of death, cerebrovascular events, or treatment-related serious adverse events. One year post-procedure, the trial met its primary efficacy endpoint, which was achieved in 88% of patients in the tailored arm compared with 70% of patients in the anatomical arm (log-rank *P* < 0.0001 for superiority). However, no significant difference between arms was observed for the freedom from any atrial arrhythmia endpoint after one ablation. The safety endpoint did not differ between arms, with procedure and ablation times being twice as long in the tailored arm. These results show that AI-guided ablation of spatio-temporal dispersion areas in addition to PVI is superior to PVI alone in eliminating AF at 1-year follow-up in patients with persistent and long-standing persistent AF. Ablation of subsequent organized atrial tachycardias may be needed to maintain sinus rhythm long term. ClinicalTrials.gov identifier: NCT04702451.

## Abstract (web summary)

In a randomized controlled trial in individuals with persistent atrial fibrillation, an individualized ablation procedure, in which areas with abnormal electrophysiological characteristics—as detected by an AI algorithm—were targeted for ablation, led to improved efficacy for reducing arrhythmia recurrence at 12 months following the ablation procedure.

## Main

Atrial fibrillation (AF) is the most common cardiac arrhythmia in adults, affecting more than 33 million people worldwide and resulting in substantial healthcare costs^1–3^. Catheter-based ablation using an anatomically driven, standardized PVI is an effective treatment for patients with drug-refractory paroxysmal AF^3,4^. However, managing persistent and long-standing persistent AF remains challenging^5^. Long-term success after anatomical PVI alone is obtained in only about half of patients^6–9^.

Previous studies investigating ‘PVI plus’ ablation methods, such as PVI plus standardized anatomically defined atrial areas (for instance, the posterior wall of the left atrium) or PVI plus targeting magnetic resonance imaging (MRI)-detected areas of atrial fibrosis, have failed to demonstrate a clear advantage over a more comprehensive ablation strategy^8,9^. Similarly, personalized procedures based on an individual’s dynamic electrical patterns during ongoing AF did not show superiority over a PVI-only procedure^6^. A key challenge in incorporating electrogram-based ablation strategies into larger trials has been the need to ensure objectivity, consistency, and most importantly, reproducibility in the detection and adjudication of intracardiac electrograms across different physicians and centers.

Recent preliminary work has suggested that AI-driven, real-time software that can recognize and assign specific AF electrical patterns, specifically spatio-temporal electrogram dispersion, might overcome these challenges^10,11^. Spatio-temporal dispersion is defined as an ensemble of intracardiac electrograms forming a localized sequential activation in a distinct area, in which clusters of three or more adjacent bipolar electrograms show an intracardiac activation spanning the entire AF cycle length. This pattern suggests localized reentrant-like conduction, indicative of a role in initiating or maintaining AF^12^.

The purpose of the TAILORED-AF randomized controlled trial was to evaluate whether a tailored cardiac-ablation procedure targeting AI-detected areas harboring spatio-temporal dispersion, in addition to PVI, is more effective than an anatomical PVI-only procedure in patients with persistent and long-standing persistent AF.

## Results

### Algorithm performance and testing

On the single-annotated electrogram testing dataset, the algorithm achieved high performance, achieving a receiver operating characteristic (ROC) area under the curve (AUC) score of 0.94 (Supplementary Fig. 1). In multi-annotation mode, the median probability output of the dispersion algorithm was very low when all experts agreed that electrograms were not dispersed. It increased progressively with agreement levels, and a high probability output was reached when all five experts agreed that the electrograms were dispersed (the probabilities were 0.02, 0.42, 0.60, 0.74, 0.78 and 0.83 when 0, 1, 2, 3, 4 and 5 experts identified dispersion, respectively).

### Study participants

**Fig. 1:**
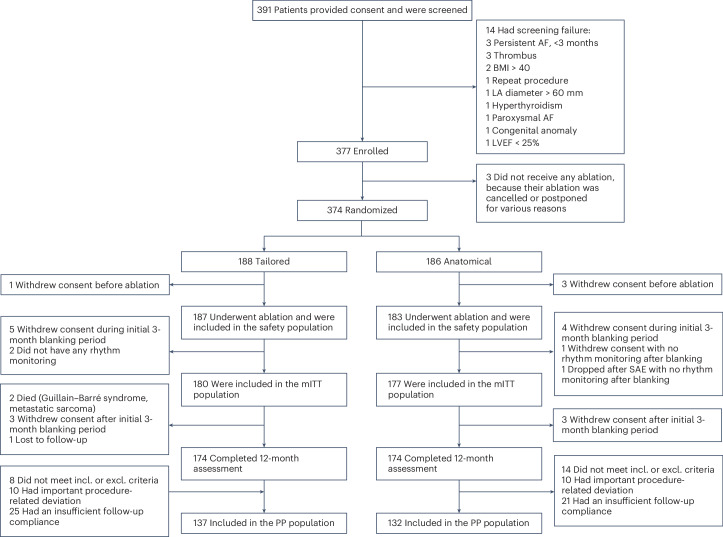
Participant flow diagram. Of the 374 patients randomized to either treatment, 188 were assigned to a tailored cardiac ablation procedure and 186 were assigned to a standard-of-care PVI procedure. Before the ablation procedure, one patient in the tailored arm and three patients in the anatomical arm withdrew their consent. In addition, seven patients in the tailored arm and six patients in the anatomical arm withdrew before the end of the blanking period. The primary analysis in the mITT population consisted of 180 patients and 177 patients in the tailored and anatomical arms, respectively. Of the 357 patients in the mITT population, 43 and 45 were excluded because of important protocol deviations in the tailored and anatomical arms, respectively. A total of 137 and 132 patients were included in the PP population in the tailored and anatomical arms, respectively. BMI, body mass index; LA, left atrium; LVEF, left ventricular ejection fraction; SAE, serious adverse event; incl., inclusion; excl., exclusion.

**Extended Data Fig. 1:**
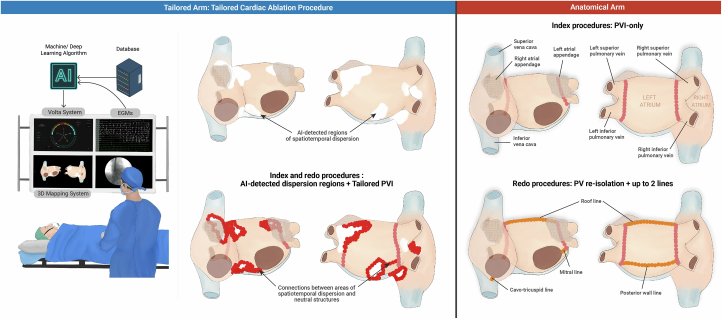
The Artificial Intelligence-Guided Ablation Strategy vs. the Standard-Of-Care Pulmonary Vein Isolation for Persistent Atrial Fibrillation. Patients randomly assigned to the Artificial Intelligence-guided Tailored Cardiac Ablation procedure (Tailored arm) had both right and left atria mapped with the Volta system where spatio-temporal dispersion was identified with the artificial intelligence classifier analyzing intra-cardiac electrograms. During a tailored procedure, dispersion areas (white regions) were ablated until atrial fibrillation terminated to sinus rhythm or until all dispersed areas were completely eliminated and/or isolated (red tags). Pulmonary vein isolation was then completed (pink tags). Repeat procedures had to be carried out using that same workflow. For the anatomical arm, antral pulmonary vein isolation only (pink tags) was performed during index procedure until bidirectional block had been achieved. During a repeat procedure, patients were allowed to undergo re-isolation of the pulmonary veins plus up to two additional anatomical lines of ablation (orange tags).

**Extended Data Fig. 2:**
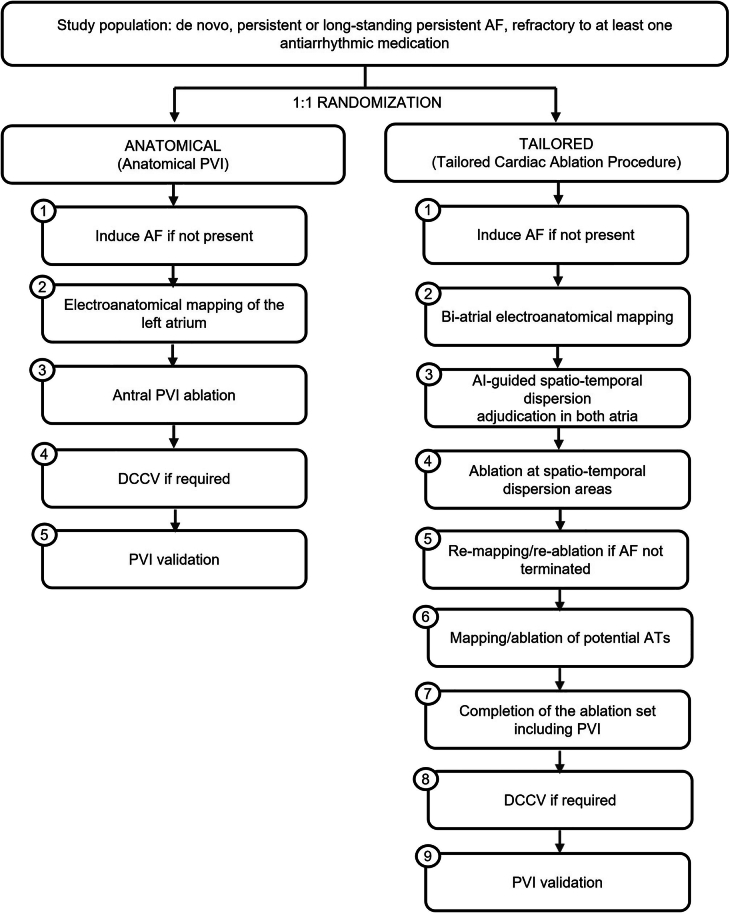
Anatomical PVI arm and AI-guided tailored cardiac ablation arm procedure steps. AF, atrial fibrillation; AT, atrial tachycardia; DCCV, direct current cardioversion; PVI, pulmonary vein isolation.

From February 2021 to December 2022, 391 patients were screened for the trial, and 377 were enrolled. A total of 370 patients underwent catheter ablation by 51 operators at 26 centers in 5 countries in Europe and the United States (Supplementary Table 1). Patients were randomly assigned (in a 1:1 ratio) to a tailored cardiac ablation procedure including PVI (tailored arm, 187 patients) or a PVI-only procedure (anatomical arm, 183 patients) (Fig. 1 and Extended Data Figs. 1 and 2). Baseline characteristics were well-balanced between the two groups (Table 1). A prespecified interim analysis, performed in January 2023, indicated that no increase in sample size was required.

**Table 1:** Baseline patient characteristics. CHA_2_DS_2_-VASc scores (an assessment of the risk of stroke among patients with AF) range from 0 to 9, with higher scores indicating a higher risk of stroke

Characteristic^a^	Overall (*n* = 370)	Tailored (*n* = 187)	Anatomical (*n* = 183)
Age (years)	65.7 ± 8.5	66.4 ± 8.5	64.9 ± 8.5
Sex, female	77 (21%)	42 (23%)	35 (19%)
Medical history			
Hypertension^b^	227 (61%)	118 (63%)	109 (60%)
Obesity, BMI > 30	156 (43%)	78 (42%)	78 (43%)
Hypercholesterolemia^c^	120 (32%)	62 (33%)	58 (32%)
Sleep apnea^d^	83 (22%)	39 (21%)	44 (25%)
Diabetes^b^	62 (17%)	33 (18%)	29 (16%)
Vascular disease^b^	55 (15%)	27 (14%)	28 (15%)
Prior stroke or TIA	27 (7%)	15 (8%)	12 (7%)
Cardiac implantable device^e^	21 (6%)	8 (4%)	13 (7%)
LVEF (%)^f^	54.8 ± 9.5	55.3 ± 9.3	54.3 ± 9.6
NYHA functional class^g^			
Class I	71 (25%)	33 (22%)	38 (27%)
Class II	166 (58%)	88 (60%)	78 (56%)
Class III	50 (17%)	26 (18%)	24 (17%)
Cardiomyopathy^c^	72 (19%)	32 (17%)	40 (22%)
Ischemic	26 (7%)	13 (7%)	13 (7%)
Dilated	12 (3%)	6 (3%)	6 (3%)
Arrhythmogenic	10 (3%)	2 (1%)	8 (4%)
Hypertrophic	8 (2%)	5 (3%)	3 (2%)
CHA_2_DS_2_‐VASc score	2.1 ± 1.4	2.3 ± 1.4	2.0 ± 1.4
LA diameter (mm)^h^	45.0 ± 7.0	44.6 ± 6.3	45.7 ± 7.8
LA surface area (cm^2^)^i^	26.3 ± 6.3	26.1 ± 6.2	26.6 ± 6.4
AF type			
Persistent AF	303 (82%)	147 (79%)	156 (85%)
Long-standing persistent AF	67 (18%)	40 (21%)	27 (15%)
AF history (years)	2.6 ± 3.5	2.7 ± 3.7	2.4 ± 3.3
AF max sustained duration (months)	8.1 ± 7.2	8.4 ± 7.0	7.8 ± 7.4
AF max sustained duration category			
<6 months	165 (45%)	76 (41%)	89 (49%)
≥6 months	205 (55%)	111 (59%)	94 (51%)
Baseline medications			
Amiodarone	130 (35%)	64 (34%)	66 (36%)
Other Class I or III AADs	19 (5%)	11 (6%)	8 (4%)
Beta blockers	228 (62%)	114 (61%)	114 (62%)

^a^Values presented are mean ± s.d. or *n* (%). Continuous variables were analyzed by two-sided Student’s *t*-test or Wilcoxon test. Two-sided Fisher’s exact test was used for the categorical data.

^b^Data were available for 187 patients (tailored) and 182 patients (anatomical).

^c^Data were available for 186 patients (tailored) and 183 patients (anatomical).

^d^Data were available for 186 patients (tailored) and 179 patients (anatomical).

^e^Data were available for 187 patients (tailored) and 181 patients (anatomical).

^f^Data were available for 186 patients (tailored) and 182 patients (anatomical).

^g^Data were available for 147 patients (tailored) and 140 patients (anatomical).

^h^Data were available for 80 patients (tailored) and 70 patients (anatomical).

^i^Data were available for 114 patients (tailored) and 118 patients (anatomical).

TIA, transient ischemic attack; NYHA, New York Heart Association; AADs, antiarrhythmic drugs.

After enrollment, 7 patients did not undergo ablation, and 13 dropped out before the end of the blanking period, which was the first 3 months after the initial ablation (withdrawal of consent). These patients were not included in the outcome analyses. Details on dropouts and premature exits are provided in Fig. 1. Adherence to study follow-up visits was 98.2% (95% confidence interval (CI), 97.2–98.9); adherence to Holter monitoring at each visit was 91.0% (95% CI, 89.2–92.7); and adherence to electrocardiogram (ECG) monitor transmission, occurring at least weekly, during the 12-month follow-up period was 83.6% (95% CI, 83.1–84.2), per patient on average. There were no significant differences between groups (Extended Data Table 1).

The per-protocol (PP) population analysis included 269 patients (137 and 132 patients in the tailored and anatomical arms, respectively) in whom no major study protocol deviation was present (Extended Data Table 2). The most common reason for exclusion from the PP analysis was insufficient rhythm monitoring (25/43 and 21/45 in the tailored and anatomical arms, respectively). In addition, the overall number of major deviations was similar between the groups, and the numbers of deviations within each category between the two arms were similar. Importantly, there were no statistical differences between the characteristics of the resulting groups of the PP population (Extended Data Table 3).

### Primary efficacy endpoint

**Fig. 2:**
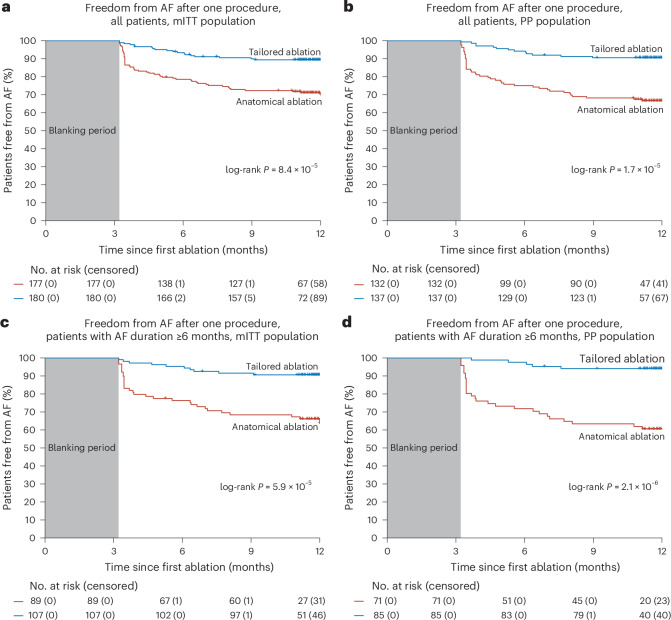
Kaplan–Meier analysis of the primary endpoint. **a**–**d**, Kaplan–Meier estimates of freedom from the primary endpoint, which is freedom from documented AF more than 30 s after a single procedure, with or without the use of antiarrhythmic medications after the 3-month blanking period. Comparisons of the tailored arm versus the anatomical arm were performed using the two-sided log-rank test. **a**, All patients in the mITT population (HR, 0.34; 95% CI, 0.21–0.57). **b**, All patients in the PP population (HR, 0.29; 95% CI, 0.16–0.51). **c**, Patients with persistent AF with a duration of AF of 6 months or more in the mITT population (HR, 0.29; 95% CI, 0.15–0.55). **d**, Patients with persistent AF with a duration of AF greater or equal to 6 months in the PP population (HR, 0.18; 95% CI, 0.08–0.40). In **a**–**d**, *P* < 0.0001 for all comparisons between the tailored and anatomical arms.

Among patients who underwent an ablation (*n* = 370), the primary efficacy endpoint (freedom from AF) was achieved in 158 who underwent a tailored ablation and was not achieved in 22; 7 had no follow-up data beyond the blanking period. In the anatomical arm, the primary endpoint was achieved in 124 patients and was not achieved in 53; 6 had no follow-up data beyond the blanking period. The estimated probability of treatment success in the modified-intention-to-treat (mITT) population (*n* = 357) was 88% for the tailored procedure and 70% for the anatomical procedure (hazard ratio (HR), 0.3; 95% CI, 0.21–0.57; log-rank *P* < 0.0001) (Fig. 2a). The difference between the two arms was confirmed in the PP population (*n* = 269; Fig. 2b) and in the 306 patients (32 versus 19 in the tailored and anatomical arms, respectively; *P* = 0.07) that were not taking antiarrhythmic drugs at 12 months (*n* = 306; 86% versus 68%; HR, 0.35; 95% CI, 0.21–0.59; log-rank *P* < 0.001).

In the prespecified subgroup of patients with an AF duration of ≥6 months in the mITT population (*n* = 196), the difference in treatment success was even more substantial, with a 23% difference between the two arms (Fig. 2c). This effect was mainly due to a less favorable outcome in the anatomical arm (65%), whereas the tailored arm stayed at 88%. A difference of up to 30% was achieved between arms in the subgroup of patients with an AF duration of ≥6 months in the PP population (*n* = 156; Fig. 2d).

### Secondary efficacy endpoints

**Fig. 3:**
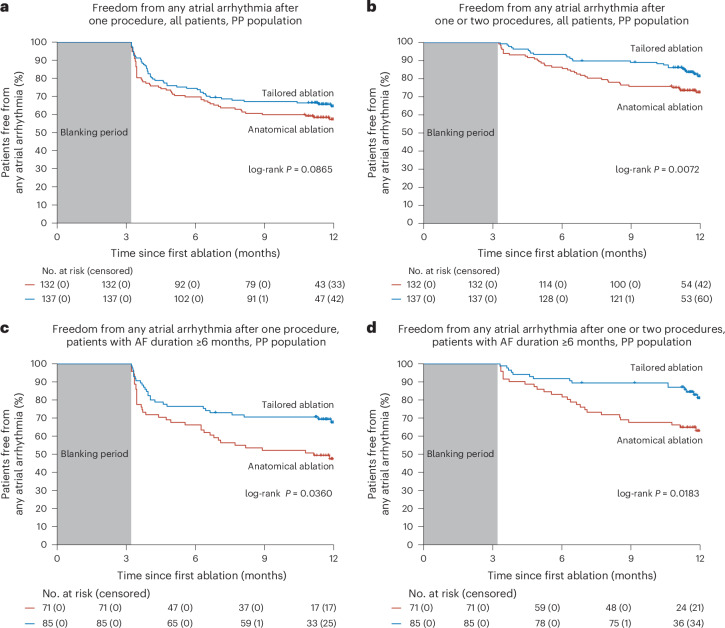
Kaplan–Meier analysis of secondary effectiveness endpoints. **a**–**d**, Kaplan–Meier estimates of freedom from the secondary endpoints, which are freedom from any documented atrial arrhythmia more than 30 s after a single procedure, or up to two procedures, with or without the use of antiarrhythmic medications after a 3-month blanking period. Comparisons of the tailored arm versus anatomical arm were performed using the two-sided log-rank test. **a**, Outcome after a single procedure for all patients in the PP population (HR, 0.81; 95% CI, 0.56–1.19). **b**, Outcome after one or two procedures for all patients in the PP population (HR, 0.62; 95% CI, 0.38–1.01). **c**, Outcome after a single procedure for patients with a duration of AF greater or equal to 6 months in the PP population (HR, 0.60; 95% CI, 0.37–0.97). **d**, Outcome after one or two procedures for patients with a duration of AF greater or equal to 6 months in the PP population (HR, 0.50; 95% CI, 0.28–0.90).

Regarding secondary endpoints, in the mITT population, more patients in the tailored arm achieved freedom from any atrial arrhythmia at 12 months after one or two ablation procedures (76% versus 71%; HR, 0.77; 95% CI, 0.51–1.16; log-rank *P* = 0.07), whereas there was no difference after one procedure (60% versus 60%; HR, 0.94; 95% CI, 0.68–1.31; log-rank *P* = 0.16). In the PP population, a significantly higher percentage of patients in the tailored arm had freedom from any atrial arrhythmia after 12 months and one or two ablation procedures (Fig. 3b), but the difference after one procedure did not reach significance (Fig. 3a).

The differences between the arms was even more pronounced in the prespecified superior-to-6-month subgroup. A significant difference was observed in favor of the tailored arm both after a single procedure and after one or two procedures (Fig. 3c,d).

### Index procedure characteristics

**Extended Data Fig. 3:**
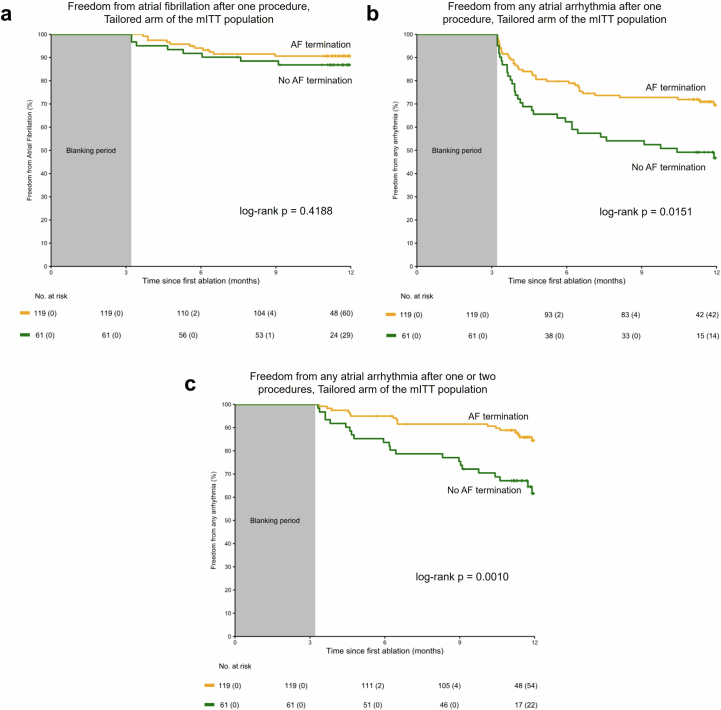
Effect of AF termination in the Tailored arm on the effectiveness endpoints. Kaplan–Meier analysis of the effect of AF termination in the Tailored arm on primary and secondary endpoints. Comparison of patients in the Tailored arm with AF termination by ablation during the index procedure versus patients in the Tailored arm without AF termination was performed using the two-sided log-rank test. **a**, Freedom from AF after a single procedure, HR 1.37 (0.59 – 3.22). **b**, Freedom from any atrial arrhythmia after one procedure, HR 2.03 (1.28 – 3.23). **c**, Freedom from any atrial arrhythmia after one or two procedures, HR 2.75 (1.52 – 4.99).

The characteristics of the index procedures are presented in Table 2. The periprocedural AF termination rate was significantly higher in the tailored arm than in the anatomical arm (66% versus 15%; *P* < 0.001). In the tailored arm, AF terminated by ablation directly to sinus rhythm in 27 patients (22%) and to regular atrial tachycardias (ATs) in 95 patients (78%). In these 95 patients, an average of 1.5 ATs were targeted for further ablation during the index procedure. Although AF termination during the index procedure in the tailored arm had no impact on the rate of AF recurrence at 12 months, it significantly improved the rate of freedom from any atrial arrhythmia (Extended Data Fig. 3).

**Table 2:** Procedural characteristics

Characteristic^a^	Tailored (*n* = 187)	Anatomical (*n* = 183)	*P* value^b^
Procedure time (min)	178 ± 60	92 ± 36	<2.2 × 10^–16^
Initial mapping time (3D navigation ± AI mapping) (min)	31 ± 22	10 ± 5	<2.2 × 10^–16^
Additional mapping time (for example, for AT) (min)	17 ± 15	NA	NA
Fluoroscopy time (min)	9 ± 10	5 ± 4	7.1 × 10^–9^
Total RF time (min)	42 ± 17	20 ± 11	<2.2 × 10^–16^
Location of dispersion			
LA anterior wall	126/150 (84%)	NA	NA
LA posterior wall	129/150 (86%)	NA	NA
LA roof	141/150 (94%)	NA	NA
LA lower septum	104/150 (69%)	NA	NA
LA floor posterior	103/150 (69%)	NA	NA
LA base LAA	78/150 (52%)	NA	NA
Mitral isthmus	71/150 (47%)	NA	NA
RA anterior	58/157 (37%)	NA	NA
Proximal CS	57/157 (36%)	NA	NA
RA upper septum	46/157 (29%)	NA	NA
Crista terminalis	55/157 (35%)	NA	NA
LA ablation extent (%)	31 ± 14	17 ± 8	<2.2 × 10^–16^
Acute AF termination by ablation	122/186 (66%)	26/169 (15%)	<2.2 × 10^–16^
Acute sinus rhythm conversion by ablation	100/187 (53%)	23/172 (13%)	3.0 × 10^–16^

^a^Values presented are mean ± s.d. or *n* (%).

^b^Continuous variables were analyzed by two-sided Student’s *t*-test or Wilcoxon test. Two-sided Fisher’s exact test was used for the categorical data.

CS, coronary sinus; LAA, left atrial appendage; NA, not applicable; RA, right atrium.

The average total procedure time as well as the total radiofrequency (RF) delivery time was significantly longer in the tailored arm than in the anatomical arm (178 ± 60 min versus 92 ± 36 min for procedure duration and 42 ± 17 min versus 20 ± 11 min for RF duration, respectively; *P* < 0.001).

**Extended Data Fig. 4:**
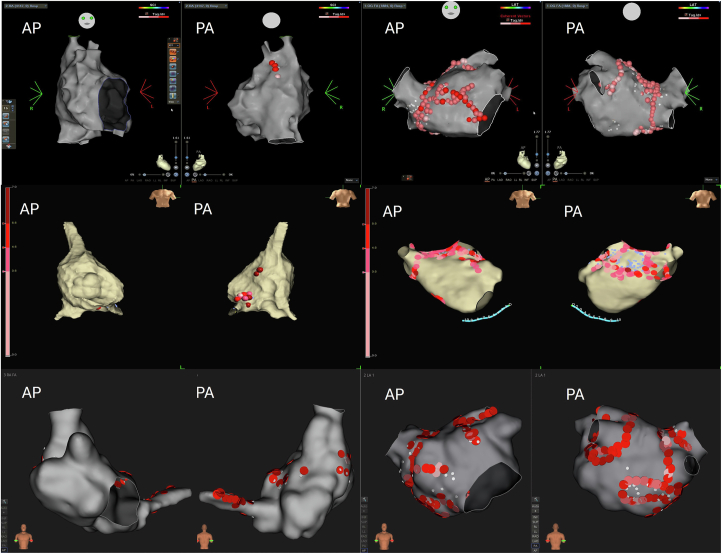
Examples of electroanatomic maps showing the AI-guided dispersion and final ablation sets in the Tailored arm. Different maps are presented in antero-posterior (AP) and postero-anterior (PA) views of right and left atria (left and right panels, respectively). Clusters of white and blue dots correspond to AI-detected dispersion regions. Red tags correspond to radiofrequency ablation tags. On the left atrial map presented in the top right panel, the posterior wall region was not completely ablated because of the proximity with the esophagus.

Examples of electroanatomic maps including the AI-based dispersion tags and corresponding ablation sets in the tailored arm are provided in Extended Data Fig. 4.

### Repeat procedures

During the study period, a total of 88 patients (50/180 versus 38/177 in the tailored and anatomical arms, respectively; *P* = 0.18) underwent a repeat catheter ablation procedure. Of these, 3 repeat procedures in the tailored arm and 4 procedures in the anatomical arm were performed during the last 3 months of follow-up and were outside of the analysis window. The number of repeat procedures considered in the multiple-procedures endpoint was not significantly different between the arms (47/180 versus 34/177; *P* = 0.13). At the repeat procedure, there was no significant difference in terms of PVI durability between the arms: 50% and 42% of patients (*P* = 0.51) had all 4 PVs still isolated and PVI durability was 73% and 71% per vein (*P* = 0.72) in the tailored and anatomical arms, respectively.

In the anatomical arm, repeat procedures included a roof line and mitral line (*n* = 13; 36%), posterior wall isolation (*n* = 9; 25%), posterior wall isolation and mitral line (*n* = 3; 8%), roof line (*n* = 4; 11%), or mitral line (*n* = 1; 3%), averaging 1.6 anatomical lines per patient. No information was provided for 2 patients, and the remaining 6 patients (17%) just had PV re-isolation and/or other ATs mapped and ablated.

In the tailored arm, of the 50 repeat procedures, 43 (86%) were performed for ATs. An average of 1.3 ATs per patient were ablated, most of them (59%) being common macro-reentries including perimitral flutters (47%), roof-dependent flutters (20%), peritricuspid flutters (18%), and a minority being other flutters (15%). Termination by ablation was achieved in all 43 AT procedures (100%).

By contrast, in the anatomical arm, 29 of the 38 repeat procedures (76%) were performed for AF.

### Safety endpoint

Death, cerebrovascular events, or major treatment-related serious adverse events (composite safety endpoint) occurred in 8/187 (4%) patients who underwent a tailored cardiac ablation procedure and 5/183 (3%) patients who underwent PVI only (*P* > 0.05 for all comparisons; Table 3). Two patients in the tailored arm died, one because of Guillain–Barré syndrome and the other from sarcoma, at 5 and 7 months after the ablation, respectively, without having experienced arrhythmia recurrence. Both deaths were classified as unrelated to the ablation procedure or cardiac disease. No strokes or atrio-esophageal fistulas occurred. Cardiac tamponade, pacemaker implantation, and severe cardiac decompensation occurred in equal numbers in both arms (2, 1, and 1 events, respectively). In the anatomical arm, one patient had a puncture-site hemorrhage requiring transfusion, and another an aspiration pneumonia. One patient in the tailored arm experienced transient phrenic nerve palsy.

**Table 3:** Safety endpoint and procedural adverse events

Event^a^	Tailored (*n* = 187)	Anatomical (*n* = 183)	*P* value^b^
Subjects with major procedure-related complications	5	5^c^	>0.99
Cardiac tamponade or perforation	2	2	>0.99
Bradycardia with pacemaker implantation	1	1	>0.99
Severe cardiac decompensation	1	1	>0.99
Puncture-site hemorrhage requiring transfusion	0	1	>0.99
Transient phrenic nerve palsy	1	0	>0.99
Pneumonia aspiration	0	1	>0.99
Subjects with minor procedure-related complications	15^d^	6^d^	0.07
Fluid overload^e^	9	3	0.14
Pericarditis or pericardial effusion^f^	4	0	0.12
Vascular access complication	3	3	>0.99
Post-ablation fever	1	0	>0.99
Abnormal ECG requiring hospitalization	0	1	>0.99
Subjects with non-procedure-related adverse events	3	0	0.25
Death^g^	2	0	0.50
Transient ischemic attack	1	0	>0.99

^a^The primary safety endpoint was a composite of death, cerebrovascular events, or treatment-related serious adverse events. Serious adverse events are reported for patients who underwent an ablation procedure, regardless of whether they completed at least 3 months of follow-up. There were no significant differences between groups.

^b^Two-sided Fisher’s exact test was used for the categorical data.

^c^One patient who had a severe cardiac decompensation was subsequently implanted with a pacemaker; accordingly, the individual components add to more than the total number of patients with any event.

^d^Two patients had vascular access complications and fluid overload events; one patient who had a mild pericardial effusion subsequently had a fluid overload event; accordingly, the individual components add to more than the total number of patients with any event.

^e^Patients were treated with diuretics only and had favorable outcomes.

^f^Patients were treated with corticosteroids or colchicine, and the events resolved without drainage or surgical intervention.

^g^Two patients died as a result of Guillain–Barré syndrome and sarcoma, 5 and 7 months after the ablation, respectively, without arrhythmia recurrence; thus, both deaths were classified as unrelated to the ablation procedure or cardiac disease.

## Discussion

The TAILORED-AF trial is the first randomized superiority trial including patients with persistent AF, comparing tailored, AI-guided ablation in addition to PVI with a standard-of-care PVI-only procedure. The study shows that a single tailored cardiac ablation targeting AI-detected spatio-temporal dispersion areas alongside PVI is superior to PVI only in eliminating AF at 1-year follow-up in patients with persistent and long-standing persistent AF. Patients in the tailored arm also experienced a higher rate of freedom from any arrhythmia after a mean of 1.26 ablation procedures (47 out of 180 patients required a second procedure). Importantly, the most pronounced benefit of this individualized, AI-guided approach was present in patients with longer AF duration and more advanced atrial remodeling, in whom PVI alone showed only poor to moderate success rates.

Previous consensus guidelines^3,4^ have highlighted the high recurrence rates of AF following catheter ablation using only PVI in patients with persistent and long-standing persistent AF^13–16^. Despite this, ablation strategies yielding superior results over the standard PVI procedure are yet to be identified. Strategies that have been evaluated so far include PVI plus anatomical procedures that apply a one-size-fits-all approach (for example, PVI plus predefined left atrial ablation line^6^, PVI plus left atrial appendage isolation^17^, PVI plus posterior left atrial wall ablation^9^, or PVI plus alcohol injection in the vein of Marshall plus linear lesions^7^). These additional lesions are not adjusted to the individual AF-maintaining substrate. Importantly, most randomized clinical trials implementing these non-individualized extensive ablation strategies beyond PVI have failed to achieve improved success rates^6,9^.

Therefore, efforts have been made to develop more personalized adjunctive ablation strategies. These include ablation targeting areas of atrial fibrosis or low voltage^8,18,19^, or targeting presumed electrical AF drivers such as complex fractionated electrograms, rotors, high dominant frequency sites, or non-pulmonary foci^20–25^. Although some smaller studies have reported promising results in single-center trials, these personalized strategies targeting electrical AF drivers have not demonstrated superiority in large-scale trials. This is likely owing to the fact that the detection and adjudication of the electrical AF substrate has not yet reached a sufficient level of objectivity, reliability, and especially reproducibility.

This is where AI can step in. AI is progressively being used in transformative technologies across various sectors, including healthcare. Its ability to leverage large-scale medical data to generate adjudications that surpass human capabilities has resulted in highly successful applications, particularly those in which complex pattern recognition is key, such as diagnostic radiology^26^. However, the potential advantages of AI-driven software for directly steering therapy are largely untapped^27,28^.

In brief, the AI-driven software used in this trial identified complex intricate clusters of electrograms and categorized them as spatio-temporal dispersion. This led to an objective, reproducible, and reliable identification of ablation target areas for individual patients across all 26 centers and 51 operators. The results presented herein indicate that this individualized, AI-driven, electrogram-guided ablation played a crucial role in eliminating AF and achieving superiority over the standard PVI-only procedure.

Procedures in the tailored arm required additional procedural steps and were associated with a significantly longer procedure and ablation time (Table 2 and Extended Data Fig. 2) compared with PVI-only ablation. Procedure duration, as well as the duration of RF delivery in the tailored arm, were in accordance with other approaches with PVI and proved as safe as the PVI-only approach^6,9^. Future developments in AI training, procedural workflow, and ablation modalities could lead to improvements in efficacy and precision. For example, larger-footprint catheters and new ablative energies, such as pulsed field ablation, might allow for more effective and efficient procedures^29,30^. Furthermore, an extended interoperability with existing mapping technologies could offer automation of manual procedural steps.

Interestingly, on the basis of prespecified subgroup analyses, a tailored procedure seems to be particularly beneficial in patients with prolonged persistent AF (≥6 months). These patients often have advanced atrial remodeling with more AF-maintaining substrate located beyond the pulmonary veins. Our results suggest that AI-guided detection and ablation of extra-PV AF-maintaining substrate areas are key to reaching arrhythmia elimination in many patients.

Our findings indicate that the arrhythmia recurrence type was vastly different between the groups. In the anatomical arm, AF was the prevailing arrhythmia, whereas the majority of recurrences in the tailored arm were regular (reentrant) ATs more readily ablated by investigators, as evidenced by the increased number of repeat procedures. These ATs might be regarded as a ‘simplification’ of AF^31^ into an organized reentry, and thus into an arrhythmia that is mechanistically better understood and can be treated successfully by catheter ablation. In line with this, previous reports have indicated that the occurrence of ATs is associated with a significantly better outcome than AF recurrence, suggesting that AT might be considered as an important step towards sinus rhythm restoration^10,32,33^.

Notably, in the tailored arm, AF termination during the index procedure did not have any measurable impact on the rate of freedom from AF at 12 months. By contrast, it was associated with a significantly higher rate of freedom from any atrial arrhythmia at 12 months, aligning with previous investigations^10,12,20,34^.

Our trial has several limitations: (1) the primary endpoint focuses only on freedom from AF, and does not account for freedom from any atrial arrhythmia after one procedure. In this complex population with persistent AF, AT recurrences, which are generally easier to ablate, can be seen as a step towards stable sinus rhythm. (2) RF 4-mm ablation-tip catheters are not the ideal ablation tools for such a workflow. New ablation tools might decrease the need for AT repeat ablations and reduce the procedure time. (3) The 12-month follow-up duration is adequate relative to previously conducted large randomized trials on cardiac catheter ablation^7–9,29,30^. Still, a longer follow-up period might have had the advantage of presenting a more accurate account of the number of patients that are arrhythmia-free after several procedures. (4) To consistently evaluate the benefit of procedures using the AI software solution presented above, its machine-learning model had to be frozen in 2020.

In summary, this is the first large-scale international randomized trial of catheter ablation in a persistent AF population to show the benefit of a procedure consisting of ablation beyond PVI guided by intracardiac electrograms. The use of AI, in particular machine learning, for the objective, reproducible, and reliable identification of ablation target areas seems to have been pivotal in achieving these results and provides hope for further applications of AI in interventional medicine.

## Methods

### Trial design

The Tailored Versus Anatomical Ablation Procedure for Persistent Atrial Fibrillation Trial (TAILORED-AF, NCT04702451) was a prospective, interventional, multicenter, randomized, controlled, double-blind (study subject and endpoint assessor) study comparing the effectiveness of two ablation procedures: a tailored cardiac ablation procedure targeting areas harboring spatio-temporal dispersion^12^ detected by AI in addition to PVI versus an anatomical PVI-only procedure. The trial was funded by the manufacturer of the AI adjudication system, Volta Medical. The trial received Investigational Device Exemption by the Food and Drug Administration (since the use of the AI adjudication system to guide ablation is investigational in the United States) and was approved by the institutional or ethics review board at each center: Western IRB (WIRB), Ascension St. Vincent IRB, Rhode Island Hospital IRB (United States), CPP SUD-EST IV (France), Technische Universität München (TUM) Ethikkommission, Landesärztekammer Baden-Württemberg Ethikkommission (Germany), OLV Ziekenhuis vzw Ethisch Comite (Belgium), and Brabant Medical Ethics Committee (Netherlands). It was conducted in accordance with principles of the Declaration of Helsinki. The trial was designed by the sponsor with input from an international steering committee (Supplementary Table 2) led by the first author. A Data Safety Monitoring Board, composed of three independent electrophysiologists, reviewed safety events and monitored the study conduct. A blinded independent ECG core lab was responsible for rhythm monitoring. Data monitoring, collection, and primary data analysis were performed by an independent contract research organization. The first draft of this manuscript was written by the first author, with subsequent reviews and edits by the other authors. Although the sponsor contributed suggestions, the final decision on the content of the manuscript rested with the first author. The authors vouch for the accuracy and completeness of the data and for the adherence of the trial to the protocol. The detailed protocol is available in the Supplementary Note 1. The statistical analysis plan is available in the Supplementary Note 2.

### Study participants

After providing written informed consent, adults with symptomatic persistent or long-standing persistent AF (sustained duration longer than or equal to 3 months, but not longer than 5 years) refractory to at least one antiarrhythmic drug and who were candidates for first-time ablation were enrolled in Europe and in the United States. In the United States, all patients with long-standing persistent AF (>1 year) were excluded from participation to avoid off-label ablation catheter use, because nothing was approved for such use at the time. Patients with severe obesity, a very dilated left atria, previous atrioventricular valve surgery, or chronic severe heart failure were excluded. Detailed inclusion and exclusion criteria are provided in Extended Data Table 4. All participants received anticoagulants for a minimum of 4 weeks before their ablation procedure. Criteria and procedures for withdrawal or termination of participation are provided in Extended Data Table 5.

### Randomization and masking

Participants were randomly assigned in a 1:1 ratio to either the control anatomical group (PVI-only) or the investigational tailored group undergoing a tailored cardiac ablation procedure targeting AI-identified electrogram spatio-temporal dispersion in addition to PVI (Fig. 1 and Extended Data Fig. 1). Randomization was performed using a random permuted block method through the electronic case report form, with block sizes randomly chosen as two, four, and six. Randomization was stratified according to AF type and site. The randomization outcome was communicated to the operator after patient enrollment, and patients were blinded to the randomization outcome.

### Machine-learning algorithm

The Volta AF-Xplorer (previously VX1, Volta Medical) software is an embedded, expertise-based AI tool trained to detect spatio-temporal dispersion in multipolar intracardiac electrograms^10^. The Volta AF-Xplorer algorithm is an ensemble binary classification model (Supplementary Fig. 2). This model is built on the aggregated output of two distinct binary classification models: a knowledge-based classification model (extreme gradient boosting algorithm) working on a series of extracted features and a deep-learning classification algorithm (convolutional neural network). These models were trained offline on a proprietary database of 275,020 annotated intracardiac electrograms in 2017, using a proprietary digital platform enabling data collection and signal annotation. Electrograms were digitized and processed in real-time (inference time less than 100 ms) by Volta AF-Xplorer, which provided operators with visual and audio cues representing areas of interest. A direct connection from the acquisition system to a computer installed with the Volta AF-Xplorer software enabled data transmission.

The algorithm’s overall performances were evaluated using a testing dataset of 113,920 single-annotated electrograms and a dataset of 14,490 electrograms annotated by five expert cardiac electrophysiologists from France and the United States. The board-certified cardiac electrophysiologists independently annotated electrograms measured by multipolar mapping catheters with virtually unlimited time for analysis to assess the presence or absence of dispersion. Each physician was unaware of the others’ annotations. The aim of this study was to evaluate whether the agreement levels between cardiac electrophysiologists correlated well with the algorithm’s analysis.

### Procedures

The tailored cardiac ablation procedure consisted of distinct steps using Volta AF-Xplorer, which included: identification of regions of interest assisted by AI, and annotation of these regions on bi-atrial three-dimensional (3D) navigation maps using three commercially available navigation systems (Carto3, Biosense Webster; EnSite Precision or EnSite X, Abbott Medical; and Rhythmia HDx, Boston Scientific; Extended Data Table 6). The procedural goal was to perform targeted ablation in regions highlighted by the Volta AF-Xplorer system, followed by the completion of PVI (Extended Data Figs. 1 and 2), aiming at AF termination. Operators were asked to connect ablated areas to an electrically neutral structure (isolated pulmonary veins or valvular annuli), or to other ablated areas if they were within 2 cm of each other. If an ablation line was created during the procedure, validation was required only in the case of documented macro-reentry. If AF terminated into a regular (reentry) AT, operators were asked to map and ablate these arrhythmias and any subsequent ones, aiming to achieve intra-procedural restoration of sinus rhythm using only catheter ablation. If AF or AT persisted, external electrical ablation was recommended. Ablation was performed with the use of RF energy.

In the anatomical arm, operators used a commercially available 3D mapping system, a multielectrode mapping catheter, and a contact force-sensing ablation catheter (Extended Data Table 6). The ablation protocol required a wide-area circumferential PVI, with documented acute bidirectional block being a procedural endpoint. If documented typical right atrial flutter was documented either before or during the procedure, cavo-tricuspid isthmus linear ablation was required. No additional linear ablation was permitted during the index procedure.

### Follow-up

The use of antiarrhythmic medications was allowed during the first 3 months after the initial ablation (the post-ablation blanking period), after which their use was discouraged. Patients with recurrent AF after the blanking period were allowed to start or resume the use of antiarrhythmic medications; they were also given the option to undergo repeat ablation using the same procedure to which they were initially randomly assigned. In the anatomical arm, patients could undergo re-isolation of the pulmonary veins along with up to two additional lines of ablation. If a repeat ablation was deemed appropriate, we recommended that it be performed 3–9 months after the initial procedure. Anticoagulation therapy was continued after ablation for a minimum of 2 months, on the basis of a patient’s CHA_2_DS_2_-VASc score. This score was calculated by summing the points for congestive heart failure (1 point), hypertension (1 point), age ≥75 years (2 points), diabetes (1 point), prior stroke (2 points), vascular disease (1 point), age 65–74 years (1 point), and female sex (1 point).

All participants in both arms were followed for 12 months, with scheduled in-office visits at 3, 6, and 12 months. Clinical assessments, 12-lead ECGs, and 24-hour Holter monitoring recordings were obtained at each visit. Alternatively, home visits by a trained nurse could be performed in France. All patients received a 6-lead Kardia portable monitor and were asked to perform and transmit 30-s rhythm recordings at least weekly and whenever they had symptoms during the complete duration of the study. ECGs, Holter-monitor recordings, and transmissions from the portable monitor were reviewed independently by the members of the ECG Core Lab’s Medical Adjudication Committee, who were unaware of the treatment assignments.

### Populations

The safety population included all randomized participants who underwent their first ablation procedure.

The mITT set included all randomized participants except for those who were deemed ineligible after randomization and before the ablation procedure, those who did not have any ablation procedure, and those lost to follow-up during the 3-month blanking period.

The prespecified PP analysis set consisted of participants in whom the study took place in line with the PP defined milestones. Specifically, these were participants who met all the inclusion criteria and none of the exclusion criteria; were treated with one or two ablation procedures, in line with the protocol, during which there were no important procedure-related protocol deviations; and completed the follow-up as mandated, that is did not miss more than one mandatory Holter recording and had a Kardia compliance rate of ≥50%.

### Endpoints

The primary efficacy endpoint was freedom from any documented episode of AF lasting longer than 30 s after the 3-month blanking period and occurring after a single ablation procedure, with or without the use of antiarrhythmic drugs.

The secondary efficacy endpoints were freedom from any documented atrial arrhythmia (including AF, flutter, or tachycardia) after one, or after one or two ablation procedures, with or without the use of antiarrhythmic drugs.

The secondary safety endpoint was a composite of death; cerebrovascular events, that is stroke or transient ischemic attack; and serious treatment-related adverse events, that is adverse events with seriousness criteria and related to the device or the ablation strategy, as assessed by the principal investigator.

A prespecified subgroup analysis on freedom from arrhythmia in participants with persistent AF lasting 6 months or more was conducted, using the same methodology as the primary analysis, on both the mITT and PP populations.

### Data collection and storage

Clinical data were collected through secure electronic case report forms using the iMednet electronic data capture system versions 1.204.0 to 1.241.4, hosted by Mednet Solutions. Follow-up ECG data were centralized and analyzed through a secure web portal, Atrium version 9.1.0, hosted by Banook Group.

### Statistical analysis

Sample-size estimations were based on the assumptions that 77% of the participants in the tailored group would be free from AF 12 months after a single ablation procedure^10,12^, versus 62% in the anatomical group^6^, corresponding to a HR of 0.547. Using a log-rank test for superiority of the tailored group versus the anatomical group and a randomization ratio of 1:1, a total of 292 participants were needed for the study to have a power of 80% at a one-sided alpha level of 0.025. Assuming a dropout rate of 22% (no index ablation performed or loss to follow-up), 374 participants were required.

Assumptions regarding the rates of AF in both study arms were verified after 50% of the originally planned total patient sample (73 in each arm) completed primary endpoint data collection. A conditional power approach for sample-size re-estimation, using the Mehta–Pocock method, was applied. Following this interim assessment, the sample size could only remain the same or be increased.

All statistical analyses were performed on enrolled participants who underwent an ablation procedure and were not lost to follow-up during the 3-month blanking period (the mITT population). Primary and secondary endpoints were analyzed using a Cox proportional-hazards model, with treatment group and a dichotomous effect for AF duration (<6 months or ≥6 months) as independent terms, and right-censoring of participants lost to follow-up. For each endpoint, the alternative hypothesis *S*1(*t*) > *S*2(*t*) was tested against the null hypothesis *S*1(*t*) = *S*2(*t*), where *S*1(*t*) and *S*2(*t*) represent the survival functions at time *t* for the tailored and anatomical arms, respectively. A two-sided log-rank test at a significance alpha level of 0.05 was used, and Kaplan–Meier curves were generated.

Sensitivity analyses of primary and secondary endpoints were performed on the PP population described in Extended Data Table 2.

The composite safety endpoint was tested for non-inferiority in the tailored arm versus the anatomical arm at a 2.5% margin using Fisher’s exact test on proportion.

Other survival endpoints, without formal hypothesis testing, are summarized with descriptive statistics, HRs, corresponding 95% CIs, and log-rank *P* values. The widths of CIs and *P* values have not been adjusted for multiplicity.

For other analyses in this manuscript, the continuous variables were analyzed by a Student’s *t*-test or, if normality assumptions were violated (assessed by Shapiro tests), an equivalent non-parametric method, the Wilcoxon–Mann–Whitney test. Fisher’s exact test was used for the categorical data.

Data were analyzed with SAS Software, version 9.4 (SAS Institute), and R Statistical Software, version 4.3.2 (R Core Team).

### Reporting summary

Further information on research design is available in the Nature Portfolio Reporting Summary linked to this article.

## Online content

Any methods, additional references, Nature Portfolio reporting summaries, source data, extended data, supplementary information, acknowledgements, peer review information; details of author contributions and competing interests; and statements of data and code availability are available at 10.1038/s41591-025-03517-w.

## Supplementary information

Supplementary Information Supplementary Figs. 1 and 2, Tables 1 and 2, and Notes 1 and 2.

Reporting Summary

## Figures and Tables

**Extended Data Table 1:**
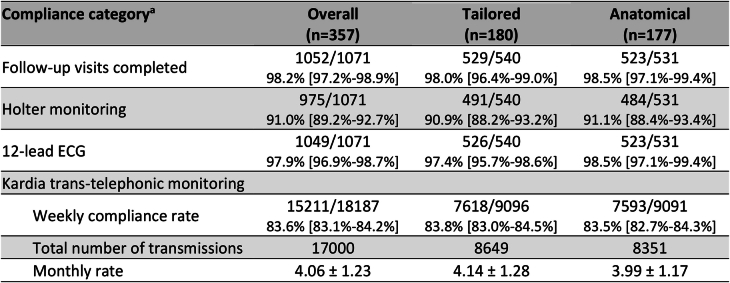
Compliance rates

^a^Values presented are mean ± SD or n (% [95% CI]). There were no significant differences between groups (P < 0.05 was considered to indicate statistical significance, tested by two-sided Student’s t-tests for numerical data and by two-sided exact Fisher’s tests for counts).

**Extended Data Table 2:**
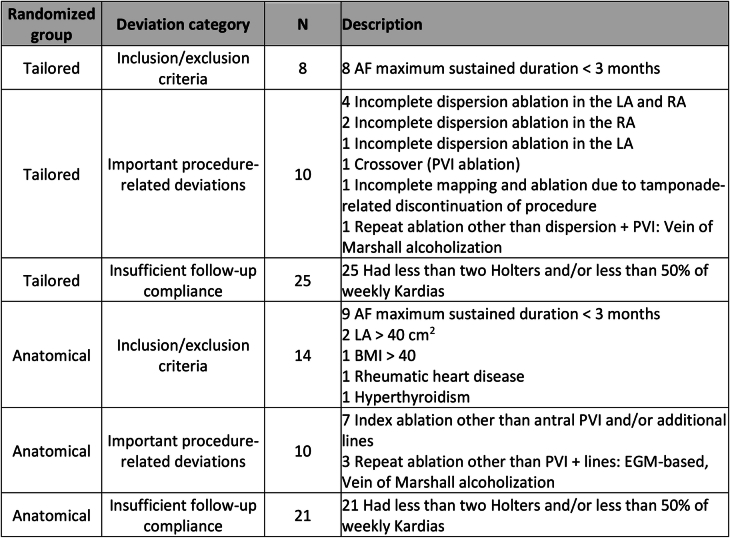
Details of mITT patients not included in the PP population

AF, Atrial Fibrillation; LA, Left Atrium; RA, Right Atrium; PVI, Pulmonary Vein Isolation; BMI, Body Mass Index; EGM, Electrograms.

**Extended Data Table 3:**
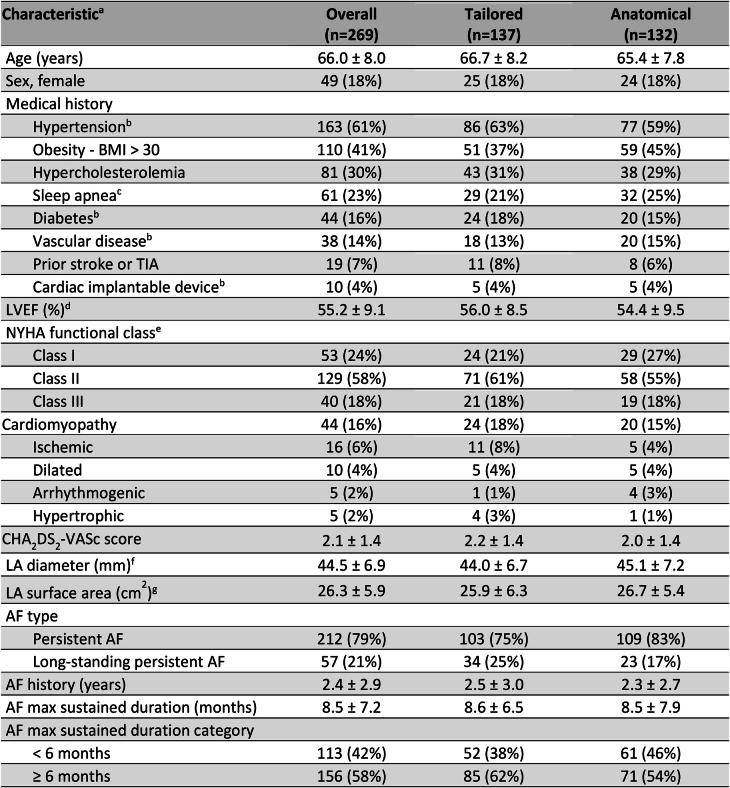
Baseline patient characteristics in the per-protocol population

^a^Values presented are mean ± SD or n (%). Continuous variables were analyzed by two-sided Student’s t-test or Wilcoxon test. Two-sided Fisher’s exact test was used for the categorical data.

^b^Data were available for 137 patients (Tailored) and 131 patients (Anatomical).

^c^Data were available for 136 patients (Tailored) and 128 patients (Anatomical).

^d^Data were available for 136 patients (Tailored) and 132 patients (Anatomical).

^e^Data were available for 116 patients (Tailored) and 106 patients (Anatomical).

^f^Data were available for 52 patients (Tailored) and 48 patients (Anatomical).

^g^Data were available for 89 patients (Tailored) and 90 patients (Anatomical). BMI, body-mass index; TIA, transient ischemic attack; LVEF, left ventricular ejection fraction; NYHA, New York Heart Association; AF, atrial fibrillation; AADs, anti-arrhythmic drugs. CHA_2_DS_2_-VASc scores (an assessment of the risk of stroke among patients with atrial fibrillation) range from 0 to 9, with higher scores indicating a higher risk of stroke.

**Extended Data Table 4:**
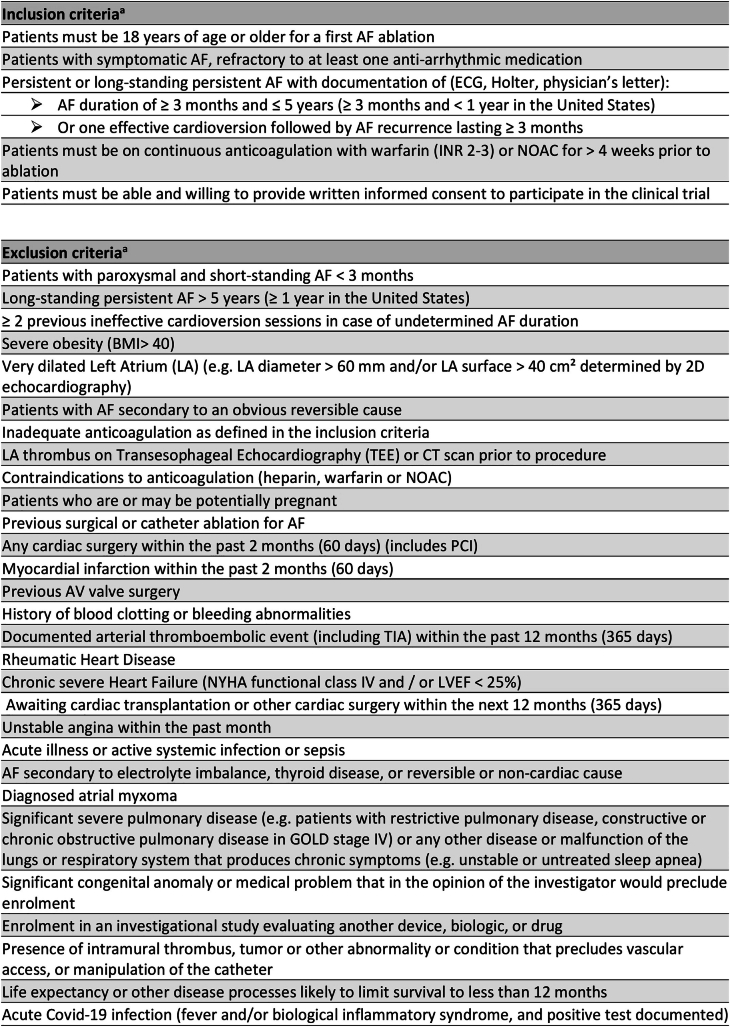
Major inclusion and exclusion criteria

AF, Atrial Fibrillation; ECG, Electrocardiogram; INR, International Normalized Ratio; NOAC, Novel Oral Anticoagulant; BMI, Body Mass Index; PCI, Percutaneous Coronary Intervention; AV, Atrioventricular; TIA, Transient Ischemic Attack; NYHA, New York Heart Association (NYHA) Functional Classification.

^a^For a full list of inclusion and exclusion criteria, refer to the full protocol.

**Extended Data Table 5:**
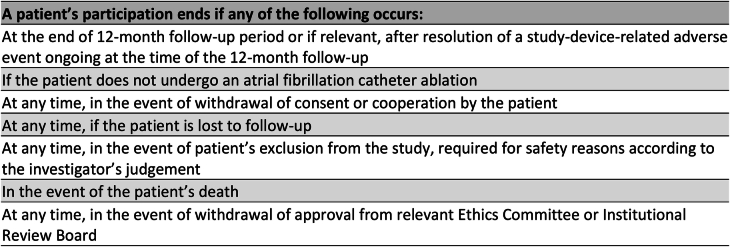
Criteria and procedures for withdrawal or termination of a subject’s participation

**Extended Data Table 6:**
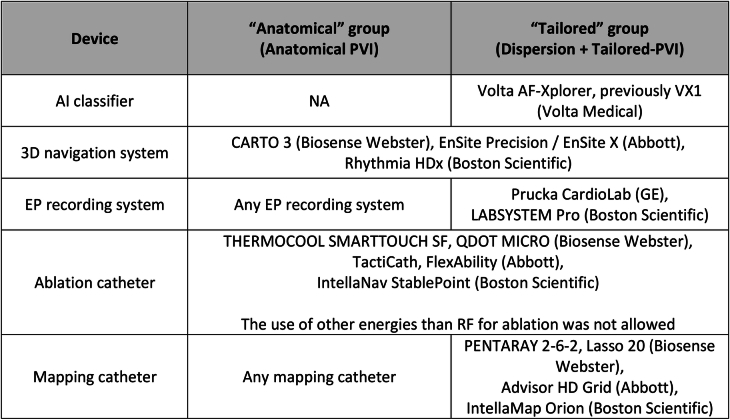
Devices used during the study

In Europe: Only CE-marked devices, locally approved and commercially available, were used in this study. In the United States: Only FDA cleared/approved and commercially available devices, were used in this study.

## Data Availability

All supporting data are available in the article and the Supplementary Information. Source data will not be shared owing to patient privacy obligations applicable to the sponsor under the European Union General Data Protection Regulation (GDPR) and in particular owing to the obligations of privacy to which the sponsor committed itself in the informed consent form signed by the patients.

## Code availability

No custom code was used for analysis of the numerical data reported in this manuscript. The AI system used is a deterministic (locked) medical device commercially available in the European Union and the United States. The AI-based code for EGM adjudication that forms the core of the medical device is intellectual property of Volta Medical and is not publicly available.
