# Supplementary material for: Artificial intelligence for individualized treatment of persistent atrial fibrillation: a randomized controlled trial
Source: Nat Med. 2025 Feb 14;31(4):1286–93. doi: 10.1038/s41591-025-03517-w (PMC12003177; doi:10.1038/s41591-025-03517-w)
Supplement: Supplementary file 1 — Supplementary Figs. 1 and 2, Tables 1 and 2, and Notes 1 and 2. [file 41591_2025_3517_MOESM1_ESM.pdf]

nature medicine

Supplementary information https://doi.org/10.1038/s41591-025-03517-w

Artificial intelligence for individualized treatment of persistent atrial fibrillation: a randomized controlled trial

In the format provided by the authors and unedited

Table of Contents

**Supplementary Table 1**	**Tailored-AF Consortium Investigators**
**Supplementary Table 2**	**Tailored-AF Committees**
**Supplementary Figure 1**	**AI algorithm performance and bench testing**
**Supplementary Figure 2**	**Flowchart diagram explaining the main steps carried out when the trained machine learning model is used on new data**
**Supplementary Note 1**	**Tailored-AF Clinical Investigation Plan**
**Supplementary Note 2**	**Tailored-AF Statistical Analysis Plan**

Supplementary Table 1: Tailored-AF Consortium Investigators.

Site Name and Address	Principal Investigator*	Sub-Investigators
Cardiovascular Center, Onze Lieve Vrouwziekenhuis Hospital<br>Moorselbaan 164<br>9300, Aalst, Belgium	Tom De Potter, MD	(none)
Department of Cardiology, University Hospitals Leuven<br>Herestraat 49<br>3000, Leuven, Belgium	Joris Ector, MD	Peter Haemers, MD
Clinique Pasteur<br>45 Av. de Lombez BP 27617 – 31076<br>31300 Toulouse, France	Jean-Paul Albenque, MD	Stéphane Combes, MD
Clinique Rhena<br>10 Rue François Epailly<br>67000 Strasbourg, France	Babe Bakouboula, MD	Mathieu Schaaf, MD
Hôpital cardiologique Louis Pradel<br>Institut cardiologique de Lyon<br>Hospices Civils de Lyon<br>59 Bd Pinel<br>69500 Bron, France	Francis Bessiere, MD, PhD	Kevin Gardey, MD
Département de Cardiologie, CHU de Nancy<br>5 Rue du Morvan<br>54500 Vandœuvre-lès-Nancy, France	Christian De Chillou, MD	Jean-Marc Sellal, MD<br>Isabelle Magnin, MD
Hopital Saint Joseph<br>26 Bd de Louvain<br>13008 Marseille, France	Edouard Gitenay, MD	Anis Ayari, MD<br>Michel Bremondy, MD<br>Elisa Martinez, MD
Hôpital Privé Jacques Cartier<br>6 Av. du Noyer Lambert<br>91300 Massy, France	Jerome Horvilleur, MD	Jerome Lacotte, MD<br>Mina Ait Said, MD<br>Fiorella Salerno, MD<br>Salem Younsi, MD
Centre Cardiologique du Nord<br>32 - 36 Rue des Moulins Gémeaux<br>93200 Saint-Denis, France	Antoine Lepillier, MD	(none)
Pôle Santé République<br>105 Avenue de la République<br>63050 Clermont-Ferrand, France	Antoine Roux, MD	Marc Mielczarek, MD

Clinique Saint George<br>2 Av. de Rimiez<br>06105 Nice, France	Guillaume Theodore, MD	(none)
Hôpital Saint Philibert<br>Rue du Grand But BP 249<br>59462 Cedex Lomme, France	Yves Guyomar, MD	(none)
Hôpital privé Le Confluent<br>4 Rue Eric Tabarly<br>44000 Nantes, France	Selim Abbey, MD	Nelly Amara, MD<br>Nicolas Malliet, MD
German Heart Center Munich<br>Lazarettstraße 36<br>80636 Munich, Germany	Isabel Deisenhofer, MD	Felix Bourier, MD<br>Sarah Lengauer, MD<br>Tilko Reents, MD<br>Fabian Bahlke, MD<br>Nico Erhard, MD<br>Florian Englert, MD<br>Miruna Popa, MD<br>Hannah Krafft, MD<br>Monika Hofmann, MD<br>Milan Paul, MD<br>Mark Kottmaier, MD<br>Marta Telishevska, MD<br>Gabriele Hessling, MD
Klinik für Kardiologie, Klinikum Coburg<br>Ketschendorfer Str. 33<br>96450 Coburg, Germany	Sonia Busch, MD<br>Mathias Forkmann, MD	Beatriz Tose Costa Paiva, MD<br>Issamedine Ajmi, MD<br>Thomas Mischke, MD<br>Tayfun Acil, MD
Medizinische Klinik IV, Städtisches Klinikum Karlsruhe Moltkestraße 90<br>76133 Karlsruhe, Germany	Matthias Merkel, MD	Patrick Hörmann, MD
Department of Cardiology, Isala Hospital<br>Dokter van Heesweg 2<br>8025 AB Zwolle, The Netherlands	Jaap Jan Smit, MD	(none)
Department of Cardiology, OLVG<br>Jan Tooropstraat 164<br>1061 AE Amsterdam, The Netherlands	Muchtiar Khan, MD	Gijsbert De Ruiter, MD<br>Emilio Schade, MD<br>Anchee Boersma, MD<br>Amaya Hagen, MD
New York Presbyterian Queens<br>Department of Internal Medicine<br>56-45 Main St.<br>Flushing, NY 11355, USA	Seth Goldbarg, MD	Joon Kim, MD

Washington University School of Medicine - Center for Heart Rhythm Disorders<br>660 S. Euclid Ave<br>St. Louis, MO 63110, USA	Daniel Cooper, MD	Phillip Cuculich, MD<br>Timothy Smith, MD<br>Sandeep Sodhi, MD
Inova Fairfax Hospital<br>3300 Gallows Rd<br>Falls Church, VA 22042, USA	Haroon Rashid, MD	Stephen Gaeta, MD
The Ohio State University Wexner Medical Center 473<br>W. 12th Ave Suite 155<br>Colombus, OH 43210, USA	John Hummel, MD	Emile Daoud, MD<br>Mahmoud Houmsse, MD<br>Toshimasa Okabe, MD
Northwell Health Lenox Hill Hospital<br>100 East 77th St<br>New York, NY 10075, USA	Stavros Mountantonakis, MD	Haisam Ismail, MD<br>Nicholas Skipitaris, MD
Ascension St. Vincent's Riverside Hospital<br>1 Shircliff Way<br>Jacksonville, FL 32204, USA	Saumil Oza, MD	(none)
Rhode Island Hospital<br>950 Warren Ave Suite 201<br>East Providence, RI 02914, USA	Daniel Philbin, MD	Estelle Torbey, MD
Alabama Cardiovascular Group<br>3680 Grandview Parkway | Suite 200<br>Birmingham, AL 35243, USA	Gustavo Morales, MD	Anil Rajendra, MD

*Multiple principal investigators are listed if an investigator left an institution during the trial and another investigator at that site became the principal investigator.

Supplementary Table 2: Tailored-AF Committees.

**Members of the Steering Committee**
Isabel Deisenhofer, MD (Chair, Coordinating Investigator)
Jean Paul Albenque, MD
Sonia Busch, MD
Christian De Chillou, MD
Tom De Potter, MD
Seth Goldbarg, MD
John Hummel, MD
Atul Verma, MD

**Members of the Data Safety Monitoring Board**
Etienne Aliot, MD (Chair)
Paul-Ursmar Milliez, MD
Xavier Waintraub, MD

**Members of the ECG core lab**
Nicolas Sadoul, MD (Chair, Kardia/ECG Adjudication Committee)
Laurence Guedon-Moreau, MD (Back-up Chair, Kardia/ECG Adjudication Committee)
Olivier Huttin, MD (Chair, Holter Adjudication Committee)
Claude Kouakam, MD
Hugues Blangy, MD
Jean-Pierre Rebmann, MD
Stephane Cosson, MD
Arnaud Olivier, MD
Aicha Fofana, MD
Florian Baptiste, MD

Supplementary Figure 1: AI algorithm performance and bench testing.

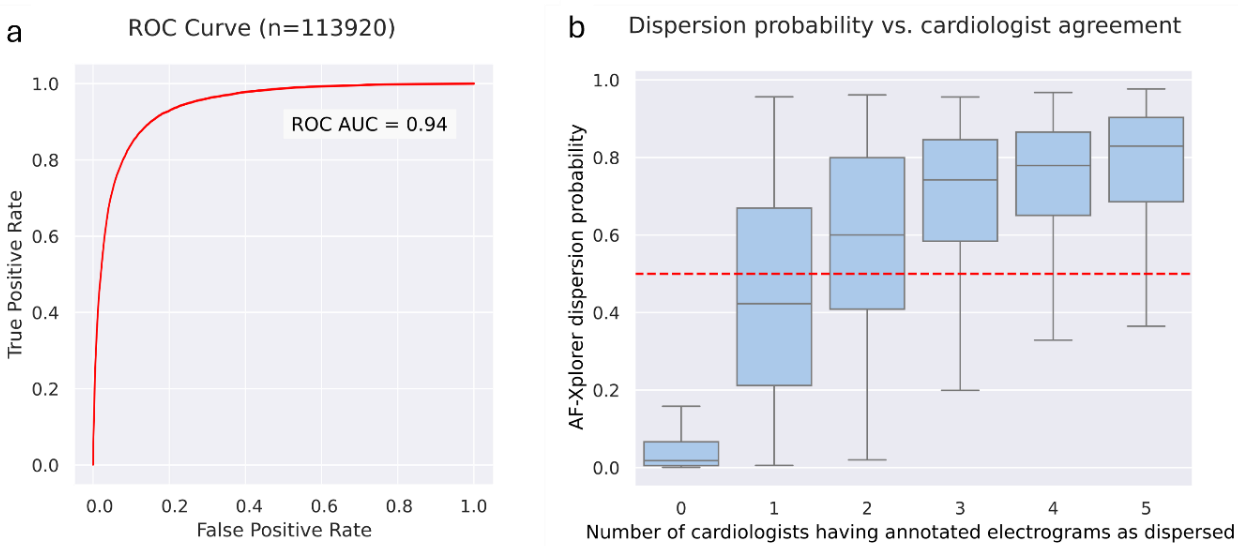

a, ROC diagram for 'dispersed' versus 'non-dispersed' adjudicated electrograms. The red ROC curve is created by sweeping a threshold over the predicted probability of dispersion. The true positive rate (i.e., "hit rate"), and the false positive rate (i.e., "false alarm rate"), measure performance. **b**, Relationship between output probability and cardiologist agreement levels. For electrograms adjudicated as 'dispersed' by respectively zero, one, two, three, four or five cardiologists (x-axis), box-plots illustrate the distribution of the output probability of the algorithm (y-axis).

Supplementary Figure 2: Flowchart diagram explaining the main steps carried out when the trained machine learning model is used on new data.

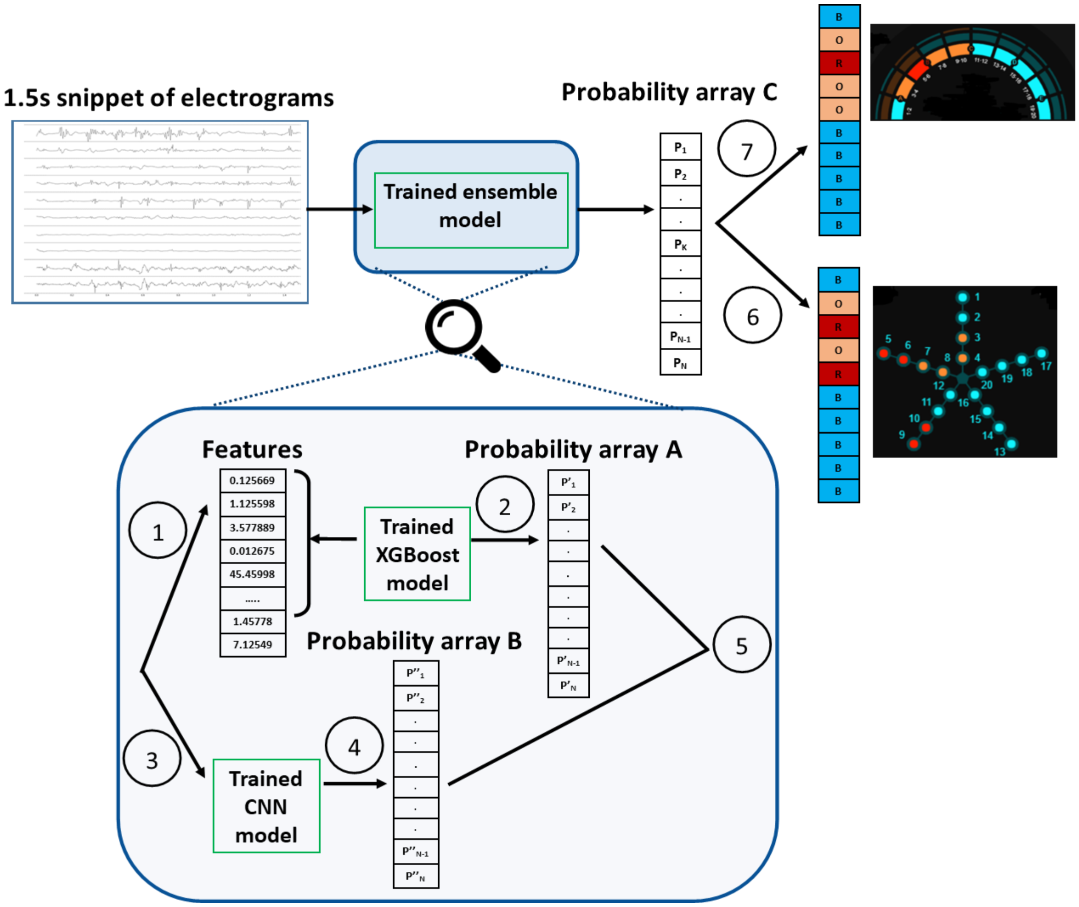

Electrogram information provided by the electrophysiology recording system is processed by a feature extraction module extracting 65 features per single track (Step 1), these features are analyzed by a trained distributed gradient boosting machine learning algorithm to produce an array of dispersion probability A (Step 2). In parallel, it is processed by a trained convolutional neural network algorithm (Step 3) producing an array of dispersion probability B (Step 4). Both dispersion likelihood arrays are merged using a weighted average based on their agreement level to produce dispersion probability C. Weights have been chosen using a 5-fold cross-validation (Step 5). Array C is color-coded and displayed on the catheter schematic (Step 6). To account for time-wise stability, several iterations in time are used to build a different color-coded list which is displayed on the upper frame of the software interface (Step 7).

The Tailored-AF trial

CLINICAL INVESTIGATION PLAN

CONFIDENTIAL

**TITLE OF THE STUDY**	**Tailored vs Anatomical Ablation Strategy for Persistent AF**
**CIP No.**	CLIPL-01-002
**TYPE OF STUDY**	Interventional, prospective, randomized, controlled, two-arm, multicenter clinical investigation
**SPONSOR**	**Volta Medical**<br>65 Avenue Jules Cantini<br>13006 Marseille, FRANCE
**COORDINATING INVESTIGATOR**	**Prof. Isabel DEISENHOFER**<br>German Heart Centre Munich<br>Lazarettstraße 36<br>80636 Munich, GERMANY
**CONTRACT RESEARCH ORGANIZATION**	**LABCORP**<br>Medical Device and Diagnostic Solutions

Table of contents

ABBREVIATIONS 5

SYNOPSIS OF THE STUDY 7

I – RATIONALE 11

II – DIAGRAM OF THE STUDY 13

III – DESCRIPTION OF STUDY DEVICES 14

3.1 – Devices used in this investigation 14

3.2 – Other devices 14

IV – ENDPOINTS OF THE CLINICAL INVESTIGATION 15

4.1 – Primary endpoint 15

4.2 – Secondary endpoints 15

4.3 – Other exploratory endpoints 16

V – DESIGN OF THE STUDY 17

5.1 – General 17

5.1.1 – Description of the type of clinical investigation 17

5.1.2 – Description of measures to be taken to minimize or avoid bias 17

5.2 – Subjects and study period 17

5.2.1 – Inclusion criteria 17

5.2.2 – Exclusion criteria 18

5.2.3 – Criteria and procedures for withdrawal or termination of a subject's participation 19

5.2.4 – Expected total duration of the clinical investigation 19

5.2.5 – Expected duration of participation for each subject 20

5.2.6 – Number of subjects required to be included in the clinical investigation 20

5.2.7 – Estimated time required to include this number (i.e. the period of enrollment) 20

5.3 – Study procedures 20

5.3.1 – Screening and enrollment 20

5.3.2 – Randomization 21

5.3.3 – Pre-ablation Medication 21

5.3.4 – Index procedure 21

5.3.5 – Trans Telephonic event Monitor (TTM) 28

5.3.6 – Follow-up period 28

5.3.7 – Redo procedure 29

5.3.8 – Blanking periods 29

5.3.9 – Visit schedule 29

5.3.10 – Summary of required clinical assessment and data collection 30

5.4 – Investigators and sites 31

This document is confidential and proprietary of Volta Medical. All copy is considered uncontrolled. User must verify the revision of uncontrolled copy before use.

CONFIDENTIAL

5.5 – Steering Committee 31
5.6 – Monitoring plan 31
VI – STATISTICAL CONSIDERATIONS 32
6.1 – Design, statistical methodology and analytical procedures 32
6.2 – Sample size 33
6.3 – Degree of significance and power of the clinical investigation 33
6.4 – Expected drop-out rate 33
6.5 – Evaluation criteria for primary and secondary endpoints 33
6.5.1 – Long-term effectiveness primary endpoint 33
6.5.2 – Other secondary and exploratory endpoints 34
6.6 – Interim analysis 34
6.7 – Missing data and sensitivity analysis 34
6.8 – Minimum and maximum number of subjects to be included for each site 35
6.9 – Poolability of Sites 35
VII – RISKS DESCRIPTION AND MINIMIZATION 35
7.1 – Expected clinical benefits 35
7.2 – Expected adverse effects of the device 35
7.3 – Risks associated with participation in the clinical investigation 35
7.4 – Measures to control or mitigate risks 36
7.5 – Justification of the risk-benefit ratio 36
VIII – DATA MANAGEMENT 36
IX – AMENDMENTS TO THE CLINICAL INVESTIGATION PLAN 37
X – DEVIATIONS FROM CLINICAL INVESTIGATION PLAN 37
10.1 – General 37
10.2 – Procedures for recording, reporting and analyzing deviations from the Clinical Investigation Plan 38
10.3 – Corrective and preventive actions and criteria for disqualification of the Principal Investigator 38
XI – DEVICE ACCOUNTABILITY 38
XII – STATEMENTS OF COMPLIANCE 39
12.1 – Ethical and regulatory considerations 39
12.2 – Insurance 39
XIII – INFORMED CONSENT PROCESS 39
XIV – ADVERSE EVENTS, ADVERSE DEVICE EFFECTS AND DEVICE DEFICIENCIES 39
14.1 – Definitions (according to ISO 14155:2020) 39
14.2 – Adverse event evaluation and reporting 41

This document is confidential and proprietary of Volta Medical. All copy is considered uncontrolled. User must verify the revision of uncontrolled copy before use.

CONFIDENTIAL

14.3 – Anticipated adverse events 42
14.4 – Data Safety Monitoring Board (DSMB) 42
XV – SUSPENSION OR PREMATURE TERMINATION OF THE CLINICAL INVESTIGATION 43
15.1 – Criteria and provisions 43
15.2 – Patient follow-up requirements 43
XVI – STUDY REPORT AND PUBLICATION POLICY 43
XVII – BIBLIOGRAPHY 44
XVIII – APPENDICES 47
18.1 – Declaration of Helsinki 47
18.2 – Insurance Certificate 47
18.3 – CE Certificates 47
18.4 – Patient Information and Patient Informed Consent 47
18.5 – Investigator Agreement 47
18.6 – IFU or Investigator’s Brochure 47
18.7 – List of Investigators 47
18.8 – Study Boards 47

CONFIDENTIAL

ABBREVIATIONS

AAD	Antiarrhythmic Drug
AE	Adverse Event
AF	Atrial Fibrillation
ANSM	French National Agency for Medicines and Health Products Safety
AT	Atrial Tachycardia
AV	Atrio-Ventricular
BMI	Body Mass Index
CFAE	Complex Fractionated Atrial Electrograms
CIP	Clinical Investigation Plan
CNIL	French National Commission for Data Protection and Liberties
CPP	Committee for the Protection of Persons – French Ethics Committee
CRA	Clinical Research Associate
CRO	Contract Research Organization
CS	Coronary Sinus
CTI	Cavo-Tricuspid Isthmus
DC	Direct-Current
DSMB	Data Safety Monitoring Board
EC	Ethics Committee
ECG	Electrocardiogram
eCRF	Electronic Case Report Form
EGM	Intracardiac Electrogram
EP	Electrophysiology/Electrophysiologist
FU	Follow-up
GCP	Good Clinical Practices
IB	Investigator's Brochure
IRB	Institutional Review Board
LA	Left Atrial/Atrium
LVEF	Left Ventricular Ejection Fraction
NOAC	Non-vitamin K antagonist oral anticoagulants
NYHA	New York Heart Association
PCI	Percutaneous Coronary Intervention

CONFIDENTIAL

PV	Pulmonary Vein
PVI	Pulmonary Veins Isolation
QoL	Quality-of-Life
RA	Right Atrial/Atrium
RAA	Right Atrial Appendage
RF	Radiofrequency
SR	Sinus Rhythm
TEE	Transesophageal Echocardiography
TIA	Transient Ischemic Attack
TTM	Trans Telephonic event Monitor

CONFIDENTIAL

SYNOPSIS OF THE STUDY

Title of the Study:	Tailored vs Anatomical Ablation Strategy for Persistent AF
CIP No.:	CLIPL-01-002
Design of the Study:	Interventional, prospective, randomized, controlled, two-arm, multicenter clinical investigation
Sponsor:	Volta Medical<br>65 Avenue Jules Cantini<br>13006 Marseille, FRANCE
Coordinating Investigator:	Prof. Isabel DEISENHOFER<br>German Heart Centre Munich<br>Lazarettstraße 36<br>80636 Munich, GERMANY
Contract Research Organization:	LABCORP, Medical Device and Diagnostic Solutions
Justification / Context:	Atrial Fibrillation (AF) ablation is typically performed in predefined anatomic regions of the left atrium without attempting to identify patient-specific areas of interest. This procedure is referred to as Pulmonary Vein Isolation (PVI).<br>The hypothesis in this Study is that a tailored ablation strategy targeting areas of spatio-temporal dispersion in combination with PVI is superior to an anatomical ablation strategy targeting PVI alone for the treatment of persistent AF.
Primary endpoint:	1. Freedom from documented AF, with or without antiarrhythmic drugs (AADs), 12 months after a single index ablation procedure.
Secondary endpoints:	2. Freedom from documented AF and Atrial Tachycardia (AT), after one or two procedures, with or without AADs, at 12 months<br>3. Freedom from documented AF/AT, after one procedure, with or without AADs, at 12 months<br>4. Safety composite endpoint at 12 months: death, cerebrovascular events, or serious treatment-related adverse event
Exploratory endpoints:	5. Freedom from documented AF, after one or two procedures, with or without AADs, at 12 months<br>6. Freedom from documented AF or AF/AT, after one procedure, or one procedure or more, without AADs, at 12 months<br>7. Freedom from documented AF or AF/AT, after one procedure, or one procedure or more, without AADs or with previously failed AAD at the same dose, at 12 months<br>8. Freedom from documented AF or AF/AT, after one procedure, or one procedure or more, with or without AADs, at 12 months, without the use of TTM<br>9. Freedom from documented symptomatic AF or AF/AT, after one procedure, or one procedure or more, with or without AADs, at 12 months

CONFIDENTIAL

	10. Freedom from AF or AF/AT, with or without AADs, 12 months after one procedure, or one procedure or more, for subjects with persistent AF <1 year<br>11. Freedom from AF or AF/AT, with or without AADs, 12 months after one procedure, or one procedure or more, for subjects with AF ≥ 6 months (including long-standing persistent AF)<br>12. Estimated AF or AF/AT burden, with or without AADs, after one procedure, or one procedure or more<br>13. Quality of life measurements (SF-36 and AFEQT) at baseline, 3, 6 and 12 months post-index procedure<br>14. Incidence of peri-procedural complications including stroke, PV stenosis, cardiac perforation, esophageal injury, and death<br>15. Acute AF termination rate by ablation<br>16. Rate of conversion to sinus rhythm by ablation<br>17. Ablation procedure duration<br>18. Fluoroscopy time and dose<br>19. Mapping time<br>20. Radiofrequency (RF) time to terminate AF<br>21. Total RF time<br>22. Total delivered energy (mean power x RF duration)<br>23. Total delivered energy (mean power x RF duration) / biatrial surface<br>24. Ablation surface area to terminate AF<br>25. Total ablation surface area<br>26. Blinded baseline Voltage maps before ablation<br>27. TTM compliance (% of transmissions expected vs received at each week post index procedure)<br>28. Assessment of blind maintenance per group
Inclusion criteria:	1. Patients 18 years of age or older candidates for a first AF ablation<br>2. Symptomatic AF, refractory to at least one antiarrhythmic medication<br>3. Persistent or long-standing persistent AF with <u>documentation of </u>(ECG, Holter, physician's letter):<br>• AF duration of ≥ 3 months and ≤ 5 years (≥ 3 months and < 1 year in the United States)<br>or<br>• 1 effective cardioversion followed by AF recurrence lasting ≥ 3 months<br>4. Continuous anticoagulation with warfarin (INR 2-3) or NOAC for > 4 weeks prior to ablation<br>5. Patients must be able and willing to provide written informed consent to participate in the clinical trial<br><br>At least 60% of patients (224 patients) in persistent AF ≥ 6 months including at least 15% (56 patients) of long-standing persistent AF ≥ 12 months

CONFIDENTIAL

Exclusion criteria:	1. Paroxysmal and short-standing AF < 3 months<br>2. Long-standing persistent AF > 5 years (≥ 1 year in the United States)<br>3. ≥ 2 previous ineffective cardioversion sessions in case of undetermined AF duration<br>4. Severe obesity (BMI > 40)<br>5. Very dilated Left Atrium (LA) (e.g. LA diameter > 60mm and/or LA surface > 40 cm^2 determined by 2D echocardiography)<br>6. Patients with AF secondary to an obvious reversible cause<br>7. Inadequate anticoagulation as defined in the inclusion criteria<br>8. LA thrombus on Transesophageal Echocardiography (TEE)* or CT Scan prior to procedure<br>9. Contraindications to anticoagulation (heparin, warfarin or NOAC)<br>10. Patients who are or may potentially be pregnant<br>11. Previous surgical or catheter ablation for AF<br>12. Any cardiac surgery within the past 2 months (60 days) (includes PCI)<br>13. Myocardial infarction within the past 2 months (60 days)<br>14. Previous AV valve surgery<br>15. History of blood clotting or bleeding abnormalities<br>16. Documented arterial thromboembolic event (including TIA) within the past 12 months (365 days)<br>17. Rheumatic Heart Disease<br>18. Chronic severe Heart Failure (NYHA functional class IV and/or LVEF < 25%)<br>19. Awaiting cardiac transplantation or other cardiac surgery within the next 12 months (365 days)<br>20. Unstable angina within the past month<br>21. Acute illness or active systemic infection or sepsis<br>22. AF secondary to electrolyte imbalance, thyroid disease, or reversible or non-cardiac cause<br>23. Diagnosed atrial myxoma<br>24. Significant severe pulmonary disease, (e.g. patients with restrictive pulmonary disease, constrictive or chronic obstructive pulmonary disease in GOLD stage IV) or any other disease or malfunction of the lungs or respiratory system that produces chronic symptoms (e.g. unstable or untreated sleep apnea)<br>25. Significant congenital anomaly or medical problem that in the opinion of the investigator would preclude enrollment<br>26. Enrollment in an investigational study evaluating another device, biologic, or drug<br>27. Presence of intramural thrombus, tumor or other abnormality or condition that precludes vascular access, or manipulation of the catheter<br>28. Life expectancy or other disease processes likely to limit survival to less than 12 months<br>29. Acute Covid-19 infection (fever and/or biological inflammatory syndrome, and positive test documented)

CONFIDENTIAL

	* Only TEE is acceptable in the United States to screen for LA thrombus. If a subject cannot have a TEE performed, then intracardiac echocardiography at the beginning of the procedure to screen for LA thrombus is acceptable.
Number of patients to be included:	Approximately 374 patients (187 in each group)<br>(Up to 80 patients in the US)
Estimated duration of the Study:	- Study set-up: 4 months<br>- Inclusion phase: 22 months<br>- Patients follow-up: 12 months<br>- Database lock, analysis, and close-out: 3 months<br>- Total duration of the Study: 41 months
Duration of patient's participation in the Study:	Up to 16 months (from enrollment to end of follow-up)

CONFIDENTIAL

I – RATIONALE

Introduction

During the past 20 years, atrial fibrillation (AF) has become a major public health problem. AF is the most common arrhythmia in adults affecting about 10 million patients in the European Union alone.[1–3] AF is associated with significant morbidity and mortality, a decreased quality of life and contributes to increasing healthcare costs in Western countries.[4] Still, state-of-the-art AF management remains largely sub-optimal. With current therapeutic approaches, it is estimated that only half of the patients with drug-refractory AF will ever be cured from the disease.[5] One of the main bottlenecks to improving AF management is the difficulty to durably terminate the arrhythmia and prevent its re-occurrence. In recent years, intense research has enabled the development of an interventional approach to curing AF, named as catheter ablation. Ablation consists of introducing catheters within the heart and delivering tissue-modifying energies in selected sub-regions. Ablation has generated a lot of hope in patients and providers, but its long-term success rate (~50%) remains deceptive, especially for patients with persistent AF.[5]

While various ablation strategies have been suggested with heterogeneous results,[5–11] pulmonary vein isolation (PVI) has emerged as the cornerstone approach.[12–14] The clinical efficacy of PVI for controlling AF, however, is limited by arrhythmia recurrences due to PV reconnection and/or non-PV triggers. Thus, PVI may not be sufficient to treat all types of AF patients. Multiple works have suggested that the success rate of an ablation procedure could be improved if a sub-set of intra-cardiac electrograms could be identified and targeted.[15–21] Such approaches, coined as electrogram-based ablation, require sophisticated skills or, alternatively, analytical software to precisely identify the electrograms of interest. Previous research has pointed towards the usefulness of recognizing and targeting multipolar intra-cardiac electrograms exhibiting spatio-temporal dispersion. Specifically, it was demonstrated that the ability to identify dispersed intra-cardiac electrograms (EGMs) could be key to ablation success.[20,21]

Background studies

The Substrate HD study (ClinicalTrials.gov Identifier: NCT02093949) published in the Journal of the American College of Cardiology in 2017 described the visual criteria that may be used to identify intra-cardiac electrograms exhibiting spatio-temporal dispersion.[21] Also, this work presented preliminary findings supporting that the targeted ablation of dispersed electrograms may represent an efficacious approach for patients with AF.

Electrogram-based ablation procedure success relies on operators' experience and skills at deciphering multiple tracks-signals while at the same time manipulating intra-cardiac catheters with millimeter precision. The level of dispersed EGMs complexity is such that several years of experience are required to reliably analyze dispersed EGMs. Multiple innovative solutions have been developed to facilitate EGM analysis during AF ablation. For example, the module CARTO CFAEs provides cartographic information based upon measurement of electrogram fractionation.[22,23] This type of product, however, represents an analysis of individual per-procedural data sets. Thus, this technology "ignores" the thesaurus of analytical parameters that pertain to previous similar procedures. The development of Volta Medical algorithms, relying on artificial intelligence (AI), represents an attempt at satisfying a medical need for an automatic and reliable detection of dispersed electrograms. The methods rely on conceptualizing a discriminant algorithm, which draws anonymized information from a very large database of intra-cardiac EGMS.

The Ev-AIFib study (ClinicalTrials.gov Identifier: NCT03434964) aimed to evaluate the performance and safety of Volta Medical most advanced software VX1 on 300 patients, 8 sites and 18 operators. It was concluded that VX1 is well suited for real-time detection of spatio-temporal dispersion during an AF/AT ablation procedure. Moreover, VX1 allowed for robust center-to-center standardization of acute and long-term ablation outcomes after electrogram-based ablation of persistent AF.[24]

This document is confidential and proprietary of Volta Medical. All copy is considered uncontrolled. User must verify the revision of uncontrolled copy before use.

Objective

In this study, our objective is to demonstrate that a tailored ablation strategy targeting areas exhibiting spatio-temporal dispersion in association with PVI, is superior to an anatomical probabilistic ablation strategy targeting PVI alone for the treatment of persistent AF. Electrograms harboring spatio-temporal dispersion will be identified using VX1.

CONFIDENTIAL

II – DIAGRAM OF THE STUDY

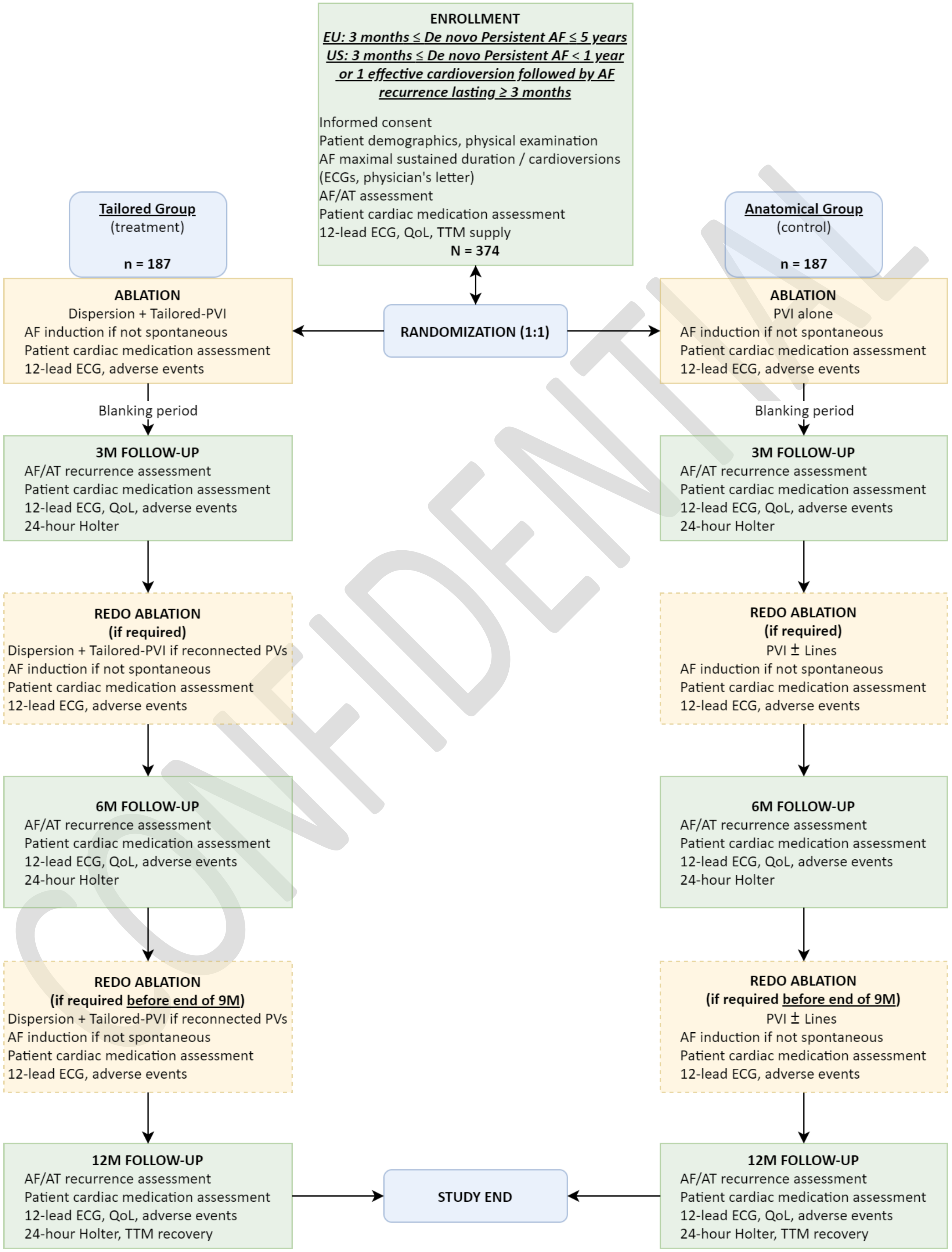

Figure 1: Diagram of the Study

This document is confidential and proprietary of Volta Medical. All copy is considered uncontrolled. User must verify the revision of uncontrolled copy before use.

III – DESCRIPTION OF STUDY DEVICES

3.1 – Devices used in this investigation

The following Volta Medical products are required to be used in the investigation:

- VX1 Application (*ref. S001*), version 1.3 or later.
  VX1 Application is a class IIa stand-alone software. VX1 Application received initial CE mark on 14 JAN 2020.
- VX1 Hardware (*ref. H001*), version 1.0 or later.
  VX1 Hardware is a class I system including a computer with integrated A/D converter with its power supply adapter and detachable power cord, a screen with its power supply adapter and detachable power cord, a data transfer cable, a keyboard and a mouse. VX1 Hardware received CE mark on 09 JUL 2020.

In the United States: A single device, including both software and hardware modules and identified as VX1 (*ref. A001*), is marketed in the USA. VX1 as used in this protocol is investigational in the US.

3.2 – Other devices

Table 1 lists all additional market approved devices that are required to be used in this investigation, and specifies the authorized or compatible brand and manufacturer, if applicable.

Table 1: Authorized other devices

Device	"Anatomical" group (PVI)	"Tailored" group (Dispersion + Tailored-PVI)
3D navigation system	CARTO 3 (Biosense Webster) or EnSite Precision / EnSite X (Abbott) or Rhythmia (Boston Scientific)	
EP recording system	Any EP recording system	Prucka CardioLab (GE) or LABSYSTEM Pro (Boston Scientific)
CS catheter	Any decapolar catheter, regardless of the manufacturer	
Ablation catheter	THERMOCOOL SMARTTOUCH SF (Biosense Webster) or TactiCath (Abbott) or FlexAbility (Abbott) or QDOT MICRO (Biosense Webster) or other RF ablation catheter<br>The use of contact catheter is highly recommended<br>The use of other energies than RF for ablation is not allowed	
Mapping catheter	Any mapping catheter	PENTARAY 2-6-2, Advisor HD Grid, Reflexion HD, IntellaMap Orion

The following market approved devices are allowed to be used in the investigation regardless of the manufacturer:

- Esophageal thermal probe

In Europe: Only CE-marked devices, locally approved and commercially available, will be used within this protocol.

In the United States: Only devices that are FDA cleared/approved and commercially available, will be used. In particular, only the THERMOCOOL SMARTTOUCH SF ablation catheter used with the CARTO 3 navigation system is FDA approved for ablation of persistent AF. Other ablation catheters with compatible navigation systems may be used upon FDA approval for persistent AF indications.

CONFIDENTIAL

IV – ENDPOINTS OF THE CLINICAL INVESTIGATION

4.1 – Primary endpoint

The primary endpoint is defined in Table 2.

Table 2: Primary endpoint

Endpoint	Description
Long-term effectiveness primary endpoint	1. Freedom from documented AF*, with or without AADs, 12 months after a single index ablation procedure.<br><br>**Freedom from documented AF is defined as no documented episodes of AF > 30 seconds with conventional non-invasive monitoring (Holters, TTM and 12-lead ECG recordings) between 3 and 12 months after index procedure*<br>*Difference of freedom from arrhythmia between the arms will be analyzed as comparison of survival distributions of the two arms in order to take into account the time to arrhythmia recurrence and the censored data (cf. 6.5 – Evaluation criteria for primary and secondary endpoints)*

Possible scenarios that may result in primary endpoint failure:

- A repeat procedure where AF is confirmed during the procedure
- A 30-second or greater episode of AF is documented on a 24-hour Holter prior or subsequent to a follow-up visit
- 30-second of AF on a weekly TTM transmission confirmed by 30-second of AF on same or next week TTM transmission
- A 10-second 12-lead ECG recording confirming AF suggested by either TTM or Holter and subject symptoms

4.2 – Secondary endpoints

Secondary endpoints are defined in Table 3 below.

Table 3: Secondary endpoints

Endpoint	Description
Other long-term effectiveness endpoints	2. Freedom from documented AF/AT*, after one or two procedures, with or without AADs, at 12 months<br>3. Freedom from documented AF/AT*, after one procedure, with or without AADs, at 12 months<br><br>**Freedom from documented AF/AT is defined as no documented episodes of any atrial arrhythmia > 30 seconds with conventional non-invasive monitoring (Holters, TTM and 12-lead ECG recordings) between 3 and 12 months after index procedure*<br>*Difference of freedom from arrhythmia between the arms will be analyzed as comparison of survival distributions of the two arms in order to take into account the time to arrhythmia recurrence and the censored data (cf. 6.5 – Evaluation criteria for primary and secondary endpoints)*
Long-term safety endpoint	4. Safety composite endpoint at 12 months: death, cerebrovascular events, or serious treatment-related adverse event

This document is confidential and proprietary of Volta Medical. All copy is considered uncontrolled. User must verify the revision of uncontrolled copy before use.

CONFIDENTIAL

4.3 – Other exploratory endpoints

Exploratory endpoints are defined in Table 4 below.

Table 4: Exploratory endpoints

Endpoint	Description
Other long-term effectiveness endpoints	5. Freedom from documented AF, after one or two procedures, with or without AADs, at 12 months<br>6. Freedom from documented AF or AF/AT, after one procedure, or one procedure or more, without AADs, at 12 months*<br>7. Freedom from documented AF or AF/AT, after one procedure, or one procedure or more, without AADs or with previously failed AAD at the same dose, at 12 months**<br>8. Freedom from documented AF or AF/AT, after one procedure, or one procedure or more, with or without AADs, at 12 months, without the use of TTM†<br>9. Freedom from documented symptomatic AF or AF/AT, after one procedure, or one procedure or more, with or without AADs, at 12 months‡<br>10. Freedom from AF or AF/AT, with or without AADs, 12 months after one procedure, or one procedure or more, for subjects with persistent AF <1 year<br>11. Freedom from AF or AF/AT, with or without AADs, 12 months after one procedure, or one procedure or more, for subjects with AF ≥ 6 months (including long-standing persistent AF)<br>12. Estimated AF or AF/AT burden[#], with or without AADs, after one procedure, or one procedure or more<br><br>*Primary and secondary endpoints 1-4 with no AAD after the blanking period<br>**Primary and secondary endpoints 1-4 with no AAD after the blanking period or with previously failed AAD at the same dose<br>†Primary and secondary endpoints 1-4 with limited non-invasive monitoring (Holters and 12-lead ECG only)<br>‡Primary and secondary endpoints 1-4 considering only documented episodes for which the patient was symptomatic, based on patient declaration (e.g. through TTM recordings annotation) and Quality-Of-Life questionnaire results<br>[#]Post-ablation arrhythmia burden is estimated based on TTM and/or Holter recordings
Quality of Life	13. QoL measurements (SF-36 and AFEQT) at baseline, 3, 6 and 12 months post-index procedure
Acute safety endpoint	14. Incidence of peri-procedural complications including stroke, PV stenosis, cardiac perforation, esophageal injury, and death
Acute effectiveness endpoints	15. AF termination rate by ablation*<br>16. Rate of conversion to sinus rhythm by ablation*<br><br>*Correlation between acute effectiveness and long-term effectiveness will be assessed in both arms
Additional evaluations	17. Ablation procedure duration<br>18. Fluoroscopy time and dose<br>19. Mapping time<br>20. Radiofrequency (RF) time to terminate AF

CONFIDENTIAL

	21. Total RF time<br>22. Total delivered energy (mean power x RF duration)<br>23. Total delivered energy (mean power x RF duration) / biatrial surface<br>24. Ablation surface area to terminate AF<br>25. Total ablation surface area<br>26. Blinded baseline Voltage maps before ablation*<br>27. TTM compliance (% of transmissions expected vs received at each week post index procedure)<br>28. Assessment of blind maintenance per group<br><br>**Voltage maps must be performed blindly by the operator. In the Tailored arm, the baseline Voltage map is performed during dispersion mapping. In the Anatomical arm, the baseline Voltage map is performed during fast-anatomical mapping.*

V – DESIGN OF THE STUDY

5.1 – General

5.1.1 – Description of the type of clinical investigation

This study concerns a medical device post-CE marking, and has the following characteristics:

- Interventional: Enrolled patients will undergo an AF ablation[1]
- Prospective: Patients are enrolled prior to undergoing an AF ablation
- Multi-center: This study will take place in several sites, at least 5 different sites with a maximum number of inclusions per site as defined in this protocol (cf. 6.8 – Minimum and maximum number of subjects to be included for each site). A maximum of 8 sites may participate in the United States.
- Controlled: Patients in the Anatomical arm (conventional procedure) constitute the control group
- Randomized, two-arm: At enrollment, the patient is randomized 1:1 to one of the arms, Anatomical or Tailored
- Simple-blinded: The patient is not aware of the randomization

5.1.2 – Description of measures to be taken to minimize or avoid bias

All patients will be randomized at enrollment in a 1:1 ratio. The patient will not be informed of the randomization.

Special care will be taken to ensure that the patient will be blinded to his/her randomization assignment throughout the study. Patient unblinding should be documented as a non-important deviation.

Moreover, a blind rhythm monitoring is planned: all ECG recordings for long-term effectiveness endpoints (Holter, TTM and 12-lead ECG) will be analyzed and adjudicated blindly by an independent ECG core lab.

5.2 – Subjects and study period

5.2.1 – Inclusion criteria

Patients who meet all the following criteria at the time of enrollment may be included:

1. Patients 18 years of age or older candidates for a first AF ablation

[1] In France, according to the Jardé Law, it is a category 2 interventional study, involving only minimal risks and constraints

This document is confidential and proprietary of Volta Medical. All copy is considered uncontrolled. User must verify the revision of uncontrolled copy before use.

2. Symptomatic AF, refractory to at least one antiarrhythmic medication
3. Persistent or long-standing persistent AF with documentation of (ECG, Holter, physician's letter):
    - AF duration of ≥ 3 months and ≤ 5 years (≥ 3 months and < 1 year in the United States)
      Or
    - 1 effective[2] cardioversion followed by AF recurrence lasting ≥ 3 months
4. Continuous anticoagulation with warfarin (INR 2-3) or NOAC for > 4 weeks prior to ablation
5. Patients must be able and willing to provide written informed consent to participate in the clinical trial

At least 60% of patients (224 patients) in persistent AF ≥ 6 months including at least 15% (56 patients) of long-standing persistent AF ≥ 12 months

5.2.2 – Exclusion criteria

Patients who meet any of the following criteria will be excluded from the investigation:

1. Paroxysmal and short-standing AF < 3 months
2. Long-standing persistent AF > 5 years (≥ 1 year in the United States)
3. ≥ 2 previous ineffective[3] cardioversion sessions in case of undetermined AF duration
4. Severe obesity (BMI > 40)
5. Very dilated Left Atrium (LA) (e.g. LA diameter > 60 mm and/or LA surface > 40 cm^2 determined by 2D echocardiography)
6. Patients with AF secondary to an obvious reversible cause
7. Inadequate anticoagulation as defined in the inclusion criteria
8. LA thrombus on Transesophageal Echocardiography (TEE)* or CT Scan prior to procedure
9. Contraindications to anticoagulation (heparin, warfarin or NOAC)
10. Patients who are or may potentially be pregnant
11. Previous surgical or catheter ablation for AF
12. Any cardiac surgery within the past 2 months (60 days) (includes PCI)
13. Myocardial infarction within the past 2 months (60 days)
14. Previous AV valve surgery
15. History of blood clotting or bleeding abnormalities
16. Documented arterial thromboembolic event (including TIA) within the past 12 months (365 days)
17. Rheumatic Heart Disease
18. Chronic severe Heart Failure (NYHA functional class IV and/or LVEF < 25%)
19. Awaiting cardiac transplantation or other cardiac surgery within the next 12 months (365 days)
20. Unstable angina within the past month
21. Acute illness or active systemic infection or sepsis
22. AF secondary to electrolyte imbalance, thyroid disease, or reversible or non-cardiac cause
23. Diagnosed atrial myxoma
24. Significant severe pulmonary disease (e.g. patients with restrictive pulmonary disease, constrictive or chronic obstructive pulmonary disease in GOLD stage IV) or any other disease or malfunction of the lungs or respiratory system that produces chronic symptoms (e.g. unstable or untreated sleep apnea)

CONFIDENTIAL

[2] An effective cardioversion means that normal sinus rhythm has been restored at the end of the procedure and that the last ECG before discharge shows a sustained sinus rhythm.
[3] An ineffective cardioversion means that normal sinus rhythm could not been restored at the end of the procedure or that the last ECG before discharge indicates a reversion to AF.

This document is confidential and proprietary of Volta Medical. All copy is considered uncontrolled. User must verify the revision of uncontrolled copy before use.

25. Significant congenital anomaly or medical problem that in the opinion of the investigator would preclude enrollment
26. Enrollment in an investigational study evaluating another device, biologic, or drug
27. Presence of intramural thrombus, tumor or other abnormality or condition that precludes vascular access, or manipulation of the catheter
28. Life expectancy or other disease processes likely to limit survival to less than 12 months
29. Acute Covid-19 infection (fever and/or biological inflammatory syndrome, and positive test documented)

*Only TEE is acceptable in the United States to screen for LA thrombus. If a subject cannot have a TEE performed, then intracardiac echocardiography at the beginning of the procedure to screen for LA thrombus is acceptable.

5.2.3 – Criteria and procedures for withdrawal or termination of a subject's participation

A patient's participation ends if any of the following occurs:

- At the end of 12-month follow-up period or if relevant, after resolution of a study-device-related adverse event ongoing at the time of the 12-month follow-up
- If the patient does not undergo an AF catheter ablation
- At any time, in the event of withdrawal of consent or cooperation by the patient
- At any time, if the patient is lost to follow-up
- At any time, in the event of patient's exclusion from the study, required for safety reasons according to the investigator's judgement
- In the event of the patient's death
- At any time, in the event of withdrawal of approval from relevant Ethics Committee or IRB

In each of the above termination cases, the appropriate case report form must be completed to document and justify the end of a patient's participation in the study.

If an adverse effect due to the implementation of the protocol is observed, the study may be suspended at any time by the sponsor.

Premature termination of the study may occur motivated by a general decision (Ethics Committees, IRBs, regulatory Competent Authorities, Data Safety Monitoring Board) for safety reasons related to the use of the device or study procedures.

A site may be terminated for the following reasons: failure to comply with the conditions of the study protocol and/or failure to accurately complete case report forms.

5.2.4 – Expected total duration of the clinical investigation

The clinical investigation will not begin in any center until:

- Approval has been obtained from the relevant Ethics Committees or IRB, including approval from national and/or local Ethics Committees or IRBs as required by current national and/or local laws on the rights, safety, and welfare of human subjects.
- Approval or no objection has been obtained from regulatory Health Authorities, as required by current national and/or local laws on the rights, safety, and welfare of human subjects.
- Liability coverage has been obtained by the sponsor, as required by current national and/or local laws on the rights, safety, and welfare of human subjects.
- Investigator agreement has been obtained from the investigator(s) in the center, as required by the Declaration of Helsinki and national/local laws on the rights, safety, and welfare of human subjects.

The expected total duration of the study is 41 months:

This document is confidential and proprietary of Volta Medical. All copy is considered uncontrolled. User must verify the revision of uncontrolled copy before use.

CONFIDENTIAL

- 4-month set-up
- 22-month enrollment
- 12-month follow-up
- 3-month database lock, analysis, and close-out

5.2.5 – Expected duration of participation for each subject

Each patient is expected to participate in the study for up to 16 months (cf. Table 8).

The patient's participation in the study begins when patient is determined eligible to participate and he/she has signed the consent form, after having received the appropriate and mandatory information about the study.

The patient's participation in the study ends if any of the situations described in section 5.2.3 – Criteria and procedures for withdrawal or termination of a subject's participation occurs.

5.2.6 – Number of subjects required to be included in the clinical investigation

The number of subjects to be included is 374 patients, i.e. 187 patients in each group (cf. 6.2 – Sample size). The maximum number of subjects to be included in the United States is 80.

5.2.7 – Estimated time required to include this number (i.e. the period of enrollment)

The estimated enrollment period is 22 months.

In case of significant changes in the inclusion phase duration, the relevant Ethics Committees, IRBs, and regulatory Health Authorities, as well as all concerned investigators, will be promptly notified.

At any time, the sponsor may interrupt or suspend enrollment of patients in the study for clinical/safety reasons (such as if an unanticipated adverse effect is observed or suspected) related to the device under investigation. In that case, the relevant Ethics Committees, IRBs, and regulatory Health Authorities, as well as all concerned investigators, will be promptly notified.

In addition to the sponsor, any Ethics Committee, IRBs, or regulatory Health Authorities may also interrupt or suspend enrollment of patients in the study for clinical/safety reasons related to the devices under investigation.

5.3 – Study procedures

5.3.1 – Screening and enrollment

Patients must be informed about the investigation and the potential benefits and risks of the study prior to enrollment. Only those patients who voluntarily provide written consent to participate will be eligible for enrollment.

The following procedures must be conducted **before** inclusion of the patient in the study:

- Evaluate the patient to determine conformance with the inclusion and exclusion criteria. Source data that demonstrate conformance with inclusion criteria should be retained at the investigational site.
- Obtain written informed consent.

Patients are considered enrolled into this clinical study once they have signed an informed consent and met all eligibility criteria. Patients who do not sign an informed consent or do not meet eligibility criteria upon further testing are not eligible and should not be enrolled in the trial.

CONFIDENTIAL

After having obtained the informed consent from the patient (and before ablation):

- Fill-out enrollment in appropriate eCRF
- Obtain and record the following data in appropriate eCRF:
  - Patient's demographics
  - Mandatory documentation of the maximum sustained AF duration or number of cardioversions (ECG/physician's letter)
  - Current vitals (physical examination, 12-lead ECG)
  - Routine clinical data including type of AF, LA diameter/surface (echo) or volume (scanner), CHA_2DS_2-VASc score, comorbidities, etc.
  - Patient's current medications
- Provide the patient with Quality-Of-Life questionnaires (SF-36 and AFEQT) and report answers in appropriate eCRF
- For US subjects: A TEE screening will be performed within 48 hours of the ablation procedure unless data from a TEE performed prior to enrollment is available and the TEE was performed within 48 hours of the ablation procedure.
  - If a patient cannot undergo TEE, the use of intracardiac echocardiography at the start of the ablation procedure to exclude LA thrombus is acceptable.

5.3.2 – Randomization

After successful enrollment, the patient will be automatically randomized to one of two arms through the eCRF system: "Anatomical" arm (PVI) or "Tailored" arm (Dispersion + Tailored-PVI). In Europe, randomization will be stratified in order to balance the treatment arms in terms of patients with persistent AF (< 12 months) and long-standing persistent AF (≥ 12 months).

Subjects will not be informed of their randomization assignment.

5.3.3 – Pre-ablation Medication

Treatment with antiarrhythmic medications should be stopped before ablation.

Continuous anticoagulation with warfarin (INR 2-3) or NOAC for > 4 weeks prior to ablation is mandatory. Anticoagulation should not be interrupted before the ablation procedure, unless specifically instructed by the investigator. Only 1 or 2 doses of oral anticoagulation may be interrupted by the investigator.

5.3.4 – Index procedure

The subject will be scheduled to undergo study treatment after the subject has met all inclusion and exclusion criteria, has signed the study informed consent form, has completed the baseline assessments and has been randomized.

AF induction

If the patient does not present in spontaneous sustained AF at the beginning of the procedure, sustained AF must be induced by atrial burst pacing, either with or without the additional infusion/injection of isoproterenol (20 microg/min) ± adenosine (according to site practice).

In the Tailored group, should AF not be inducible or be inducible and non-sustained, the patient will undergo conventional AF ablation per physician discretion. This patient/case will not be considered in the per-protocol analysis.

This document is confidential and proprietary of Volta Medical. All copy is considered uncontrolled. User must verify the revision of uncontrolled copy before use.

CONFIDENTIAL

Mapping (only for Tailored group)

For the Tailored group, *careful biatrial high density dispersion mapping* must be performed as the first step of the procedure. A biatrial mapping shorter than 20 minutes is considered incomplete. An incomplete mapping with failure to terminate the AF is considered a deviation of the protocol (cf. X – DEVIATIONS FROM CLINICAL INVESTIGATION PLAN).

Operators must map each region of both atria carefully. Electroanatomic color maps (i.e. voltage maps with minimal interpolation area) or the density of electroanatomic dots on 3D shells can be visually used to verify that the biatrial mapping has been completed. Operators must insist in difficult areas: ridge, anterior Right Superior Pulmonary Vein, area around the transseptal puncture, CS, right septum, area between RA Appendage (RAA) and septum.

Operators should move the mapping catheter slowly and remain in each position for at least 2-3 seconds. If the upper frame of VX1 interface changes from blue to orange/red, the corresponding dipoles must be manually tagged on the 3D shells (white/golden dots respectively – cf. VX1 instructions-for-use). Accuracy and relevance of dispersion regions should be confirmed by the operator by checking EGMs visually. If VX1 identifies an area of dispersed electrograms that the operator does not agree with, this should be documented.

If a dispersion area is detected, the operator must stay and move slowly in this area: a high number of dots is required to define the precise boundaries of the dispersion area.

Intraprocedureal anticoagulation

Use of intraprocedural anticoagulation consistent with your standard practice should be used during the index ablation. It is recommended that heparin should be administered prior to or immediately following transseptal puncture during AF catheter ablation procedures and adjusted to achieve and maintain an ACT of at least 300 seconds.

Ablation procedure in the Tailored group

In the Tailored group, the procedure endpoint is *sinus rhythm conversion by ablation* at dispersion regions mapped during AF and potential subsequent AT(s).

Once mapping is complete:

1) The operator starts by ablating all dispersion areas. Tailored-PV-encircling[4] along dispersion regions is recommended during dispersion areas ablation (cf. Figure 2). Ablation should always be done in a manner to either cover the dispersed electrograms or encircle a large area of dispersed electrograms (cf. Figures 3 and 4)
2) The operator then completes the isolation of the 4 PVs
3) The electrical connection of the 4 PVs is checked at the end of the procedure according to each site routine practice. Additional perivenous ablation can be performed to electrically disconnect the connected PV(s).

[4] Tailored-PVI or Tailored-PV-encircling corresponds to PVI or PV-encircling along dispersion areas around the PVs in order to optimize the flow of the procedure.

This document is confidential and proprietary of Volta Medical. All copy is considered uncontrolled. User must verify the revision of uncontrolled copy before use.

CONFIDENTIAL

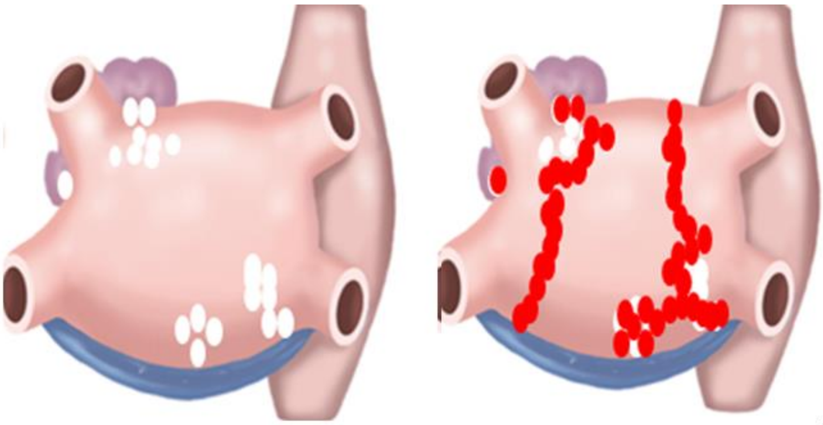

Figure 2: Tailored-PVI after Tailored-PV-encircling

In case of failure to terminate AF after ablation of all dispersion areas, *at least one re-mapping/re-ablation is mandated, and two complete re-mappings* are recommended. If the patient did not convert to sinus rhythm with ablation, a DC electrical cardioversion is performed.

If sinus rhythm is restored before ablation at all dispersion areas, the completion of the ablation set is mandatory, except in the atrial regions positioned close to the esophagus, phrenic nerve, AV node and/or sinus node. This list is not exhaustive, the operator can identify and document any other potentially hazardous areas for ablation.

When a sustained AT results from the ablation of all dispersion areas, mapping and ablation of AT must be performed with the use of activation maps ± VX1 ± pacing maneuvers.

In case of linear ablation performed for macro-reentry (roof, perimitral, cavo-tricuspid isthmus (CTI) flutter), lines should be validated by establishing a post-ablation bidirectional block. The impossibility of performing the line validation must be documented. No line validation is required without documented macro-reentry.

If two ablation areas are close (< 2 cm) or if one ablation area is close (< 2 cm) to an electrically neutral structure (PV, valva), it is recommended to join them in order to reduce the risk of creating areas prone to AT – "Ablate & Connect" strategy (cf. Figure 3).

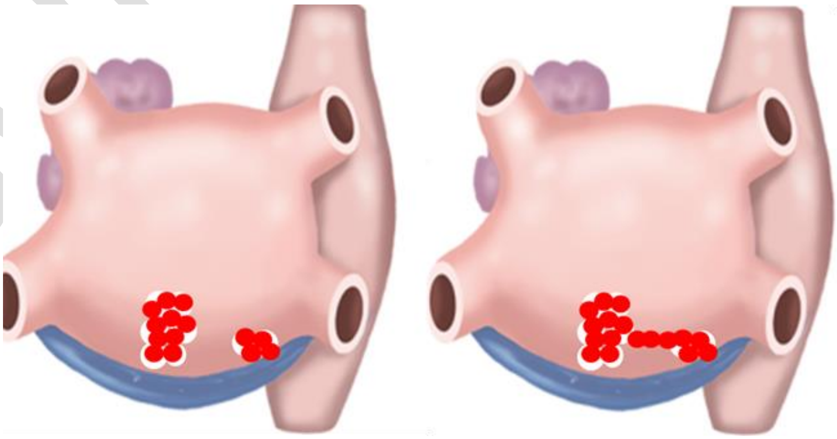

Figure 3: Connection of close dispersion areas (Ablation tags diameter on the 3D shells = 6 mm).

CONFIDENTIAL

In the case of a wide and extensive dispersion area, white dots can be encircled so as to electrically isolate each dispersion zone – “Circle & connect” strategy (cf. Figure 4).

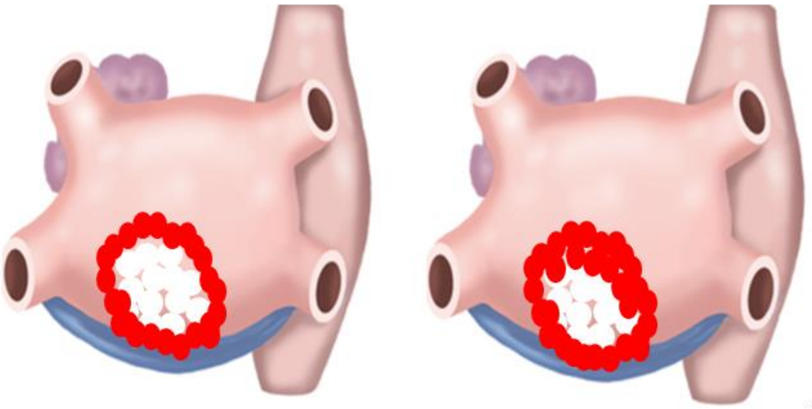

Figure 4: Centripetal ablation

The Tailored ablation protocol is summarized in Figure 5.

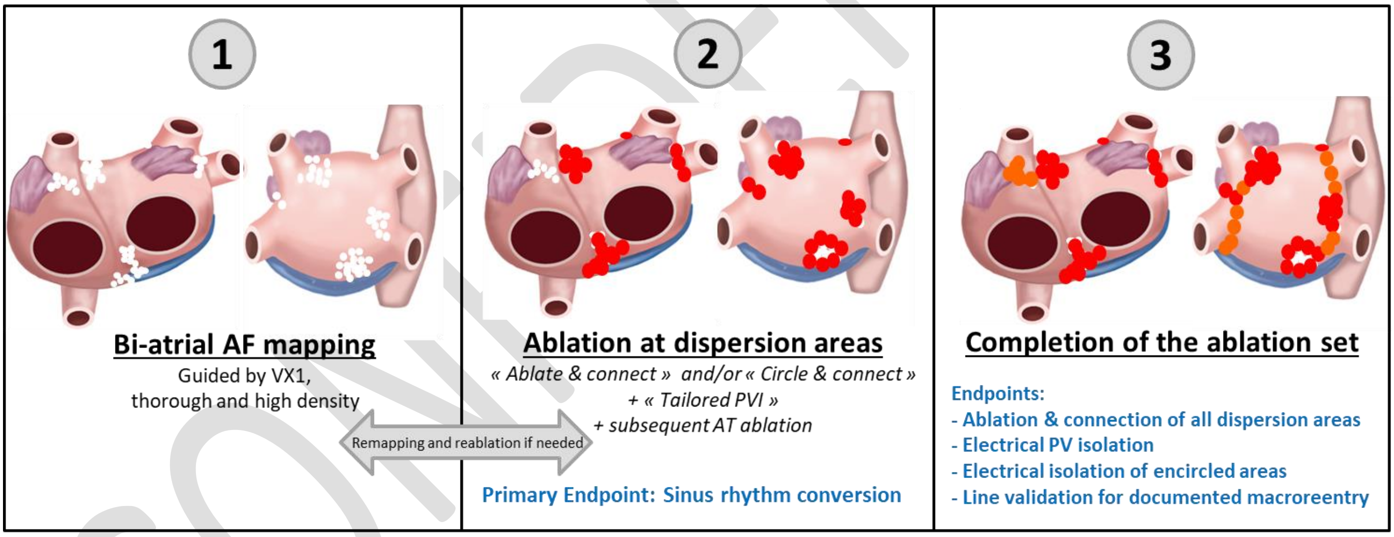

Figure 5: The Tailored protocol

<u>Ablation procedure in the Anatomical group</u>

In the Anatomical group, the procedure endpoint is *<u>isolation of all four PVs</u>*, documented according to each site routine practice (e.g. bidirectional block with mapping catheter).

The operator creates a contiguous circular lesion around each pulmonary vein antrum (1-2 cm from the ostia) with point-by-point applications of RF energy. If the patient is not converted to sinus rhythm at the end of the procedure, a DC electrical cardioversion must be performed.

No RF applications outside the PV antra are allowed (cf. Figure 6 (b)). RF applications at the carinas are allowed (cf. Figure 6 (a)).

CONFIDENTIAL

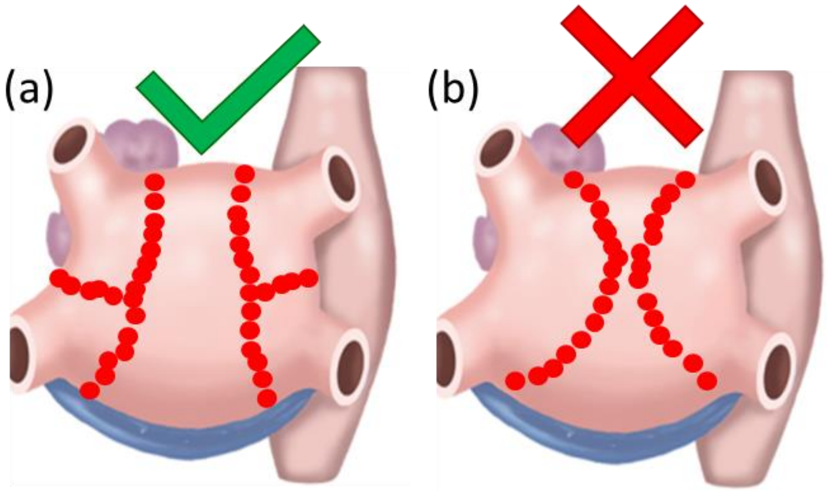

Figure 6: RF applications (a) allowed at the carinas and (b) forbidden outside PV antra

<u>RF application recommendations</u>

Operators will select their contact Force working ranges based on experience during the roll-in cases, review of literature, clinical data. Recommendations: 5-20 g.

High power – short duration RF applications are highly recommended (40-50 watts everywhere except in the CS or close to the esophagus). Table 5 gives recommendations for RF application duration and power. Failure to follow them does not constitute a deviation, as <u>RF application parameters are left to the operator's discretion</u>.

Table 5: RF application duration and power recommendations

Parameter	LA anterior wall / Septum / PV regions / floor	LA ridge / mitral isthmus	LA roof	LA posterior wall	CS	RA	RAA
Power (watts)	40-50	40-50	40-50	25-50	25-30	40-50	40-50
Maximal application time in case of 50 watts applications	30 sec	30 sec	20 sec	15 sec	NA	30 sec	30 sec
Ablation index	500-550	500-550	450-500	350-400	400	450-500	500-550
LSI	5.5-6	5.5-6	5	4	4	5	5.5-6

Number of applications inside/outside these ranges must be quantified (cf. Table 6). The ablation tag diameter should be 6 mm. All tags must be contiguous.

CONFIDENTIAL

Table 6: Color code for ablation tag

Tag	Ablation index	LSI
Light red	350-500	4-5
Red	500-600	5-6.5
Dark red	>600	>6.5

<u>AT ablation</u>

In case of AT, mapping and ablation of AT should be performed <u>only in the Tailored group</u>. In case of documented history of common flutter or if a subject develops common flutter during the procedure, a cavotricuspid isthmus (CTI) line is recommended regardless of the randomization group.

In case of regularization into a non-common flutter in the Anatomical group, no mapping and ablation should be performed. However, a DC electrical cardioversion must be performed.

<u>Data collection</u>

Table 7: Main data collected during procedure (complete list in eCRF)

Data	Description
Procedure times	Procedure start: Time of insertion of first sheath for vascular access<br>Procedure end: Time of removal of sheaths
Voltage maps	Baseline Voltage maps performed blindly by the operator.<br>In the Tailored arm, the baseline Voltage map is performed during VX1-guided mapping.<br>In the Anatomical arm, the baseline Voltage map is performed during fast-anatomical mapping.
RA/LA volumes	RA/LA volumes in mL without veins
Mapping times	Time of start and end of each AF mapping for Tailored group<br>Time of start and end of fast-anatomical mapping for Anatomical group
Ablation times	Time of start and end of each ablation cycle
Mapping picture	<u>Only for Tailored group</u><br>Picture of the biatrial shell with electroanatomic dots and VX1 dots before ablation start
Fluoroscopy time and dose	Total fluoroscopy time and dose for the entire procedure
RF times	Total RF ablation time and RF time to AF termination
RF Power	Min, Max and most commonly used RF power value
Ablation surface area	Total ablation surface area and ablation surface area to terminate AF, when the 3D navigation system allows it
Post-ablation picture	Picture of the biatrial shell with ablation tags for both arms and crucial dots for tags for both arms
Crucial dots	Crucial dots* number and location (cf. Figure 7)<br><br>**Crucial dots = AF/AT termination points or 20-ms cycle length increase*

CONFIDENTIAL

Ablation of VX1-tagged regions	Location and justification of VX1-tagged regions not ablated by the operator
AT data	Number, mechanism, and location (cf. Figure 7) of ATs
Procedure endpoints documentation	Pictures confirming PV electrical isolation, AF termination, SR conversion, lines validation (if applicable)
Medication administration	All cardiac medications administered during the procedure
Devices and equipment	Devices and equipment used during the procedure including diagnostic and ablation catheters, 3D navigation and EP recording systems

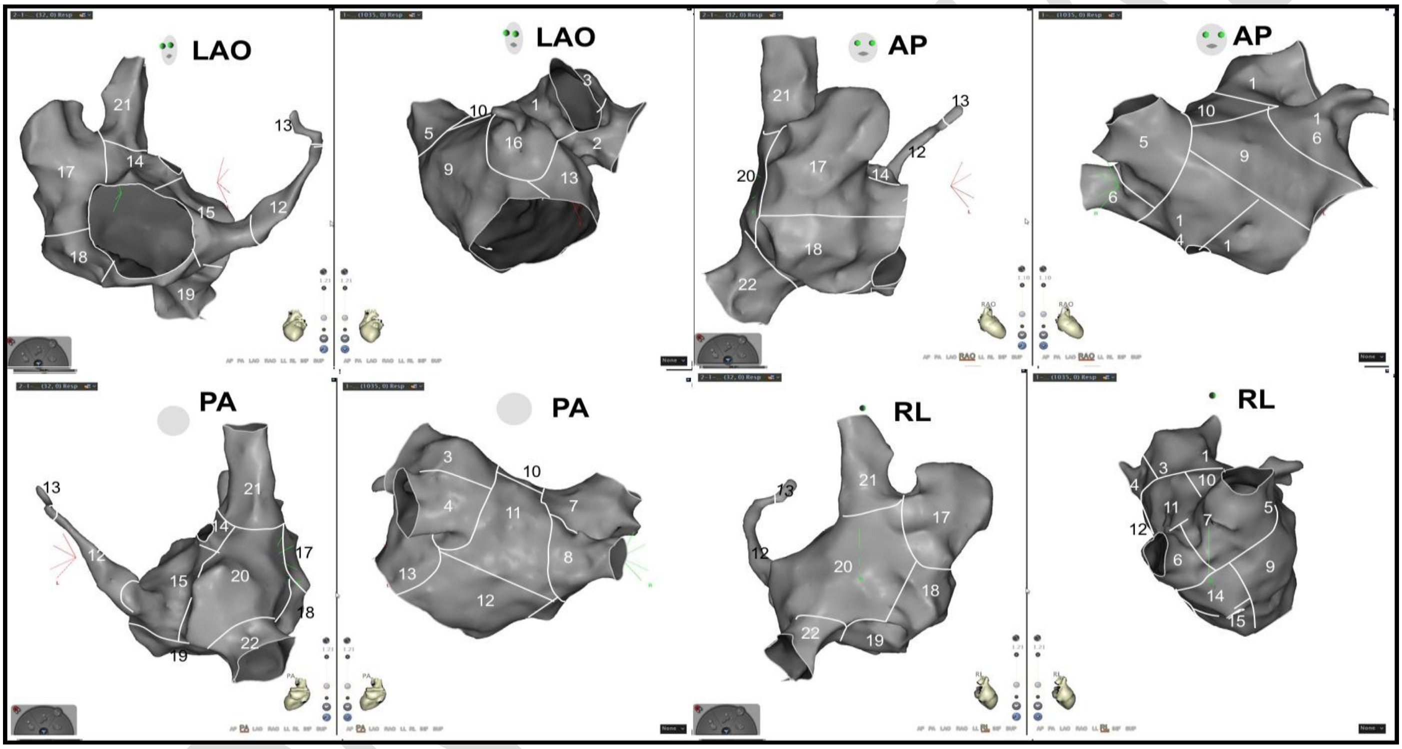

Figure 7 : Bi-atrial segmentation used for characterization of dispersion tagged regions

Region 1: Left superior pulmonary vein anterior antrum – Ridge.
Region 2: Left superior pulmonary vein inferior antrum-Ridge.
Region 3: Left superior pulmonary vein posterior antrum.
Region 4: Left inferior pulmonary vein posterior antrum.
Region 5: Right superior pulmonary vein anterior antrum.
Region 6: Right inferior pulmonary vein anterior antrum.
Region 7: Right superior pulmonary vein posterior antrum.
Region 8: Right inferior pulmonary vein posterior antrum.
Region 9: Anterior wall from mitral annulus to roof.
Region 10: Roof.
Region 11: Posterior wall.
Region 12r: Mid-coronary sinus.
Region 12l: Left atrial floor.
Region 13r: Coronary sinus distal.
Region 13l: Mitral isthmus.
Region 14r: High right atrial septum.
Region 14l: High left atrial septum.
Region 15r: Low right atrial septum.
Region 15l: Low left atrial septum.
Region 16: Left atrial appendage.
Region 17: Right atrial appendage.
Region 18: Lateral right atrial appendage.
Region 19: Cavo-tricuspid isthmus.
Region 20: Posterior right atrium.
Region 21: Superior cava vein.
Region 22: Inferior vena cava

This document is confidential and proprietary of Volta Medical. All copy is considered uncontrolled. User must verify the revision of uncontrolled copy before use.

Pre-discharge and post-procedure medication

Post-procedure drug therapy will include re-initiation and/or continuation of any pre-procedure cardiac medications and/or prescription of supplemental anti-arrhythmic medications, under the direction of the investigator. All medications will be documented in the appropriate section of the eCRF.

Systemic anticoagulation with warfarin or a NOAC is recommended for at least 2 months after the procedure. Decisions regarding continuation of systemic anticoagulation more than 2 months post ablation should be based on the patient's stroke risk profile and not on the perceived success or failure of the ablation procedure.

The use of antiarrhythmic medications is allowed for the first 3 months (post-ablation blanking period) after the index procedure and discouraged thereafter.

The use of diuretics prophylactically, especially for long procedures regardless of the study arm, is recommended.

5.3.5 – Trans Telephonic event Monitor (TTM)

All patients will receive a portable ECG device (KardiaMobile, AliveCor) that they will keep throughout their participation in the Study. They will be asked to perform one 30-second ECG recording per week, and whenever they have arrhythmia symptoms. All TTM recordings will be automatically uploaded in a secured platform for blind review by the ECG core lab.

5.3.6 – Follow-up period

All patients will be followed-up for 12 months with scheduled in-office visits at 3, 6 and 12 months. A 3-month blanking period will be applied after the index procedure, during which early recurrences of AF/AT will not be counted.

All patients will be required to perform a 24-hour Holter prior or subsequent to each in-office visit at 3, 6 and 12 months post-procedure. The 24-hour Holter recording must be performed within the same time windows as the in-office visit specified in Table 8.

At each in-office visit (3, 6 and 12 months or unscheduled follow-up visit), the following assessments/procedures will be performed:

- Diagnostic 12-lead ECG
- Assessment of Holter report if available (only during scheduled visits)
- QoL questionnaires (only during scheduled visits)
  - It is recommended that QoL questionnaires be completed prior to any other follow-up procedures in order to reduce influencing the subject's responses.
- Concomitant cardiac medications
- Adverse event documentation

At the 12-month visit, a blinding assessment should be done by asking the subject which arm of the study they believe they are in. The subject's response should be recorded.

If AF and/or AT recurrences are observed during follow-up, further treatment shall be at the discretion of the physician (e.g. drugs or re-ablation or cardioversion). Re-ablations after the 3 months blanking period and before the end of the 9th month after the first procedure are highly recommended.

In the event of aggravating circumstances (new Covid-19 outbreak), these visits can be carried out remotely.

This document is confidential and proprietary of Volta Medical. All copy is considered uncontrolled. User must verify the revision of uncontrolled copy before use.

CONFIDENTIAL

5.3.7 – Redo procedure

Only one re-ablation procedure is allowed per-protocol between the end of the 3rd month and the end of the 9th month after the first procedure (6-month window).

Re-ablation procedure in Tailored group

In the Tailored group, a re-ablation procedure will be performed using the same approach as the first index procedure. The redo procedure endpoint is also *sinus rhythm conversion by ablation* at dispersion regions and potential subsequent AT. In addition, a CTI anatomical line is allowed.
If the repeat procedure is performed for an AT recurrence, AF induction is not required at the beginning of the procedure.

Re-ablation procedure in Anatomical group

During a repeat ablation in the Anatomical group, PV reconnections will be checked, and potentially reconnected PV will be re-isolated.

A maximum of two additional anatomical lines are allowed (0, 1 or 2 lines): roof and/or mitral isthmus and/or CTI lines or posterior box isolation (no other lines are allowed).

The redo procedure endpoint is *isolation of all four PVs and lines validation*.

In case of regularization into AT during anatomical re-ablation, mapping (without VX1) and ablation of the AT is allowed. If the repeat procedure is performed for an AT, it can be mapped (without VX1) and ablated.

5.3.8 – Blanking periods

After the index procedure, a 6-week absolute blanking period will be applied, during which a repeat procedure is not allowed, only cardioversions are allowed if necessary.

After this absolute blanking period and before the end of the standard 3-month blanking period, a repeat procedure is discouraged but not prohibited. The decision to perform a repeat procedure before the end of the 3-month blanking period is left to the operator’s discretion. If a repeat procedure is performed during the 3-month blanking period, the recurrence and the repeat procedure are counted as occurring at 3 months.

After a repeat procedure, a 2-week blanking period will be applied, during which AF/AT recurrences will not be counted. Any re-repeat procedures will be performed outside the study according to the operator’s preferred approach and will result in the data being censored at the time of the re-repeat procedure. The patient will however stay in the study for safety purposes.

5.3.9 – Visit schedule

Study visits that do not occur within time windows in Table 8 will be considered protocol deviations.

Table 8: Time windows for study visits

**Visit**	Screening, enrollment, and randomization	Index procedure	3-month follow-up	Redo procedure (if required)	6-month follow-up	12-month follow-up
**Timing**	Prior to index procedure	0 – 90 days after enrollment	2M + 1 day to 3M after index procedure	3M to 9M after index procedure	6M ± 30 days after index procedure	12M ± 30 days after index procedure

This document is confidential and proprietary of Volta Medical. All copy is considered uncontrolled. User must verify the revision of uncontrolled copy before use.

CONFIDENTIAL

5.3.10 – Summary of required clinical assessment and data collection

Table 9 summarizes study visits and required assessments and data collection.

Table 9: Schedule of events

Event	Screening	Enrollment	Index procedure	3-month follow-up visit	Redo procedure (if required)	6-month follow-up visit	12-month follow-up visit	Unscheduled follow-up visit
Inclusion and exclusion criteria assessment	X							
Informed consent	X							
Demographics, medical history, cardiac diagnosis		X						
Documentation of AF (ECG, physician's letter)		X						
12-lead ECG		X		X		X	X	X
Physical exam		X		X		X	X	X
Concomitant cardiac medications		X	X	X	X	X	X	X
AF assessment (AF presence and/or AF recurrence since last follow-up)		X	X	X	X	X	X	X
QoL questionnaire		X		X		X	X	
24h ambulatory continuous ECG (Holter)				X		X	X	
Adverse Events review		X	X	X	X	X	X	X
AF interventional procedure			X		X			

CONFIDENTIAL

5.4 – Investigators and sites

Investigators selected to participate in this study:

- must be experienced in AF/AT ablation and must have been validated by the coordinating investigator
- must be willing to accept the responsibilities of investigator
- must allow the Sponsor's designated monitors and representatives to review the records pertaining to this study, source documentation and patient informed consents
- must allow eventual internal or external Quality Assurance visit

Each investigator of this study must be trained to the visual detection of dispersion, the use of VX1 and to this protocol. In addition, each investigator must have participated to at least one case by a Volta Medical EP (live or video) and must have performed at least 2 training or roll-in cases in compliance with the Tailored group ablation workflow before inclusions can begin. These patients will not be included in the study and will not be followed-up after ablation.

The presence of a Field Technical Engineer (FTE) from Volta Medical is mandatory for at least the initial cases of the study in order to support investigators.

Considering the possibility of a new Covid-19 outbreak leading to restrictions on travel and access to hospitals, it should be possible for Volta Medical FTEs to support cases remotely, by means of appropriate remote video systems.

Before participating in the study, all investigators must agree to respect and fulfil the terms of this investigational plan and sign and date an Investigator Agreement (cf. Appendix 18.5).

The coordinating investigator of this study is:

Prof. Isabel DEISENHOFER
German Heart Centre Munich
Lazarettstraße 36
80636 Munich, GERMANY

The updateable list of expected investigators and Investigation sites is provided in Appendix 18.7.

During the progress of the Study, according to local regulatory requirements, additional centers and/or other investigators may take part in the Study. In that case, the relevant Ethics Committees, IRBs, and Regulatory Authorities, as well as all investigators, will be notified.

5.5 – Steering Committee

The study is driven by a Steering Committee, whose members are listed in Appendix 18.8. This committee will be actively involved in the investigation and review its progress at regular intervals. At any time, this committee may request that the investigation be put on hold or even terminated for safety, ethical or other reasons.

5.6 – Monitoring plan

The study will be monitored periodically at each enrolling site per the study monitoring plan, for the purpose of:

- Verifying compliance to the protocol (deviations) and applicable regulations (signed informed consent forms),
- Verifying that study documentation exists,
- Verifying case report form data to original entries in source files (discrepancies, missing data).

Volta Medical delegates the responsibility for ensuring proper monitoring of the Study to Labcorp.

The detailed monitoring plan is developed by Labcorp and validated by Volta Medical.

CONFIDENTIAL

Appropriate qualified and trained Labcorp employees or representatives will monitor the study according to Labcorp standard operating procedures.

Qualification visits: the monitor will evaluate the investigation sites to ensure that they have the appropriate qualifications and resources and have access to a sufficient number of patients to participate in the Study.

Initiation visits: the monitor will then initiate each Investigation site and will ensure that the Principal Investigator and his team:

- have received and understood the requirements and content of the Clinical Investigation Plan, the Investigator's Brochure, the Informed Consent Form, the eCRF and the Instructions for Use,
- have been trained to the use of the device studied,
- have been informed of their responsibilities.

Monitoring visits: each investigation site will undergo in-site and remote monitoring visits during the Study duration, as defined in the monitoring plan.

Termination visits: closing activities will be carried out by the monitor at each investigation site.

Volta Medical and representatives of regulatory health authorities are permitted to inspect the study documents (protocol, case report forms, study-related medical records, study correspondence, etc.). In addition to ongoing monitoring of the study, GCP audits by the Sponsor or its representatives are also permitted. All attempts will be made to preserve subject confidentiality.

VI – STATISTICAL CONSIDERATIONS

6.1 – Design, statistical methodology and analytical procedures

Standard summary statistics will be calculated for all study variables (demographics and outcomes) by study group and for all patients. For continuous variables, statistics will include means, standard deviation and 95% confidence intervals for means when assumptions of normal distribution are not violated. Median and quartiles may also be presented. Categorical variables will be summarized using frequency distributions.

The analysis for the primary endpoint will be a modified Intention-To-Treat (mITT) analysis, allowing exclusion of some randomized subjects in a justified way:

- Patients that are deemed ineligible after randomization or that do not have any AF ablation will be excluded
- Patients in the Tailored group for whom AF cannot be induced and who undergo conventional ablation will be included in this analysis
- Patients lost to follow-up (death, disability, relocation, or drop-out) during the 3-month blanking period will be excluded
- Patients who complete at least their 3-month follow-up but who are lost to follow-up (death, disability, relocation, or drop-out) before the 12-month follow-up will be included in the analysis and the data will be censored
- Patients who undergo a 3rd ablation procedure (re-repeat procedure) will be included in the analysis but the data will be censored at the time of the 3rd procedure.

A per-protocol (PP) population will also be evaluated and will serve as supportive analyses to the mITT analysis. In the PP analysis, the population will be analyzed according to the treatment actually received at the start of the study, and will consist of patients that met all the inclusion criteria, none of the exclusion criteria, were successfully treated, had no important protocol deviations and attended all the scheduled follow-up visits.

This document is confidential and proprietary of Volta Medical. All copy is considered uncontrolled. User must verify the revision of uncontrolled copy before use.

CONFIDENTIAL

6.2 – Sample size

The primary endpoint is freedom from AF, 12 months after a single ablation procedure. As observed from previously published studies in patients receiving PVI alone for persistent AF, it could be assumed that at most 62% of the patients in the anatomical group will be free from AF at the end of follow-up[5,8]. A previous study conducted on VX1 suggested that at least 77% of the patients of the tailored group may be free from AF at the end of follow-up (i.e. Hazard Ratio (HR) 0.547). In order to confirm these results prospectively in this study, 292 patients (146 in each group) are required to show that time to AF is significantly different between groups (Log-Rank test for a one-sided superiority trial, significance level α=0.025, statistical power of 80%, under the assumption that the hazard rates are proportional).

Assuming a drop-out rate of 22% (no ablation performed or loss to follow up), **374 patients are required overall** (187 in each group).

Sample size calculations performed using SAS Proc Power TwoSampleSurvival (SAS Institute Inc., Cary, NC)

6.3 – Degree of significance and power of the clinical investigation

Assumptions for the superiority endpoint include a one-sided 0.025 alpha level and 80% power.

6.4 – Expected drop-out rate

We expect a maximum drop-out rate of 22% (i.e. a maximum of 82 patients among the 374 patients are expected to fail to complete the 12-month follow-up).

6.5 – Evaluation criteria for primary and secondary endpoints

6.5.1 – Long-term effectiveness primary endpoint

For the primary endpoint, both Tailored (Treatment) and Anatomical (Control) groups will be compared in a superiority context employing a superiority margin of 15%, as established above in the sample size calculations and as the VX1 has demonstrated in a previous study (HR 0.547).
Formally, the one-sided hypothesis to be tested (Log-Rank test) is:

H_0: HR is over 0.547, indicating that the proportion of patients free from AF recurrences at 3-12 month in the Treatment group is not superior to the proportion of patients free from AF recurrences at 3-12-month in the Control group plus 15% margin.

$$H_0: HR > 0.547$$

H_1: HR equals or is less than 0.547, indicating that the proportion of patients free from AF recurrences at 3-12 month in the Treatment group is superior to the proportion of patients free from AF recurrences at 3-12-month in the Control group plus 15% margin.

$$H_1: HR \leq 0.547$$

The null hypothesis will be rejected if the p-value is less than the significance level of 0.025.

If the Treatment group is found to have a statistically significantly greater freedom from AF rate than Control, the Treatment will be considered superior relative to Control.

In addition, the time to AF recurrence in each group will be visually compared by Kaplan-Meier curves.

The impact of any baseline demographic factor, which is found to be significantly different between the treatments, will be assessed.

This document is confidential and proprietary of Volta Medical. All copy is considered uncontrolled. User must verify the revision of uncontrolled copy before use.

CONFIDENTIAL

6.5.2 – Other secondary and exploratory endpoints

For all other survival data, one-sided and two-sided Log-Rank test will be applied at a significance level of 0.05 (secondary/exploratory endpoints 2-9). The secondary efficacy endpoints will be evaluated in a pre-specified order following a gatekeeping approach so that subsequent endpoints will be evaluated if and only if the previous endpoint was found to achieve statistical significance. Thus, no correction for multiple testing will be required. Further details including the ordering of endpoints will be provided in the statistical analysis plan (SAP).

For quality of life (endpoint 10), a linear mixed model for repeated data will be used to examine the difference between groups over time.

For all other endpoints:

The continuous variables will each be analyzed by a Student's t-test, and equivalent non-parametric method, the Wilcoxon–Mann–Whitney test, will be used in case of violation of the normality assumption. Chi-square test or Fisher exact test will be used for the categorical data.

6.6 – Interim analysis

This trial will employ an adaptive design for the blinded re-estimation of the sample size. To ensure the study is adequately powered for the superiority comparison for the primary outcome measure, the assumptions regarding the rates of AF in both study arms will be verified after 50% of the originally planned total patient sample have complete primary endpoint information. A conditional power approach for sample-size re-estimation using the Mehta-Pocock[25] approach will be utilized. Following this interim assessment, the sample size may only remain the same or be increased. Details of the statistical calculations to be utilized in support of the interim analysis will be provided in the study Statistical Analysis Plan (SAP). The SAP will also provide details regarding the operational procedures for conducting, reporting, and acting on the results of the analysis.

A third-party, independent statistician will perform this analysis to minimize operational bias. After the analysis, the statistician will provide a recommendation to the Steering Committee and Volta Medical. The recommendation will also be shared with senior, non-operational management staff that have a strict business need to review and act on the results. None of the clinical operational staff or study investigators will have access to the interim data on which the analysis is based in order to further minimize operational bias. The scientific soundness of the study is not likely to be impacted as the only potential change to the study as a result of this analysis is an increase in sample size.

6.7 – Missing data and sensitivity analysis

Subjects who miss a study visit should be contacted immediately by the Investigational site to determine the reason for the missed visit and to reschedule the visit as soon as possible to meet the study visit window. If the subject cannot be located after 3 attempts by phone, email and/or letter, then the subject will be considered lost to follow-up. All attempts to contact the subjects will be documented and retained in the subject study record.

With regard to the main judgement criteria, data will be censored if the patient is lost to follow-up (death, disability, relocation, or drop-out).

As the burden of study participation does not differ between the tailored and the anatomical arm, we expect all study losses to be non-differential with respect to treatment assignment. Thus, our results will not be biased as a result of these losses.

Additional supportive analyses will be performed on the primary endpoint to assess the effect of missing data.

For missing demographic data, the data will not be replaced because it has no weight on the main judgment criterion.

A rigorous monitoring is expected, any missing data will be filled in during these visits.

This document is confidential and proprietary of Volta Medical. All copy is considered uncontrolled. User must verify the revision of uncontrolled copy before use.

CONFIDENTIAL

6.8 – Minimum and maximum number of subjects to be included for each site

A maximum of 75 inclusions per site (20% of inclusions) will be allowed in order to prevent site-effect. This limit will ensure a minimum of 5 enrolling sites.

6.9 – Poolability of Sites

All investigational sites will follow the requirements of standardized data collection procedures and forms. The primary efficacy and safety endpoints will be presented separately for each site using descriptive statistics. Poolability of the primary efficacy endpoint across investigational sites will be evaluated using a logistic regression model with a fixed effect for site. If there is a significant effect of site, analysis will be repeated using site as a random effect. Sites enrolling fewer than 5 subjects will be combined to form one quasi site. Additional exploratory analyses may be performed to understand any variations in outcome by site. Assessment of poolability by geography (US vs. OUS) will be performed based on logistic regression as described above. As US subjects will only be patients with AF duration of 3 months to < 12 months, only OUS patients with AF <12 months will be included in the poolability by country assessment. As only one catheter type is approved for use in the US, an additional subgroup analysis of each catheter used will assess the variability, if any, by country for patients with the same catheter type.

VII – RISKS DESCRIPTION AND MINIMIZATION

7.1 – Expected clinical benefits

VX1 offers a fast and reliable way to identify patient-specific spatio-temporal dispersion EGMs. Previous research done by Volta suggests that consistently targeting dispersed EGMs has resulted in less recurrence of AF. In addition, research by Volta Medical has shown that targeting of dispersed electrograms identified by VX1 results in lesser surface area ablated than targeting complex fractionated atrial electrograms (CFAEs). In targeting these dispersed electrograms in addition to conventional approaches, patients may benefit from fewer redo AF ablation procedures and less recurrence of atrial arrhythmias.

7.2 – Expected adverse effects of the device

The use of VX1 is not expected to increase the likelihood of any adverse effect that might otherwise occur during a cardiac ablation. Use of the VX1 device has been characterized by longer mapping time than observed in comparable patient populations in literature, however procedure times and ablation time have not shown to be longer. Complications associated with AF ablation procedures are well-documented and anticipated. Risks for cardiac ablation include, but are not limited to those identified in section 14.3 – Anticipated adverse events.

7.3 – Risks associated with participation in the clinical investigation

Subjects randomized into the Tailored arm of the study are expected to receive higher RF times, greater ablation area, and slightly longer procedure times than subjects randomized to the anatomical arm. However, previous research indicates that RF time, ablation area, and procedure time while using the VX1 system are well within accepted industry standards for AF ablation and consistent with other approaches to AF ablation in published literature. The study design assumes that all patients receive the standard of care and expect additional benefits linked to close and repeated follow-ups after AF ablation.

The procedures and methods for data collection required by the protocol do not differ from routine AF ablation and post-ablation follow-up practice, other than filling out a Quality-of-Life questionnaire at baseline and at each follow-up visit.

This document is confidential and proprietary of Volta Medical. All copy is considered uncontrolled. User must verify the revision of uncontrolled copy before use.

CONFIDENTIAL

Use of the device not in conformance with this investigational plan and/or with the instructions in the physician's manual could introduce other issues.

7.4 – Measures to control or mitigate risks

To protect the welfare of subjects, Ethics Committee or IRB approval of the investigation will be obtained for each site prior to the first case at that site.

Investigators selected to participate in this study will have experience in AF ablation. Trained and experienced clinical and technical personnel representing Volta Medical will be made available to support procedures and for troubleshooting.

All areas recommended for ablation by the VX1 system will be reviewed by the investigator, and if the investigator determines that the recommended region is undesirable for ablation the investigators will be instructed to maintain the subject's safety first and avoid ablation of undesirable regions. The frequency of such divergent assessments will be documented and evaluated.

Each investigator will sign an Investigator Agreement stating his/her responsibility to conduct this study according to this investigational plan, to adhere to the records and reporting requirements, and to supervise use of the device.

Through careful design and testing, Volta Medical has attempted to minimize the risk to patients. Testing included: software validation testing, electrical safety and electromagnetic compatibility testing according to IEC 60601 standards on the hardware system.

VX1 Application has received CE mark on 14 JAN 2020, VX1 Hardware has received CE mark on 09 JUL 2020.

Copy of the CE certificates are in Appendix 18.3.

Volta Medical Quality Management System is ISO 13485 certified.

7.5 – Justification of the risk-benefit ratio

Considering the potential risks, the measures taken to minimize risks and the potential benefit to patients, the risk-benefit ratio is deemed acceptable for the use of VX1 and for the conduct of this study.

VIII – DATA MANAGEMENT

Electronic Data Capture (EDC) will be used for this investigation for collecting the data. The EDC/eCRF system will be managed by Labcorp according to Labcorp standards. Confidentiality of patient identity will be ensured.

A complete detailed guideline on data to be recorded for the study and how to fill in the eCRFs will be provided to the Investigators.

Data will be reviewed and cleaned, as well as data queries issued and resolved, in accordance with the Data Management Plan.

Audits or inspections by Competent Authorities and FDA may be carried out.

This document is confidential and proprietary of Volta Medical. All copy is considered uncontrolled. User must verify the revision of uncontrolled copy before use.

CONFIDENTIAL

IX – AMENDMENTS TO THE CLINICAL INVESTIGATION PLAN

Major changes to the protocol will be described in a Clinical Investigation Plan Amendment. The amendment will identify the changes made, the reason for the changes and if it is mandatory or optional to implement the amendment.

The principal investigator will acknowledge the receipt of the amendment and confirm by their signature on the Amendment Signature page that they will adhere to the amendment.

The amendment must be submitted to the relevant Ethics Committees or IRB and to Authorities, where required. Approval from the Ethics Committees or IRB will be required prior to implementation of the amendment.

Any amendment affecting the patient requires that the patient be informed of the changes and a new consent be signed and dated by the investigator and patient prior to the patient's next follow-up.

Changes to, or formal clarifications of, the clinical investigational plan will be documented in writing and provided to investigators. This information will be incorporated when an amendment occurs.

X – DEVIATIONS FROM CLINICAL INVESTIGATION PLAN

10.1 – General

A protocol deviation is defined as any event where the clinical investigator or site personnel did not conduct the study according to the protocol.

The Investigator is not allowed to deviate from the Clinical Investigation Plan.

In emergency situations, deviations from the Clinical Investigation Plan, established in order to protect the rights, safety and well-being of the subjects, may be carried out without authorization of Volta Medical or Ethics Committee/IRB prior approval. Such deviations should be recorded and forwarded as soon as possible to Volta Medical and the appropriate Ethics Committee or IRB.

In particular, every effort should be made to avoid deviations from the protocol including, but not limited, to the following:

1. Inclusion of a patient without appropriate written consent or who does not meet the inclusion criteria or who meets any of the exclusion criteria,
2. Missing any data related to the primary and secondary endpoints,
3. Use of other energy than RF for ablation (cryo/chemical),
4. Ablation other than antral PVI in the Anatomical arm for index procedures (e.g. lines other than ICT, EGM ablation, PV encirclement other than antral),
5. Ablation other than PVI ± lines (as defined in 5.3.7 – Redo procedure) in the Anatomical arm for redo procedures,
6. Failure to terminate AF with incomplete mapping in the Tailored group (mono atrial mapping, too short mapping < 20 minutes),
7. Failure to terminate AF with incomplete ablation in the Tailored group, leaving dispersion areas not ablated, without valid justification (for safety reasons at operator's discretion),
8. Failure to terminate AF without a mandatory second mapping/ablation cycle in the Tailored group,
9. Patient becoming unblinded to his/her randomization assignment,
10. Follow-up being performed outside the time window allowed.

CONFIDENTIAL

10.2 – Procedures for recording, reporting and analyzing deviations from the Clinical Investigation Plan

Protocol deviations shall be reported to the Sponsor regardless of whether medically justifiable or taken to protect the patient in an emergency.

Subject-specific deviations will be reported on the Labcorp Risk and Issues Management System (RIMS).

Non-subject specific deviations (e.g. unauthorized use of a study device by a physician who has not signed an investigator agreement, etc.) will also need to be reported to the Sponsor.

Investigators will also adhere to procedures for reporting study deviations to their Ethics Committee or IRB in accordance with their specific reporting policies and procedures.

For reporting purposes, the Sponsor classifies study deviations as important or non-important. Important deviations are defined as changes in the conduct of the trial from that specified in the protocol that compromise the subject's rights, safety, or well-being, or the completeness, accuracy, reliability or scientific integrity of the study data. The following deviations are considered to be important:

1. Enrollment of a patient without written consent
2. Failure to report SAEs
3. Crossovers between the groups
4. Use of other energy than RF for ablation (cryo/chemical)
5. Ablation other than antral PVI in the Anatomical arm for index procedures (e.g. lines other than ICT, EGM ablation, PV encirclement other than antral),
6. Ablation other than PVI ± lines (as defined in 5.3.7 – Redo procedure) in the Anatomical arm for redo procedures,
7. Failure to terminate AF with incomplete mapping in the Tailored group (mono atrial mapping, too short mapping < 20 minutes),
8. Failure to terminate AF with incomplete ablation in the Tailored group, leaving dispersion areas not ablated, without valid justification (for safety reasons at operator's discretion),
9. Failure to terminate AF without a mandatory second mapping/ablation cycle in the Tailored group,

Deviations not classified as important will be considered non-important. Protocol deviations will be classified by the Steering Committee in a blinded fashion. Patients presenting with at least one important deviation will be excluded from the Per-Protocol (PP) analysis.

10.3 – Corrective and preventive actions and criteria for disqualification of the Principal Investigator

In the event of 2 important deviations at the same investigation site, an assessment will be made by the Sponsor and the site principal Investigator may be disqualified. In that case, the inclusions will be stopped at that site.

XI – DEVICE ACCOUNTABILITY

CONFIDENTIAL

The Principal Investigator of each site shall maintain adequate records of the receipt and disposition of all devices per local institutional procedures and regulations.

XII – STATEMENTS OF COMPLIANCE

12.1 – Ethical and regulatory considerations

The study will be conducted in accordance with the ethical principles mentioned in the Declaration of Helsinki (cf. Appendix 18.1), Good Clinical Practice, the ISO 14155 Standard and applicable laws and country regulations where the study will be performed.

This study would not start until approvals or favorable opinions have been received from applicable Ethics Committees or IRBs and Regulatory Authorities.

Each amendment to this Clinical Investigation Plan must be approved beforehand by the applicable Ethics Committees or IRBs and Regulatory Authorities for the full duration of the Study.

Any requirements imposed by the applicable Ethics Committees, IRBs and/or Regulatory Authorities shall be respected and applied throughout the Study period.

12.2 – Insurance

Provided that this protocol is respected, the civil liability of Volta Medical and all its agents is covered by a contract subscribed by Volta Medical with HDI GLOBAL SE (contracts nos. 0100534514058-200083 for France, 390-76236600-30016 for Belgium, 76237332-03032 for Germany and V-071-369-378-0 for The Netherlands). A copy of the insurance certificates can be found in Appendix 18.2.

XIII – INFORMED CONSENT PROCESS

To participate in the study, the subject must sign and date the Informed Consent Form after having been informed about the nature and purpose of the Study, participation and termination conditions, risks, and benefits, before initiation of any study-related procedures. Patient Information and Patient Informed Consent Form are provided in Appendix 18.4.

A copy of the signed Informed consent Form must be provided to the participating patient.

The patient information and Informed Consent Form will be available in certified translation in all official languages of the countries participating in the Study.

Signed Informed Consent Forms must remain in the patients' study files and be available for verification by the Sponsor or representative at any time.

XIV – ADVERSE EVENTS, ADVERSE DEVICE EFFECTS AND DEVICE DEFICIENCIES

14.1 – Definitions (according to ISO 14155:2020)

Adverse Event (AE)

CONFIDENTIAL

Untoward medical occurrence, unintended disease or injury, or untoward clinical signs (including abnormal laboratory findings) in subjects, users or other persons, whether or not related to the investigational medical device and whether anticipated or unanticipated.
NOTE 1: This definition includes events related to the investigational medical device or the comparator.
NOTE 2: This definition includes events related to the procedures involved.
NOTE 3: For users or other persons, this definition is restricted to events related to the use of investigational medical devices or comparators.

Adverse Device Effect (ADE)

Adverse event related to the use of an investigational medical device.
NOTE 1: This definition includes adverse events resulting from insufficient or inadequate instructions for use, deployment, implantation, installation, or operation, or any malfunction of the investigational medical device.
NOTE 2: This definition includes any event resulting from use error or from intentional misuse of the investigational medical device.
NOTE 3: This includes 'comparator' if the comparator is a medical device.

Serious Adverse Event (SAE)

Adverse event that led to any of the following:

a) death,

b) serious deterioration in the health of the subject, users, or other persons as defined by one or more of the following:

- a life-threatening illness or injury, or
- a permanent impairment of a body structure or a body function including chronic diseases, or
- in-patient or prolonged hospitalization, or
- medical or surgical intervention to prevent life-threatening illness or injury, or permanent impairment to a body structure or a body function.

c) foetal distress, foetal death, a congenital abnormality, or birth defect including physical or mental impairment.

NOTE 1: Planned hospitalization for a pre-existing condition, or a procedure required by the CIP, without serious deterioration in health, is not considered a serious adverse event.

Serious Adverse Device Effect (SADE)

Adverse device effect that has resulted in any of the consequences characteristic of a serious adverse event.

Unanticipated Serious Adverse Device Effect (USADE)

Serious adverse device effect which by its nature, incidence, severity or outcome has not been identified in the current risk assessment.
NOTE 1: Anticipated serious adverse device effect (ASADE) is an effect which by its nature, incidence, severity or outcome has been identified in the risk assessment.

Device deficiency

Inadequacy of a medical device with respect to its identity, quality, durability, reliability, usability, safety or performance.
NOTE 1: Device deficiencies include malfunctions, use errors, and inadequacy in the information supplied by the manufacturer including labelling.
NOTE 2: This definition includes device deficiencies related to the investigational medical device or the comparator.

Device malfunction

This document is confidential and proprietary of Volta Medical. All copy is considered uncontrolled. User must verify the revision of uncontrolled copy before use.

CONFIDENTIAL

Failure of an investigational medical device to perform in accordance with its intended purpose when used in accordance with the instructions for use or CIP, or IB.

Use error
User action or lack of user action while using the medical device that leads to a different result than that intended by the manufacturer or expected by the user.
NOTE 1: Use error includes the inability of the user to complete a task.
NOTE 2: Use errors can result from a mismatch between the characteristics of the user, user interface, task or use environment.
NOTE 3: Users might be aware or unaware that a use error has occurred.
NOTE 4: An unexpected physiological response of the patient is not by itself considered a use error.
NOTE 5: A malfunction of a medical device that causes an unexpected result is not considered a use error.

14.2 – Adverse event evaluation and reporting

Conditions or diseases which are pre-existing and chronic should not be recorded as adverse events. Changes in a chronic condition or disease that are consistent with natural disease progression are not considered adverse events. Pre-existing conditions or diseases should be documented as part of the patient's medical history.

Elective or pre-planned treatments for AF (e.g. scheduled re-ablation or cardioversion) which are part of the patient's scheduled treatment plan should not be considered adverse events. However, any events related to a procedure (including re-do procedure) or the patient's underlying AF which are not expected or indicate a worsening of condition should be reported as adverse events.

The adverse event should be recorded in standard medical terminology rather than the patient's own words when possible.

The investigator will assess each adverse event for its relationship to the study device, and whether it was anticipated in the protocol. The determination of the relationship between the adverse event and the device or procedure is made according to the following definitions:

- Definitely related (causal relationship): the adverse event was directly and clearly related to the device or procedure
- Probably related
- Possibly related
- Not related
- Unknown

Adverse events that are serious and/or unanticipated will be reviewed immediately by Labcorp and Volta employees or representatives and will also be reviewed during DSMB meetings.

All adverse events are listed in the eCRF.

All serious adverse events (SAEs, SADEs, USADEs) should be emailed to SAEIntake@labcorp.com <u>within 24 hours</u> from the become aware date. The following information should be collected:

- Nature of adverse event
- Date of onset of adverse event
- Date of resolution of adverse event, if applicable
- Statement as to why it is considered serious
- Statement as to why it is considered unanticipated, if applicable
- Statement as to the degree to which it is considered device related, and why

This document is confidential and proprietary of Volta Medical. All copy is considered uncontrolled. User must verify the revision of uncontrolled copy before use.

CONFIDENTIAL

- Results of any diagnostic tests performed
- Description of any intervention performed, or treatment administered

It is the responsibility of each investigator to report all SAEs/SADEs/USADEs to the Ethics Committee or IRB, according to national regulations and EC/IRB requirements.

Volta Medical will assure that Labcorp employee or representative will report all serious adverse events to the corresponding Competent Authorities in accordance with all applicable national regulations.

14.3 – Anticipated adverse events

Table 10 provides the list of all anticipated adverse events related to AF ablation.

Table 10: Anticipated Adverse Events

Description	Description
Abnormal ECG	Pericardial effusion
Local normal discomfort due to insertion/removal of vascular sheaths	Cardiac tamponade due to perforation
Hemorrhage and/or hematoma at sheath insertion	Nerve injury
Extremity weakness, swelling and/or pain	Air embolism
Complete AV block	Allergic reaction
Nausea/vomiting	Endocarditis
Shortness of breath/dyspnea	Esophageal-atria fistula
Feeling of chest pain, skipped beats, and/or rapid heart rate	Hemothorax
Damage to skin from prolonged exposure to x-rays	Pericarditis
New arrhythmias	Pseudoaneurysm
Ventricular arrhythmia requiring defibrillation	Pulmonary vein dissection/stenosis
Arterial injury requiring intervention	Respiratory failure
Thromboembolism	Stroke/TIA
Local/systemic infection	Valvular damage
Pneumothorax	Pleural effusion
AV fistula	Pulmonary edema
Thrombophlebitis	Anemia requiring transfusion
Pulmonary embolism	Vasovagal reaction
Myocardial infarction	Hypotension
Heart failure	Death (must be reported as SAE)

14.4 – Data Safety Monitoring Board (DSMB)

A board of independent experts will review the study data to determine the progress of the study from a safety point of view and will independently make recommendations to the Steering Committee to continue, to modify or to terminate the clinical study.

The DSMB will consider relevant background knowledge about the disease, therapies, or patient population as well as study-specific data, in order to monitor the conduct of the study to evaluate the safety of the subjects involved in the trial, to evaluate the occurrence of early unanticipated adverse effects.

CONFIDENTIAL

In particular, the board will review all the Adverse Events in order to ascertain evidence of a safety risk in continuing the study.

The DSMB is made of three independent EP experts (cf. Appendix 18.8).

XV – SUSPENSION OR PREMATURE TERMINATION OF THE CLINICAL INVESTIGATION

15.1 – Criteria and provisions

Premature, definitive, or temporary termination of all or part of the study may be decided by Ethics Committees, IRBs, Competent Authorities or the Sponsor.

If an adverse effect due to the implementation of the protocol is observed, the study may be suspended at any time by the Sponsor. If no satisfactory solution can be found to the problem, the study may be stopped completely; if a satisfactory solution is found, the study may be resumed.

Premature termination of the study may occur motivated by a general decision (Ethics Committees, IRBs, regulatory Competent Authorities, Data Safety Monitoring Board) for safety reasons related to the use of the device or study procedures.

A site may be terminated for the following reasons: failure to comply with the conditions of the study protocol and/or failure to accurately complete case report forms.

15.2 – Patient follow-up requirements

At the end of the study, medical management of patients will consist of standard post-ablation follow-up, regardless of whether the study has ended normally or prematurely.

XVI – STUDY REPORT AND PUBLICATION POLICY

After conclusion of the investigation, a comprehensive clinical and statistical report shall be written by Volta Medical in consultation with the Coordinating Investigator.

The first publication will be a full publication of all data from all sites. Any publications of the results, either in part or in total (abstracts in journals or newspapers, oral presentations, etc.) by investigators or their representatives will require pre-submission review by Volta Medical. Volta Medical is entitled to delay publication in order to obtain patent protection. For more details regarding publications, refer to the publication Agreement.

CONFIDENTIAL

This document is confidential and proprietary of Volta Medical. All copy is considered uncontrolled. User must verify the revision of uncontrolled copy before use.

XVII – BIBLIOGRAPHY

1. Kirchhof, P. *et al.* A roadmap to improve the quality of atrial fibrillation management: proceedings from the fifth Atrial Fibrillation Network/European Heart Rhythm Association consensus conference. *Europace* **18**, 37–50 (2016).
2. Morin, D. P. *et al.* The State of the Art: Atrial Fibrillation Epidemiology, Prevention, and Treatment. *Mayo Clin. Proc.* **91**, 1778–1810 (2016).
3. Rahman, F., Kwan, G. F. & Benjamin, E. J. Global epidemiology of atrial fibrillation. *Nat Rev Cardiol* **11**, 639–654 (2014).
4. Sheikh, A. *et al.* Trends in hospitalization for atrial fibrillation: epidemiology, cost, and implications for the future. *Prog Cardiovasc Dis* **58**, 105–116 (2015).
5. Verma, A. *et al.* Approaches to Catheter Ablation for Persistent Atrial Fibrillation. *N. Engl. J. Med.* **372**, 1812–1822 (2015).
6. Oral, H. *et al.* A randomized assessment of the incremental role of ablation of complex fractionated atrial electrograms after antral pulmonary vein isolation for long-lasting persistent atrial fibrillation. *J. Am. Coll. Cardiol.* **53**, 782–789 (2009).
7. Fassini, G. *et al.* Left mitral isthmus ablation associated with PV Isolation: long-term results of a prospective randomized study. *J. Cardiovasc. Electrophysiol.* **16**, 1150–1156 (2005).
8. Fink, T. *et al.* Stand-Alone Pulmonary Vein Isolation Versus Pulmonary Vein Isolation With Additional Substrate Modification as Index Ablation Procedures in Patients With Persistent and Long-Standing Persistent Atrial Fibrillation: The Randomized Alster-Lost-AF Trial (Ablation at St. Georg Hospital for Long-Standing Persistent Atrial Fibrillation). *Circ Arrhythm Electrophysiol* **10**, (2017).
9. Gaita, F. *et al.* Long-term clinical results of 2 different ablation strategies in patients with paroxysmal and persistent atrial fibrillation. *Circ Arrhythm Electrophysiol* **1**, 269–275 (2008).
10. Elayi, C. S. *et al.* Ablation for longstanding permanent atrial fibrillation: results from a randomized study comparing three different strategies. *Heart Rhythm* **5**, 1658–1664 (2008).
11. Vogler, J. *et al.* Pulmonary Vein Isolation Versus Defragmentation: The CHASE-AF Clinical Trial. *J. Am. Coll. Cardiol.* **66**, 2743–2752 (2015).

This document is confidential and proprietary of Volta Medical. All copy is considered uncontrolled. User must verify the revision of uncontrolled copy before use.

CONFIDENTIAL

12. Haïssaguerre, M. *et al.* Spontaneous initiation of atrial fibrillation by ectopic beats originating in the pulmonary veins. *N. Engl. J. Med.* **339**, 659–666 (1998).
13. Pappone, C. *et al.* Mortality, morbidity, and quality of life after circumferential pulmonary vein ablation for atrial fibrillation: outcomes from a controlled nonrandomized long-term study. *J. Am. Coll. Cardiol.* **42**, 185–197 (2003).
14. Kanj, M., Wazni, O. & Natale, A. Pulmonary vein antrum isolation. *Heart Rhythm* **4**, S73-79 (2007).
15. Atienza, F. *et al.* Real-time dominant frequency mapping and ablation of dominant frequency sites in atrial fibrillation with left-to-right frequency gradients predicts long-term maintenance of sinus rhythm. *Heart Rhythm* **6**, 33–40 (2009).
16. Atienza, F. *et al.* Comparison of radiofrequency catheter ablation of drivers and circumferential pulmonary vein isolation in atrial fibrillation: a noninferiority randomized multicenter RADAR-AF trial. *J. Am. Coll. Cardiol.* **64**, 2455–2467 (2014).
17. Jadidi, A. S. *et al.* Inverse relationship between fractionated electrograms and atrial fibrosis in persistent atrial fibrillation: combined magnetic resonance imaging and high-density mapping. *J. Am. Coll. Cardiol.* **62**, 802–812 (2013).
18. Jadidi, A. S. *et al.* Ablation of Persistent Atrial Fibrillation Targeting Low-Voltage Areas With Selective Activation Characteristics. *Circ Arrhythm Electrophysiol* **9**, (2016).
19. Narayan, S. M. *et al.* Classifying fractionated electrograms in human atrial fibrillation using monophasic action potentials and activation mapping: evidence for localized drivers, rate acceleration, and nonlocal signal etiologies. *Heart Rhythm* **8**, 244–253 (2011).
20. Nademanee, K. *et al.* A new approach for catheter ablation of atrial fibrillation: mapping of the electrophysiologic substrate. *J. Am. Coll. Cardiol.* **43**, 2044–2053 (2004).
21. Seitz, J. *et al.* AF Ablation Guided by Spatiotemporal Electrogram Dispersion Without Pulmonary Vein Isolation: A Wholly Patient-Tailored Approach. *J. Am. Coll. Cardiol.* **69**, 303–321 (2017).
22. Seitz, J. *et al.* Automated Detection of Complex Fractionated Atrial Electrograms In Substrate-Based Atrial Fibrillation Ablation: Better Discrimination with a New Setting of CARTO® Algorithm. *J Atr Fibrillation* **6**, 673 (2013).

CONFIDENTIAL

23. Monir, G. & Pollak, S. J. Consistency of the CFAE phenomena using custom software for automated detection of complex fractionated atrial electrograms (CFAEs) in the left atrium during atrial fibrillation. *J. Cardiovasc. Electrophysiol.* **19**, 915–919 (2008).
24. Seitz, J. *et al.* Artificial Intelligence Software Standardizes Electrogram-based Ablation Outcome for Persistent Atrial Fibrillation. *J. Cardiovasc. Electrophysiol.* In press (2022).
25. Mehta, C. R. & Pocock, S. J. Adaptive increase in sample size when interim results are promising: A practical guide with examples. *Statistics in Medicine* **30**, 3267–3284 (2011).

CONFIDENTIAL

XVIII – APPENDICES

18.1 – Declaration of Helsinki

18.2 – Insurance Certificate

18.3 – CE Certificates

18.4 – Patient Information and Patient Informed Consent

18.5 – Investigator Agreement

18.6 – IFU or Investigator's Brochure

18.7 – List of Investigators

18.8 – Study Boards

CONFIDENTIAL

Volta Medical

Tailored vs. Anatomical Ablation Strategy for Persistent AF
CLIPL-01-002-E

Statistical Analysis Plan

Version: 1.4

Volta Medical
65 Avenue Jules Cantini
13006 Marseille, FRANCE

CONFIDENTIAL **DO NOT COPY**

This document contains information that is confidential and proprietary. The contents of this document may not be copied without written permission from Volta Medical.

TABLE OF CONTENTS

1 PROTOCOL SUMMARY 3
2 ABBREVIATIONS 5
3 INTRODUCTION 6
4 ENDPOINT ANALYSES 6
4.1 ANALYSIS SETS 6
4.2 PRIMARY ENDPOINT 7
4.3 SECONDARY ENDPOINTS 8
4.4 EXPLORATORY ENDPOINTS 10
4.5 SAFETY ENDPOINTS 11
5 ASSESSING THE PRIMARY OUTCOME 13
6 GENERAL STATISTICAL CONSIDERATIONS 14
6.1 INTERIM ANALYSIS 14
6.2 BLANKING PERIODS 15
6.3 MISSING DATA MANAGEMENT 16
6.4 POOLING DATA ACROSS CENTERS 16
6.5 RANDOMIZATION 16
6.6 TREATMENT MASKING 16
6.7 STUDY STOPPING RULES 17
6.8 MEASURES TAKEN TO MINIMIZE BIAS 17
6.9 CORRECTION FOR MULTIPLE-TESTING 17
7 CHANGES FROM PROTOCOL 17
8 REFERENCES 18

1 Protocol Summary

**Primary Objective**	To demonstrate that a tailored ablation strategy targeting areas exhibiting spatio-temporal dispersion in association with PVI, is superior to an anatomical probabilistic ablation strategy targeting PVI alone for the treatment of persistent AF.
**Study Device/Procedure**	Tailored ablation with VX1
**Control Device/Procedure**	Anatomical Ablation
**Study Design**	Interventional, prospective, randomized, controlled, two-arm, multicenter clinical investigation
**Primary Endpoint(s)**	Freedom from documented AF, with or without AADs, 12 months after a single AF ablation procedure. Where freedom from documented AF is defined as no documented episodes of AF>30 seconds with conventional non-invasive monitoring between 3 and 12 months after index procedure. Freedom from AF is analyzed as a time-to-event comparison between survival distributions of the treatment and control arms.
**Secondary Endpoint(s)**	1. Freedom from documented AF/AT, after one or two procedures, with or without AADs, at 12 months<br>2. Freedom from documented AF/AT, after one procedure, with or without AADs, at 12 months<br>3. Composite safety endpoint of death, cerebrovascular event, or serious treatment-related adverse event at 12 months after index procedure.

**Exploratory Endpoints**	1. Freedom from documented AF, after one or two procedures, with or without AADs, at 12 months<br>2. Freedom from documented AF or AF/AT, after one procedure, or one procedure or more, without AADs, at 12 months<br>3. Freedom from documented AF or AF/AT, after one procedure, or one procedure or more, without AADs or with previously failed AAD at the same dose, at 12 months<br>4. Freedom from documented AF or AF/AT, after one procedure, or one procedure or more, with or without AADs, at 12 months, without the use of TTM<br>5. Freedom from documented symptomatic AF or AF/AT, after one procedure, or one procedure or more, with or without AADs, at 12 months<br>6. Freedom from AF or AF/AT, with or without AADs, 12 months after one procedure, or one procedure or more, for subjects with persistent AF <1 year<br>7. Quality of life measurements (SF-36 and AFEQT) at baseline, 3, 6 and 12 months post-index procedure<br>8. Incidence of peri-procedural complications including stroke, PV stenosis, cardiac perforation, esophageal injury, and death<br>9. Acute AF termination rate by ablation<br>10. Rate of conversion to sinus rhythm by ablation<br>11. Ablation procedure duration<br>12. Fluoroscopy time and dose<br>13. Mapping time<br>14. Radiofrequency (RF) time to terminate AF<br>15. Total RF time<br>16. Total delivered energy (mean power x RF duration)<br>17. Total delivered energy (mean power x RF duration) / biatrial surface<br>18. Ablation surface area to terminate AF<br>19. Total ablation surface area<br>20. Blinded baseline Voltage maps before ablation<br>21. TTM compliance (% of transmissions expected vs received at each week post index procedure)<br>22. Assessment of blind maintenance per group
**Follow-Up Schedule**	Follow-up assessments at 3, 6, and 12 months post index procedure. Holter and 12-lead ECGs will be performed at 3, 6, and 12 months. Portable trans telephonic event monitors will be recorded weekly for approximately 52 weeks.

2 Abbreviations

**AAD**	Antiarrhythmic Drug
**ADE**	Adverse Device Effect
**AE**	Adverse Event
**AF**	Atrial Fibrillation
**AFEQT**	AF Effect on Quality-of-Life Questionnaire
**AT**	Atrial Tachycardia
**CP**	Conditional Power
**ECG**	Electrocardiogram
**HR**	Hazard Ratio
**mITT**	Modified Intention-To-Treat
**PP**	Per-Protocol
**PV**	Pulmonary Vein
**PVI**	Pulmonary Vein Isolation
**QoL**	Quality-of-Life
**RF**	Radio Frequency
**SADE**	Serious Adverse Device Effect
**SAE**	Serious Adverse Event
**SAP**	Statistical Analysis Plan
**SF-36**	The Short Form (36) Health Survey
**TTM**	Trans Telephonic Monitoring

3 Introduction

This statistical analysis plan (SAP) describes the planned analyses for data collected under the Clinical Trial Protocol CLIPL-01-002 “Tailored vs. Anatomical Ablation Strategy for Persistent AF”, last revised 06 July 2022 (Revision E). Specified analyses may be used for scientific presentations and/or manuscripts, and regulatory submissions. The primary analysis will be based on the data through 12 months post-index procedure.

If there are any discrepancies between this SAP and the study protocol, the SAP shall prevail.

4 Endpoint Analyses

4.1 ANALYSIS SETS

The analysis sets making up this study will include the following:

4.1.1 The Safety Population

The Safety population will include all randomized Subjects who underwent their first ablation procedure.

4.1.2 The Modified Intent-to-Treat Population (mITT)

The modified Intention-To-Treat (mITT) set will include all randomized subjects except for:

- Subjects who are deemed ineligible after randomization or who do not have any AF ablation, and
- Subjects lost to follow-up during the 3-month blanking period.

Subjects in the Tailored group for whom AF cannot be induced and who undergo conventional ablation will be included in the mITT analysis.

Subjects who complete at least their 3-month follow-up visit but who are lost to follow-up before the 12-month follow-up visit will be included in the mITT analysis.

Subjects who undergo a 3rd ablation procedure will be included in the analyses but the data will be censored at the time of the 3rd procedure for multiple procedure endpoints.

4.1.3 The Per-Protocol Population (PP)

The Per-Protocol (PP) analysis set will include only those subjects of either given treatment arm defined as the treatment actually received at the start of the study, and will consist of subjects who met all the inclusion criteria, none of the exclusion criteria, were successfully treated with an index procedure, had no important protocol deviations, and attended the scheduled follow-up visits (did not miss more than one mandatory Holter recording, and TTM compliance >50%).

The PP analysis set will also be used to evaluate endpoints already evaluated using the mITT as the primary analysis set, thus serving as supportive analyses to that of the mITT efficacy analysis.

4.2 PRIMARY ENDPOINT

Freedom from documented AF, 12 months after a single index ablation procedure to be understood as a single AF ablation procedure (i.e. including redo procedures for non-AF indications).

4.2.1 Hypotheses

Freedom from documented AF after 12 months will be higher in the tailored group than the anatomic group.

The null and alternative hypotheses are as follows:

$$H_0: S_1(t) = S_2(t)$$

$$H_A: S_1(t) > S_2(t),$$

where S1(t) and S2(t) represent the survival functions for the Tailored and Anatomical arms, respectively, at any given time t.

4.2.2 Sample Size

The primary endpoint is freedom from AF, 12 months after a single ablation procedure. Assuming that 62% of the Subjects in the anatomic group will be free from AF at the end of follow-up1,2, that at least 77% of the Subjects of the tailored group will be free from AF at the end of follow-up (i.e., Hazard Ratio (HR) 0.547), then 292 Subjects (146 in each group) are required to show that time to AF is significantly different between groups (Log-Rank test for a one-sided superiority trial, significance level α=0.025, statistical power of 80%, under the assumption that the hazard rates are proportional). Assuming a drop-out rate of 22% (no index ablation performed or loss to follow up), 374 Subjects are required overall (187 in each group). Following 50% of subjects having a primary endpoint collected, the sample size will be re-estimated using the Mehta-Pocock conditional power (CP) approach.

4.2.3 Primary Analysis

The primary endpoint will be analyzed using a Cox Proportional hazards model to conduct a time-to-event comparison (of hazard rates from risk of AF recurrence at a time t) between the Tailored (treatment) and Anatomical (control) groups. The model will include an indicator for group (study arm), a dichotomous effect for AF duration (persistent AF ≥ 6 months; persistent AF < 6 months), and applying right-censoring of subjects lost to follow-up. The model will be used to estimate the hazard ratio of AF occurrence between the Tailored arm compared to that of the Anatomical arm, along with a two-sided 95% CI and p-value for the HR on behalf of the group predictor. The actual null hypothesis of the Primary Endpoint will

be tested with a one-sided Log-Rank test at a significance level of α=0.025. The null hypothesis will be rejected if the p-value is < 0.025, and the survival distribution of the Tailored arm will be considered superior relative to that of the Anatomical arm. All of the outputs described above will be generated under the same single Cox Proportional hazards model, which will be conducted in the mITT population. A Kaplan-Meir plot will be generated to visually display the survival distributions of the Tailored and Anatomical arms, respectively to accommodate the p-value from their comparison.

4.2.4 Sensitivity Analyses of Primary Endpoint

The Primary Endpoint Analysis will additionally be conducted in the PP population.

4.2.5 Other analyses on primary endpoint

Primary analyses will be repeated with the following changes in the Cox Proportional Hazards model:

Without AF duration fixed effect. With AF duration fixed effect numerically expressed as the logarithm of the AF duration (in months) instead of the categorical definition. The format of the AF duration variable (dichotomous vs. numerically expressed as the log of months) will be compared between the models with which each is fit.

4.2.6 Subgroup Analyses

The following subgroup analyses will be performed following the approach described for the primary analysis:

1. All subjects with AF duration within the following categories: < 6 months, ≥ 6 months, < 12 months, and ≥ 12 months

2. AF presentation at index procedure (spontaneous AF, induced AF)

3. With/without AADs

4. Patient gender (male and female)

4.3 SECONDARY ENDPOINTS

The secondary endpoints are as follow:

1. Freedom from AF/AT after one or two procedures at 12 months following index procedure.
2. Freedom from AF/AT after a single procedure at 12 months following index procedure.
3. The safety endpoint is a composite endpoint, the events of which it is comprised include that of death, cerebrovascular event, or serious treatment-related adverse event at the 12-month timepoint since initial index procedure (this endpoint is inclusive of the Blanking Period – marked by the 3 months immediately following the initial index procedure undergone by each enrolled subject).

4.3.1 Secondary Analysis

The secondary endpoint analyses will consist of the following:

Two tests of the primary endpoint in the mITT population, but expanded to be defined as freedom from AF/AT, with or without AADs, 12 months after one or two procedures. A superiority hypothesis will be tested using a Cox proportional hazards model with randomization group, AF duration, and AF/AT strata as fixed effects, with a one-sided alpha level of 0.025:

Both analyses under will be evaluated according to the following hypotheses:

$$H_0: S_1(t) = S_2(t)$$

$$H_A: S_1(t) > S_2(t),$$

where $S_1(t)$ and $S_2(t)$ represent the survival functions for the Tailored and Anatomical arms, respectively, at any given time *t*. If the null hypotheses for each of the tests is rejected, the following superiority statements will be considered on behalf of each endpoint:

1. Tailored ablation is superior to anatomical with respect to freedom from AF/AT, with or without AADs, 12 months after one or two procedures.

2. Tailored ablation is superior to anatomical with respect to freedom from AF/AT, with or without AADs, 12 months after one procedure.

Similarly to the primary endpoint analyses, Cox Proportional hazards regression models will be fit and assessed for goodness-of-fit. Then to test each null hypothesis, one-sided Log-Rank Tests will be. Just like under the primary analysis, the p-value from the one-sided Log-Rank Test run under each Cox Proportional hazards model will ultimately be what is used to either reject ($p < 0.025$) or fail to reject ($p \geq 0.025$) the null hypotheses above. Both Cox models will be conducted in the mITT population.

Kaplan-Meier plots will be generated for each endpoint, to visually display the differences among the survival distributions of the Tailored and Anatomical arms, respectively, with each plot accommodated by the p-value generated from their respective one-sided Log-Rank test.

3. The composite adverse event (AE) rate defined as the number of AEs per month occurring in each treatment arm from time of index procedure through to 12-month follow-up, where composite AEs are defined by either death, a cerebrovascular event, or any serious procedure-related adverse event, will be conducted. in the Safety population (see Section 4.5, Safety Endpoints below).

4.3.2 Sensitivity Analyses of Secondary Survival Endpoints

The first two Secondary Endpoint Analyses will additionally be conducted in the PP population.

4.4 EXPLORATORY ENDPOINTS

The following endpoints compared in anatomical vs. tailored groups will be considered exploratory:

1. Freedom from documented AF, after one or two procedures, with or without AADs, at 12 months
2. Freedom from documented AF or AF/AT, after one procedure, or one procedure or more, without AADs, at 12 months
3. Freedom from documented AF or AF/AT, after one procedure, or one procedure or more, without AADs or with previously failed AAD at the same dose, at 12 months
4. Freedom from documented AF or AF/AT, after one procedure, or one procedure or more, with or without AADs, at 12 months, without the use of TTM
5. Freedom from documented symptomatic AF or AF/AT, after one procedure, or one procedure or more, with or without AADs, at 12 months
6. Freedom from AF or AF/AT, with or without AADs, 12 months after one procedure, or one procedure or more, for subjects with persistent AF <1 year.
7. Freedom from AF or AF/AT, with or without AADs, 12 months after one procedure, or one procedure or more, for subjects with AF ≥ 6 months (including long-standing persistent AF)
8. Estimated AF or AF/AT burden, with or without AADs, after one procedure, or one procedure or more
9. Quality of life measurements (SF-36 and AFEQT) at baseline, 3, 6 and 12 months post-index procedure
10. Incidence of peri-procedural complications including stroke, PV stenosis, cardiac perforation, esophageal injury, and death
11. Acute AF termination rate by ablation
12. Rate of conversion to sinus rhythm by ablation
13. Ablation procedure duration
14. Fluoroscopy time and dose
15. Mapping time
16. Radiofrequency (RF) time to terminate AF
17. Total RF time
18. Total delivered energy (mean power x RF duration)
19. Total delivered energy (mean power x RF duration) / biatrial surface
20. Ablation surface area to terminate AF
21. Total ablation surface area
22. Blinded baseline Voltage maps before ablation
23. TTM compliance (% of transmissions expected vs received at each week post index procedure)

4.4.1 Exploratory Endpoint Analysis

Endpoints 1 – 8 will be tested using a Cox proportional hazards model with randomization group and AF strata as fixed effects. Hypotheses will be tested in the following order, and each at a less stringent one-sided alpha level of 0.05, given the exploratory nature of these analyses)

Endpoint 9 will be tested by comparing the QoL score at each visit between tailored and anatomical ablation using a t-test for difference in means. A Shapiro-Wilks test will be used to assess whether the data meets normality assumptions for parametric. Should the normality assumption be violated, a non-parametric method such as the Wilcoxon test will be used instead. The effect of time will also be assessed using a generalized linear mixed model for repeated measures.

Endpoints 10 – 21 and 23 will be tested by comparing tailored and anatomical ablation groups. using a t-test for differences in means. Should the normality assumption be violated, a non-parametric method such as the Wilcoxon test will be used instead.

Endpoint 22 will not undergo any statistical analysis, but the data collected (blinded baseline voltage maps prior to ablation) will be provided to the sponsor in a dataset / spreadsheet format to enable any post-hoc analysis that may be desired.

4.5 SAFETY ENDPOINTS

The safety endpoint is a composite endpoint at 12 months of the following: death, cerebrovascular events, or serious treatment-related adverse event. Because the rates of this endpoint which are observed under each of the study arms may or may not prove to be relatively rare (< 5% within a given arm), two methods of analysis are provided, the one which will ultimately be used to analyze the safety endpoint will depend on its rate of occurrence, as observed across the two study groups. The safety endpoint analysis, regardless of the method employed, will be conducted in the Safety population. The same analysis will also be conducted in the mITT and PP populations, since if rare occurrence of the safety endpoint is observed and the few occurrences observed happen to all occur within the mITT/PP populations, then the smaller subject sample sizes will serve to increase the rates/proportions observed in each study arm.

METHOD 1 (safety endpoint occurrence > 5% in each study arm):

The safety analysis will be to compare the AE composite rate per month (defined as the 4th secondary endpoint in Section 1 Protocol Summary) under the Tailored (treatment) arm against the Anatomical (control) arm AE composite rate per month from the time of index procedure until the 12-month follow-up. A two-sided 95% CI will be generated for the rate ratio (λ_t/λ_c) comparing the AE composite rate for the two study arms.

Let λ_c and λ_t denote the rate of composite adverse events for the Anatomical and the Tailored treatment arms, respectively. Given a non-inferiority margin δ, the null and alternative hypotheses are:

$$H_0: \frac{\lambda_t}{\lambda_c} \geq 1 + \delta$$

$$H_1: \frac{\lambda_t}{\lambda_c} < 1 + \delta$$

λ_t, λ_c, and $\frac{\lambda_t}{\lambda_c}$ will be estimated with a linear fixed effects model with a log link function and a Poisson distribution for the number of adverse events (referring only to those defined under the composite AE endpoint). The outcome of the model is the number of composite AEs for each subject enrolled and randomized to one of the study arms. The model will

include an indicator variable for Study Arm, an offset for the log of the number of weeks each subject remained enrolled in the study will be added (the date of each subject's index procedure will be regarded as their first day on the study).In the event the model fails to converge, it will be refit with a Negative Binomial distribution.

H_0 is rejected if the upper limit of the 95% confidence interval for $\frac{\lambda_t}{\lambda_c}$ is less than $1 + \delta$. If H_0 is rejected, non-inferiority is demonstrated. Based upon the number of months and the expected rate of adverse events, the current study design is under-powered to detect a clinically meaningful (less than 5%) non-inferiority margin for the Tailored treatment arm. Because the null hypothesis test must be conducted under a specified non-inferiority margin, the analysis will use $\delta = 0.05$.

METHOD 2 (safety endpoint occurrence (≤ 5% in each study arm):

If both model approaches fail to converge, most likely due to the incidence of the composite AE rates being relatively rare (less than or equal to 5%) under both study arms, then the composite AE endpoint will be treated as a frequency instead of a rate, such that P_1 will be used to denote the proportion defined by the number of composite AEs out of total subjects in the Tailored (treatment) arm and P_2 will be used to denote the proportion defined by the number of composite AEs out of total subjects in the Anatomical (control) arm. The difference in proportions ($P_1 - P_2$) will be reported along with a two-sided exact 95% CI using an exact test statistic with continuity correction if not already defaulted (a Fisher's Exact test may be used if CI can be generated from its statistic for the difference of proportions estimate). Given a non-inferiority margin δ, the null and alternative hypotheses are as follow:

$$H_0: p_1 - p_2 \geq \delta$$
$$H_1: p_1 - p_2 < \delta$$

If the upper bound of the 90% CI for ($P_1 - P_2$) is less than $\delta = 0.025$, the null hypothesis is rejected, and non-inferiority is demonstrated (2.5% has been allocated as a clinically meaningful non-inferiority margin in the absence of literature). In the event that the composite AEs have a zero occurrence in both study arms, a confidence interval for the difference of proportions cannot be computed, in which case 95% exact CIs for P_1 and P_2 will be reported, and non-inferiority will be considered demonstrated.

4.5.1 Other Adverse Events

All adverse events will be summarized through 12 months. The proportions of subjects with events will be reported. No imputation for missing data is planned.

Tabular summaries by treatment group of all adverse events, adverse device effects, serious adverse events, and serious adverse device effects will be presented.

An adverse event (AE) is defined as any untoward medical occurrence, unintended disease or injury or untoward clinical signs (including an abnormal laboratory finding) in subjects,

users or other persons, whether or not related to the investigational medical device. An Adverse Device Effect (ADE) is defined as an adverse event related to the use of an investigational medical device. A serious adverse event (SAE) is an adverse event that:

a) led to a death

b) led to a serious deterioration in the health of the subject, that either resulted in: - a life-threatening illness or injury, or - a permanent impairment of a body structure or a body function, or - in-patient or prolonged hospitalization, or -medical or surgical intervention to prevent life threatening illness or injury or permanent impairment to a body structure or a body function.

c) led to fetal distress, fetal death or a congenital abnormality or birth defect. A serious adverse device effect (SADE) is an adverse device effect that has resulted in any of the consequences characteristic of a serious adverse event.

The investigator will assess each adverse event for its relationship to the study device, and whether it was anticipated in the protocol. The determination of the relationship between the adverse event and the device is made according to the following definitions:

- Definitely related (causal relationship)
- Probably related
- Possibly related
- Not related
- Unknown.

Acute peri-procedural complications (related to the study procedure and occurring within one week of the ablation procedure), will be specifically analyzed as part of the exploratory endpoints.

5 Assessing the Primary Outcome

Freedom from documented AF between 3 and 12 months following the index ablation procedure will be defined as no documented episodes of AF > 30 seconds with conventional non-invasive monitoring.

The following scenarios result in primary endpoint failure:

1) A repeat procedure where AF is confirmed during the procedure (Patient is in spontaneous AF at the beginning of the procedure)

2) A 30-second or greater episode of AF is documented on a 24-hour Holter

3) A 30-second of AF on a weekly TTM transmission confirmed by 30-second of AF on same (but different day) or next week TTM transmission. Two AF transmissions on the same day do not count as a confirmed recurrence.

4) Since 12-Lead ECGs are 10 seconds in duration, an AF episode of >30 seconds will be documented via 12-lead EGC under only certain conditions (AF symptoms, multiple ECGs recorded at a single visit showing AF, confirmation of single Kardia in AF in same, previous or next week).

TTM will be measured weekly by the patient via a portable ECG device (KardiaMobile, AliveCor). The recordings will be automatically uploaded for blind review by the ECG core lab.

24-hour Holter monitor assessments will be performed prior to or subsequent to each in-office visit at 3, 6, and 12 months post-index procedure.

Diagnostic 12-lead ECGs will be performed at each in-office visit at 3, 6, and 12 months following the index procedure.

6 General Statistical Considerations

6.1 INTERIM ANALYSIS

This trial will employ an adaptive design for the blinded re-estimation of the sample size. To ensure the study is adequately powered for the superiority comparison for the primary outcome measure, the assumptions regarding the rates of AF in both study arms will be verified after 50% of the originally planned total patient sample have complete primary endpoint information. A conditional power (CP) approach for sample-size re-estimation using the Mehta-Pocock approach will be utilized.[3] Following this interim assessment, the sample size may only remain the same or be increased. A third-party, independent statistician will perform this analysis to minimize operational bias. This analysis will occur after approximately 50% of the originally planned subjects a completed/analyzable primary endpoint. The statistician will assess the conditional study power assuming that the hazard ratio remains the value estimated at this analysis. The conditional power will be categorized into one of the three following zones:

Favorable: CP ≥ 80%
Promising: 36% ≤ CP < 80%
Unfavorable: CP < 36%

The lower bound on the promising results is selected from Mehta and Pocock as a function of the target power, timing of the sample size re-estimation, and maximum sample size. Selection of this boundary in particular does not inflate the overall Type I error rate.
After the analysis, the statistician will provide a recommendation to the Steering Committee and Volta Medical clinical team:

- In the event that the conditional power is found to be in the Unfavorable zone, the study will continue to the originally planned sample. The independent statistician will report ONLY that the trial will continue as originally planned.
- In the event that the conditional power assessment is Promising, the sample size will be increased to 100 total subjects (an additional 50 maximum per treatment arm, predetermined by Volta). The independent statistician will report that the sample size should be increased and by how many subjects (in regard to the 292 subjects originally planned).
- In the event that the conditional power is found to be in the Favorable zone, the study will continue to the originally planned sample. The independent statistician will report ONLY that the trial will continue as originally planned.

Also, the following information will be shared with senior, non-operational management staff that have a strict business need to review and act on the results:

- The recommendation (sample size increase or continue as originally planned)

- The rate of freedom from AF for each arm for the overall interim analysis population

- The rate of freedom from AF for each arm for two subgroups depending on the AF type (persistent AF and long-standing persistent AF)

None of the clinical operational staff or study investigators will have access to the interim data on which the analysis is based to further minimize operational bias. The scientific soundness of the study is not likely to be impacted as the only potential change to the study as a result of this analysis is an increase in sample size.

6.2 BLANKING PERIODS

After the index procedure, a 3-month blanking period + tolerance of 1 week (98 days) will be applied for all efficacy endpoints, during which recurrences of AF/AT (excluding repeat procedure) will not be counted.

If a repeat procedure is performed during the 3-month blanking period (and patient is in spontaneous AF), the recurrence and the repeat procedure will be counted as occurring at 3 months.

If a repeat procedure is performed after the end of the 9th month, the procedure will not be considered for multiple procedures endpoints.

After a repeat procedure, a 2-week blanking period (14 days) will be applied, during which AF/AT recurrences will not be counted. Any re-repeat procedures (repeat procedure #2) will result in the data being censored at the time of the re-repeat procedure.

Note: for the purpose of counting months to establish blanking period boundaries in the study, one month will be considered equal to 30.4 days.

6.3 MISSING DATA MANAGEMENT

Subjects who miss a study visit should be contacted immediately by the Investigational site to determine the reason for the missed visit and to reschedule the visit as soon as possible to meet the study visit window. If the subject cannot be located after 3 attempts by phone, email and/or letter, then the subject will be considered lost to follow-up. All attempts to contact the subjects will be documented and retained in the subject study record. Regarding the main judgement criteria, data will be censored if the patient is lost to follow-up. As the burden of study participation does not differ between the tailored and the anatomical arm, we expect all study losses to be non-differential with respect to treatment assignment. Thus, our results will not be biased because of these losses. Additional supportive analyses will be performed on the primary endpoint to assess the effect of missing data. For missing demographic data, the data will not be replaced because it has no weight on the main judgment criterion. A rigorous monitoring is expected; any missing data will be filled in during these visits.

6.4 POOLING DATA ACROSS CENTERS

The analyses will be presented using data pooled across study sites. Summary statistics for the rate of freedom from AF will be calculated by site. A formal assessment of site-to-site heterogeneity will be assessed using a linear mixed-effects binomial regression model with a logit link function for the event of AF recurrence (corresponding to a difference of proportions analysis). The outcome of the model will be the binary status of AF recurrence per subject (0=Subject did not experience AF recurrence, 1=AF recurrence documented on behalf of subject). The model will include an indicator variable for study arm, AF duration as a fixed effect, site as a fixed effect, and a study arm-by-site interaction term. Within the model results, if the study arm-by-site interaction coefficient has a p-value <0.15, it will be regarded as there being heterogeneity detected between sites. Lastly, poolability by geography (US vs. OUS) will be performed based on linear mixed-effects binomial regression as described above. As US subjects will only be patients with AF duration of 3 months to < 12 months, only OUS patients with AF <12 months will be included in the poolability by country assessment.

6.5 RANDOMIZATION

After successful enrollment, the patient will be automatically randomized in a 1:1 ratio to one of two arms through the eCRF system: “Anatomical” arm (PVI) or “Tailored” arm (Dispersion + smart-PVI). Randomization will be stratified in order to balance the treatment arms in terms of subjects with persistent AF (< 12 months) and long-standing persistent AF (≥ 12 months). Subjects will not be informed of their randomization assignment.

6.6 TREATMENT MASKING

Special care will be taken to ensure that the patient will be blinded to his/her randomization assignment throughout the study. Patient unblinding will be documented as a non-important deviation. Moreover, a blind rhythm monitoring is planned: all ECG recordings for long-term effectiveness endpoints (Holter, TTM and 12-lead ECG) will be analyzed and adjudicated blindly by an independent ECG core lab. Subjects will be asked at the 12-month visit to guess which arm of the study they are in.

6.7 STUDY STOPPING RULES

Premature termination of the study may occur motivated by a general decision (Ethics Committees, Institutional Review Boards, regulatory Competent Authorities, Data Safety Monitoring Board, Steering Committee) for safety reasons related to the use of the device or study procedures.

6.8 MEASURES TAKEN TO MINIMIZE BIAS

Several measures are incorporated into the study design to help minimize study bias as follows:

1) This is a multi-center trial to help ensure that investigator or site or subject enrollment bias is minimized. Selection of subjects will be made from the Investigator's usual subject load. Consecutively eligible subjects should be enrolled into the study.
2) This document specifies appropriate statistical methods to ensure that bias is minimized.
3) Standardized and validated case report forms will be used to collect data during the study.
4) Steps to ensure blinding to treatment received have been taken (see Section 6.6)

6.9 CORRECTION FOR MULTIPLE-TESTING

For the two secondary efficacy endpoints, the same variable will be observed after up to two procedures (one or two procedures), or after a single procedure. Given the potential number of patients receiving a second ablation procedure (between 22% and 33%[1]), the variable for both secondary efficacy endpoints will be the same for most patients, meaning that the two variables will be highly correlated. The third secondary endpoint relates to safety, and is therefore of a different nature. Consequently, no correction for multiple testing will be applied for secondary endpoints.

7 Changes from protocol

Primary endpoint hypothesis in the protocol was wrongly based on the effect size observed in a previous study and used in the sample size estimation (delta 15%, HR = 0.547). The study is powered to detect such an effect size. The tested hypotheses were corrected to H0: $S1(t) = S2(t)$ and HA: $S1(t) > S2(t)$.

The gatekeeping approach for secondary endpoints was abandoned. There are 3 secondary endpoints of different nature (2 highly-correlated efficacy, 1 safety) and therefore no multiple-testing correction will be applied.

8 References

1. Verma, A. et al. “Approaches to catheter ablation for persistent atrial fibrillation.” *New England Journal of Medicine* 372 (2015): 1812–1822.

2. Fink, T. et al. “Stand-alone pulmonary vein isolation versus pulmonary vein isolation with additional substrate modification as index ablation procedures in patients with persistent and long-standing persistent atrial fibrillation: the randomized alster-lost-AF Trial (ablation at St. Georg hospital for long-standing persistent atrial fibrillation).” *Circ Arrhythm Electrophysiol* 10, (2017).

3. Mehta, Cyrus R., and Stuart J. Pocock. "Adaptive increase in sample size when interim results are promising: a practical guide with examples." *Statistics in medicine* 30.28 (2011): 3267-3284.
