# Supplementary material for: Artificial intelligence for individualized treatment of persistent atrial fibrillation: a randomized controlled trial
Source: Nat Med. 2025 Feb 14;31(4):1286–93. doi: 10.1038/s41591-025-03517-w (PMC12003177; doi:10.1038/s41591-025-03517-w)
Supplement: Supplementary file 2 — Reporting Summary [file 41591_2025_3517_MOESM2_ESM.pdf]

nature portfolio

Corresponding author(s): Isabel Deisenhofer

Last updated by author(s): Nov 28, 2024

Reporting Summary

Nature Portfolio wishes to improve the reproducibility of the work that we publish. This form provides structure for consistency and transparency in reporting. For further information on Nature Portfolio policies, see our Editorial Policies and the Editorial Policy Checklist.

Statistics

For all statistical analyses, confirm that the following items are present in the figure legend, table legend, main text, or Methods section.

n/a	Confirmed	
☐	☒	The exact sample size (*n*) for each experimental group/condition, given as a discrete number and unit of measurement
☐	☒	A statement on whether measurements were taken from distinct samples or whether the same sample was measured repeatedly
☐	☒	The statistical test(s) used AND whether they are one- or two-sided<br>*Only common tests should be described solely by name; describe more complex techniques in the Methods section.*
☒	☐	A description of all covariates tested
☐	☒	A description of any assumptions or corrections, such as tests of normality and adjustment for multiple comparisons
☐	☒	A full description of the statistical parameters including central tendency (e.g. means) or other basic estimates (e.g. regression coefficient) AND variation (e.g. standard deviation) or associated estimates of uncertainty (e.g. confidence intervals)
☐	☒	For null hypothesis testing, the test statistic (e.g. *F*, *t*, *r*) with confidence intervals, effect sizes, degrees of freedom and *P* value noted<br>*Give P values as exact values whenever suitable.*
☒	☐	For Bayesian analysis, information on the choice of priors and Markov chain Monte Carlo settings
☒	☐	For hierarchical and complex designs, identification of the appropriate level for tests and full reporting of outcomes
☒	☐	Estimates of effect sizes (e.g. Cohen's *d*, Pearson's *r*), indicating how they were calculated

Our web collection on statistics for biologists contains articles on many of the points above.

Software and code

Policy information about availability of computer code

Data collection	iMednet EDC system versions 1.204.0 to 1.241.4 hosted by Mednet Solutions and Atrium version 9.1.0 by Banook Group.
Data analysis	Volta AF-Xplorer (VX1) Software version 1.4 (Volta Medical), SAS Software version 9.4 (SAS Institute), and R Statistical Software version 4.3.2 (R Core Team).

For manuscripts utilizing custom algorithms or software that are central to the research but not yet described in published literature, software must be made available to editors and reviewers. We strongly encourage code deposition in a community repository (e.g. GitHub). See the Nature Portfolio guidelines for submitting code & software for further information.

Data

Policy information about availability of data

All manuscripts must include a data availability statement. This statement should provide the following information, where applicable:

- Accession codes, unique identifiers, or web links for publicly available datasets
- A description of any restrictions on data availability
- For clinical datasets or third party data, please ensure that the statement adheres to our policy

All supporting data are available within the article and the Supplementary Information. Source data will not be shared due to patient privacy obligations applicable to the sponsor under the European data privacy regulation (GDPR) and in particular due to the obligations of privacy to which the Sponsor committed itself in the informed consent form signed by the patient.

Research involving human participants, their data, or biological material

Policy information about studies with human participants or human data. See also policy information about sex, gender (identity/presentation), and sexual orientation and race, ethnicity and racism.

Reporting on sex and gender	Baseline patient characteristics, including sex, are provided in the manuscript. Findings do not apply to one sex or gender, and sex and gender were not prespecified in the study design. Self-reporting (e.g. discussion between patient and treating physician) was used to determine patient sex. Informed consent was obtained from all patients prior to enrollment in the study. Sex- or gender- based analyses are not provided, as it was not the main purpose of the clinical study.
Reporting on race, ethnicity, or other socially relevant groupings	All baseline patient characteristics (age, sex, comorbidities, years of diagnosis of persistent AF) are provided in Table 1 of the manuscript.
Population characteristics	All baseline patient characteristics (age, sex, comorbidities, years of diagnosis of persistent AF) are provided in Table 1 of the manuscript.
Recruitment	Participants were recruited by participating institutions in the clinical study based on direct conversations between healthcare providers and patients. All participants were required to meet inclusion/exclusion criteria prior to undergoing their index procedure. Randomization between the investigational and control arms serves as a method of experimental control for human clinical trials to reduce selection bias introduced by the sampling methods. All subjects were blinded to their treatment assignment.
Ethics oversight	FDA approval was obtained. Ethical approval was obtained from Western IRB (WIRB), Ascension St. Vincent IRB, Rhode Island Hospital IRB (United States), CPP SUD-EST IV (France), Technische Universität München (TUM) Ethikkommission, Landesärztekammer Baden-Württemberg Ethikkommission (Germany), OLV Ziekenhuis vzw Ethisch Comite (Belgium), and Brabant Medical Ethics Committee (Netherlands).

Note that full information on the approval of the study protocol must also be provided in the manuscript.

Field-specific reporting

Please select the one below that is the best fit for your research. If you are not sure, read the appropriate sections before making your selection.

☒ Life sciences ☐ Behavioural & social sciences ☐ Ecological, evolutionary & environmental sciences

For a reference copy of the document with all sections, see nature.com/documents/nr-reporting-summary-flat.pdf

Life sciences study design

All studies must disclose on these points even when the disclosure is negative.

Sample size	Sample size estimations were based on the assumptions that 77% of the subjects in the Tailored group will be free from atrial fibrillation 12 months after a single ablation procedure, vs. 62% in the Anatomical group, corresponding to a Hazard Ratio of 0.547. Using a log-rank test for superiority of the Tailored group vs. the Anatomical group and a randomization ratio of 1:1, a total of 292 subjects were needed for the study to have a power of 80% at a one-sided alpha level of 0.025. Assuming a dropout rate of 22% (no index ablation performed or loss to follow-up), 374 subjects were required.
Data exclusions	No data was excluded from the analysis.
Replication	All study data entered into the clinical study database was 100% source data verified by clinical study monitors. Study data was monitored against source documentation, and queried for accuracy of data collection. A statistician from the CRO performed analyses of the primary and several secondary endpoints reported in the manuscript, according to the statistical analysis plan. An internal statistician and a third independent statistician both reproduced the different analyses. A blinded and independent ECG core lab adjudicated all arrhythmia monitoring transmissions, and an independent data safety monitoring board reviewed safety events and monitored the study conduct. All study measurements were taken from distinct samples, with each trial participant as an independent sample.
Randomization	Subjects were randomly assigned in a 1:1 ratio to either the control "Anatomical" group (pulmonary vein isolation-only) or the investigational "Tailored" group undergoing a tailored cardiac ablation procedure targeting artificial intelligence-identified electrogram spatio-temporal dispersion in addition to pulmonary vein isolation (Fig. 1 and Extended Data Fig. 1). Randomization was performed using the method of random permuted block. The block sizes were randomly chosen as 2, 4 and 6. Randomization was stratified according to atrial fibrillation type and site.
Blinding	Since investigator blinding was not possible, the randomization outcome was communicated to the operator after patient enrollment. The core lab adjudicating all ECG transmissions was blinded to the randomization outcome. Patients were also blinded to the randomization outcome.

Reporting for specific materials, systems and methods

We require information from authors about some types of materials, experimental systems and methods used in many studies. Here, indicate whether each material, system or method listed is relevant to your study. If you are not sure if a list item applies to your research, read the appropriate section before selecting a response.

Materials & experimental systems

n/a	Involved in the study
☒	☐ Antibodies
☒	☐ Eukaryotic cell lines
☒	☐ Palaeontology and archaeology
☒	☐ Animals and other organisms
☐	☒ Clinical data
☒	☐ Dual use research of concern
☒	☐ Plants

Methods

n/a	Involved in the study
☒	☐ ChIP-seq
☒	☐ Flow cytometry
☒	☐ MRI-based neuroimaging

Clinical data

Policy information about clinical studies

All manuscripts should comply with the ICMJE guidelines for publication of clinical research and a completed CONSORT checklist must be included with all submissions.

Clinical trial registration

NCT04702451

Study protocol

The full trial protocol is available in the Supplementary Material.

Data collection

Patients were treated between February 2021 and December 2022, and followed for one year at their respective hospital institutions until December 2023.

Outcomes

The primary efficacy endpoint was freedom from documented atrial fibrillation, with or without AADs, 12 months after a single index ablation procedure, defined as no documented episodes of AF > 30 seconds with conventional non-invasive monitoring (Holters, TTM and 12-lead ECG recordings) between 3 and 12 months after index procedure. Difference of freedom from arrhythmia between the arms has been analyzed as comparison of survival distributions of the two arms in order to take into account the time to arrhythmia recurrence and the censored data.

The secondary efficacy endpoints were: (1) Freedom from any documented arrhythmia, after one or two procedures, with or without AADs, at 12 months. (2) Freedom from any documented arrhythmia, after one procedure, with or without AADs, at 12 months.

Primary and secondary outcomes measures were adjudicated by a blinded and independent ECG core lab.

The safety endpoint was a composite endpoint at 12 months (death, cerebrovascular events, or serious treatment-related adverse event) and was tested for non-inferiority.

Safety events were assessed by the principal investigator and reviewed by the data safety monitoring board.

Plants

Seed stocks

Report on the source of all seed stocks or other plant material used. If applicable, state the seed stock centre and catalogue number. If plant specimens were collected from the field, describe the collection location, date and sampling procedures.

Novel plant genotypes

Describe the methods by which all novel plant genotypes were produced. This includes those generated by transgenic approaches, gene editing, chemical/radiation-based mutagenesis and hybridization. For transgenic lines, describe the transformation method, the number of independent lines analyzed and the generation upon which experiments were performed. For gene-edited lines, describe the editor used, the endogenous sequence targeted for editing, the targeting guide RNA sequence (if applicable) and how the editor was applied.

Authentication

Describe any authentication procedures for each seed stock used or novel genotype generated. Describe any experiments used to assess the effect of a mutation and, where applicable, how potential secondary effects (e.g. second site T-DNA insertions, mosiacism, off-target gene editing) were examined.
